# Supplementary material for: Antiprotozoal Aminosteroids from Pachysandra terminalis
Source: Molecules. 2025 Feb 27;30(5):1093. doi: 10.3390/molecules30051093 (PMC11902257; doi:10.3390/molecules30051093)
Supplement: Supplementary file 1 [file molecules-30-01093-s001.zip › molecules-3458136-supplementary.pdf]

Supplementary Materials

Antiprotozoal Aminosteroids from *Pachysandra terminalis*

Lizanne Schäfer [1], Monica Cal [2,3], Marcel Kaiser [2,3], Pascal Mäser [2,3] and Thomas J. Schmidt [1,*]

[1] University of Münster, Institute of Pharmaceutical Biology and Phytochemistry (IPBP), PharmaCampus Corrensstraße 48, D-48149 Münster, Germany; l_scha57@uni-muenster.de
[2] Swiss Tropical and Public Health Institute (Swiss TPH), Kreuzstrasse 2, CH-4123 Allschwil, Switzerland; monica.cal@swisstph.ch (M.C.); marcel.kaiser@swisstph.ch (M.K.); pascal.maeser@swisstph.ch (P.M.)
[3] University of Basel, Petersplatz 1, CH-4003 Basel, Switzerland
* Correspondence: thomschm@uni-muenster.de; Tel.: +49-251-833-3378

Table S1: IC_{50}-values (in µg/mL) of the in vitro antiprotozoal (*Plasmodium falciparum*, *Pf*, *Trypanosoma brucei rhodesiense*, *Tbr*) as well as cytotoxic activity (L6 rat skeletal myoblasts) of the CPC fractions from aerial parts of *Pachysandra terminalis*

Figure S1-S10: Spectral data (LC/MS, ^{1}H NMR, ^{13}C NMR, 2D NMR (COSY, HSQC, HMBC), UV and CD) of 9-(*N*,*N*-dimethyl)-5-megastigmen-1-one (**1**).

Figure S11-S22: Spectral data (LC/MS, ^{1}H NMR, ^{13}C NMR, 2D NMR (COSY, HSQC, HMBC, NOESY)) of 5,6-dehydro-desacyl-epipachysamine A (**2**).

Figure S23-S29: Spectral data (LC/MS, ^{1}H NMR, ^{13}C NMR) of desacyl-epipachysamine A (**3**).

Figure S30-S37: Spectral data (LC/MS, ^{1}H NMR, ^{13}C NMR) of epipachysamine B (**4**).

Figure S38-S45: Spectral data (LC/MS, ^{1}H NMR, ^{13}C NMR) of pactermine A (**5**).

Figure S46-S57: Spectral data (LC/MS, ^{1}H NMR, ^{13}C NMR, 2D NMR (COSY, HSQC, HMBC, NOESY)) of *N*3-chloromethyl-desacyl-epipachysamine A (**6**).

Figure S58-S71: Spectral data (LC/MS, ^{1}H NMR, ^{13}C NMR, 2D NMR (COSY, HSQC, HMBC, NOESY)) of 3*α*,4*α*-diapachysanaximine A (**7**).

Figure S72-S78: Spectral data (LC/MS, ^{1}H NMR, ^{13}C NMR) of pachysamine A (**8**).

Figure S79-S89: Spectral data (LC/MS, ^{1}H NMR, ^{13}C NMR, 2D NMR (COSY, HSQC, HMBC, NOESY)) of 3β-dimethylamino-pregnane-20-one (**9**).

Figure S90-S97: Spectral data (LC/MS, ^{1}H NMR, ^{13}C NMR) of pachysandrine D (**10**).

Figure S98-S104: Spectral data (LC/MS, ^{1}H NMR, ^{13}C NMR) of terminaline (**11**).

Figure S105-S111: Spectral data (LC/MS, ^{1}H NMR, ^{13}C NMR) of *N*-methyl-desacyl-epipachysamine A (**12**).

Figure S112-S119: Spectral data (LC/MS, ^{1}H NMR, ^{13}C NMR) of sarcodinine (**13**).

Figure S120-S127: Spectral data (LC/MS, ^{1}H NMR, ^{13}C NMR) of epipachysamine A (**14**).

Figure S128-S136: Spectral data (LC/MS, ^{1}H NMR, ^{13}C NMR, UV and CD) of spiropachysin (**15**).

Figure S137-S149: Spectral data (LC/MS, ^{1}H NMR, ^{13}C NMR, 2D NMR (COSY, HSQC, HMBC, NOESY)) of 4β-hydroxy-hookerianamide N (**16**).

Figure S150-S164: Spectral data (LC/MS, ^{1}H NMR, ^{13}C NMR, 2D NMR (COSY, HSQC, HMBC, NOESY)) of 5α-hydroxy-3α,4α-diapachysanaximine A (**17**).

Figure S165-S177: Spectral data (LC/MS, ^{1}H NMR, ^{13}C NMR, 2D NMR (COSY, HSQC, HMBC, NOESY)) of 2β,3β,4β-diapachysamine K (**18**).

Figure S178-S195: Spectral data (LC/MS, ^{1}H NMR, ^{13}C NMR, ^{15}N NMR, 2D NMR (COSY, HSQC, HMBC, NOESY)) of 3β-dimethylamino-pregnane-20-oxime (**19**) and 3β-dimethylamino-pregn-5,6-ene-20-oxime (**20**).

Table S1. IC_{50}-values (in µg/mL) of the in vitro antiprotozoal (*Plasmodium falciparum, Pf, Trypanosoma brucei rhodesiense, Tbr*) as well as cytotoxic activity (L6 rat skeletal myoblasts) of the CPC fractions from aerial parts of *Pachysandra terminalis*. Data represent arithmetic means of two independent determinations ± their deviation from the mean. SI: Selectivity indices: IC_{50}(Cytotox)/IC_{50}(*Pf* or *Tbr*).

	Pf	***Tbr***	**Cytotox.**	**SI (*Pf*)**	**SI (*Tbr*)**
		CPC fractions			
1	1.3 ± 0.3	8 ± 2	39 ± 3	31	5
2	0.27 ± 0.01	1.5 ± 0.8	5.3 ± 0.4	19	3
3	0.27 ± 0.01	1.6 ± 0.3	12 ± 6	44	7
4	0.20 ± 0.05	0.5 ± 0.3	8 ± 7	38	15
5	0.31 ± 0.04	2.1 ± 0.2	13 ± 3	41	6
6	0.27 ± 0.04	1.9 ± 0.2	15.0 ± 0.4	56	8
7	0.29 ± 0.04	1.8 ± 0.3	15.7 ± 0.2	54	9
8	0.25 ± 0.05	2.0 ± 0.2	14.1 ± 0.3	56	7
9	0.30 ± 0.01	1.5 ± 0.5	13 ± 4	41	8
10	0.173 ± 0.001	0.75 ± 0.06	12 ± 3	69	16
11	0.28 ± 0.05	1.32 ± 0.6	15.1 ± 0.5	54	11
12	0.4 ± 0.1	2.1 ± 0.2	24 ± 4	56	11
13	0.93 ± 0.07	6.2 ± 0.9	47 ± 1	51	8
14	0.41 ± 0.05	6.4 ± 0.8	20 ± 3	49	3
15	0.35 ± 0.02	1.9 ± 0.2	37 ± 10	105	19
16	0.53 ± 0.01	2.1 ± 0.2	20 ± 3	37	9
17	0.26 ± 0.04	1.0 ± 0.4	22.0 ± 0.7	84	21
18	0.34 ± 0.02	1.6 ± 0.2	17.2 ± 0.8	51	11
		Positive controls			
Chloroquine	0.002 ± 0.000				
Melarsoprol		0.007 ± 0.002			
Podophyllotoxin			0.009 ± 0.001		

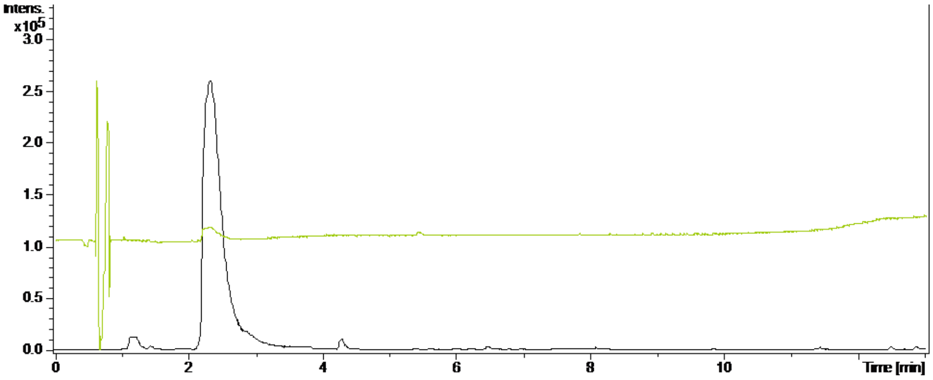

Figure S1. UHPLC/+ESI-QqTOF-MS/MS chromatogram of 9-(*N*,*N*-dimethyl)-5-megastigmen-1-one (**1**). Base peak chromatogram 200.0000-1000.0000 +All MS (black); UV chromatogram 200-400 nm (green).

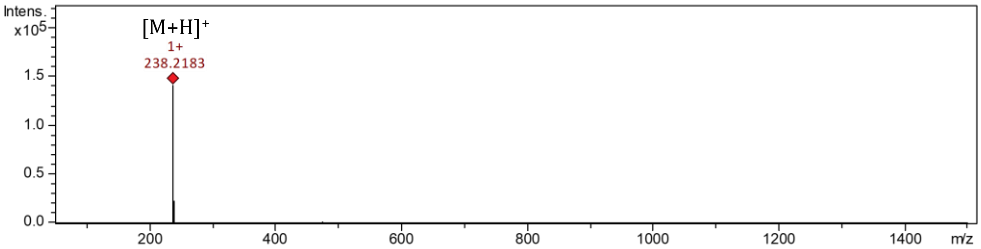

Figure S2. +ESI-QqTOF MS spectrum of 9-(*N*,*N*-dimethyl)-5-megastigmen-1-one (**1**); *m*/*z* 238.2183 [M+H]⁺.

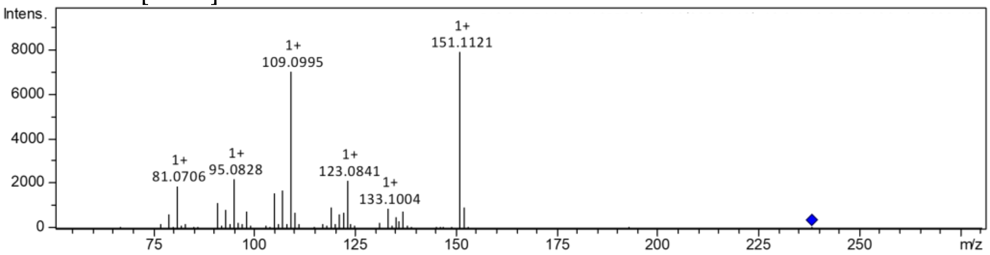

Figure S3. +ESI-QqTOF MS/MS spectrum of 9-(*N*,*N*-dimethyl)-5-megastigmen-1-one (**1**).

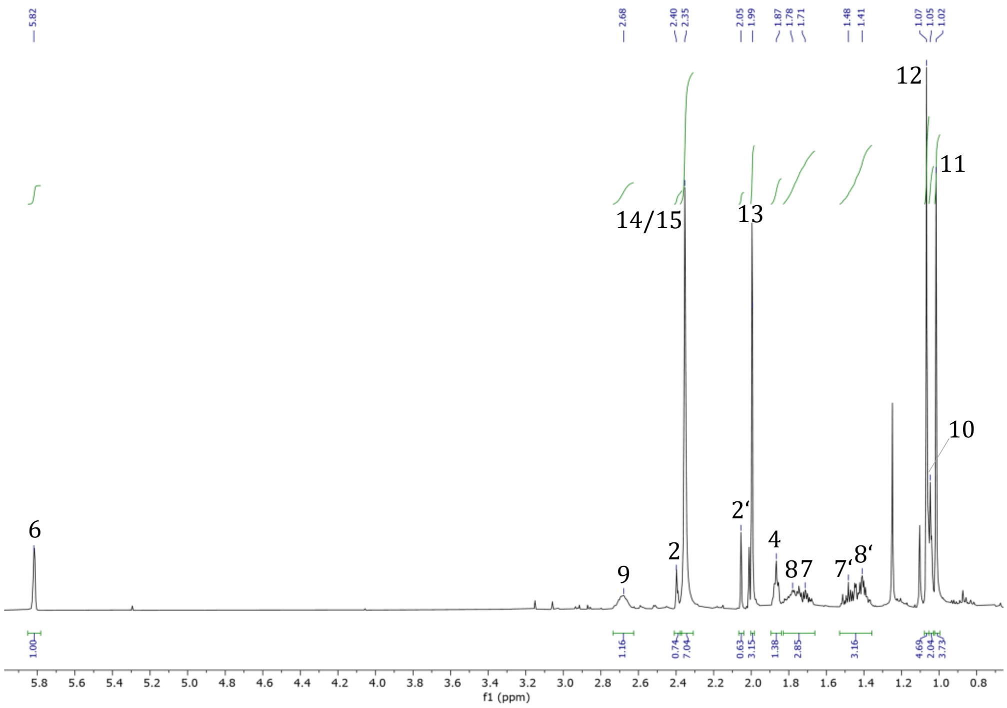

Figure S4. ^{1}H NMR spectrum of 9-(*N*,*N*-dimethyl)-5-megastigmen-1-one (**1**) ($CDCl_3$, 600 MHz).

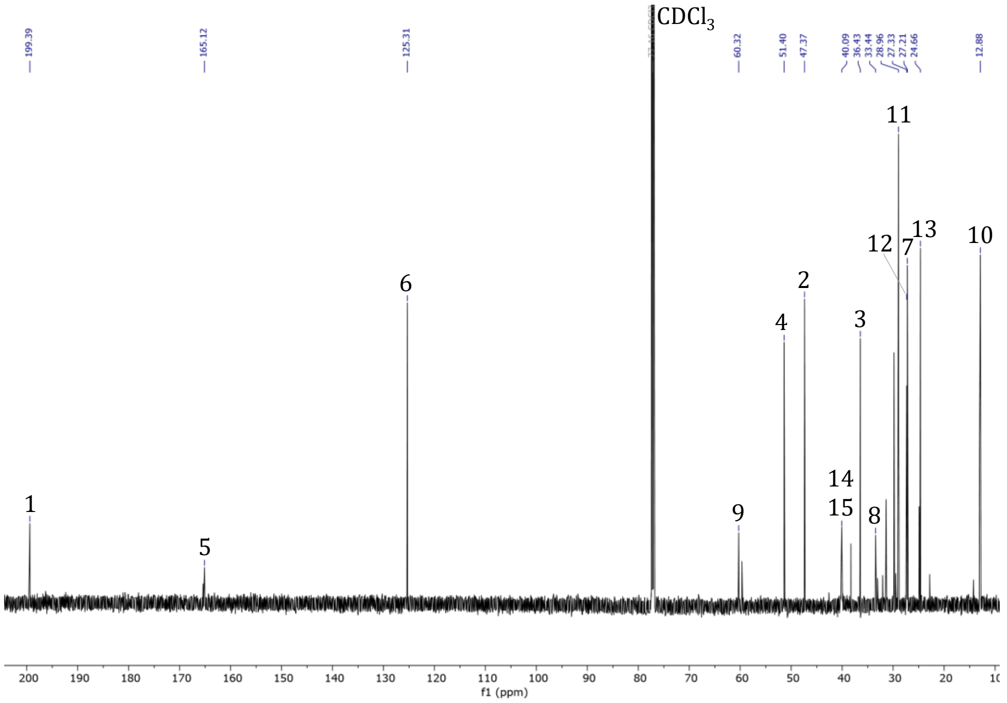

Figure S5. ^{13}C NMR spectrum of 9-(*N*,*N*-dimethyl)-5-megastigmen-1-one (**1**) ($CDCl_3$, 151 MHz).

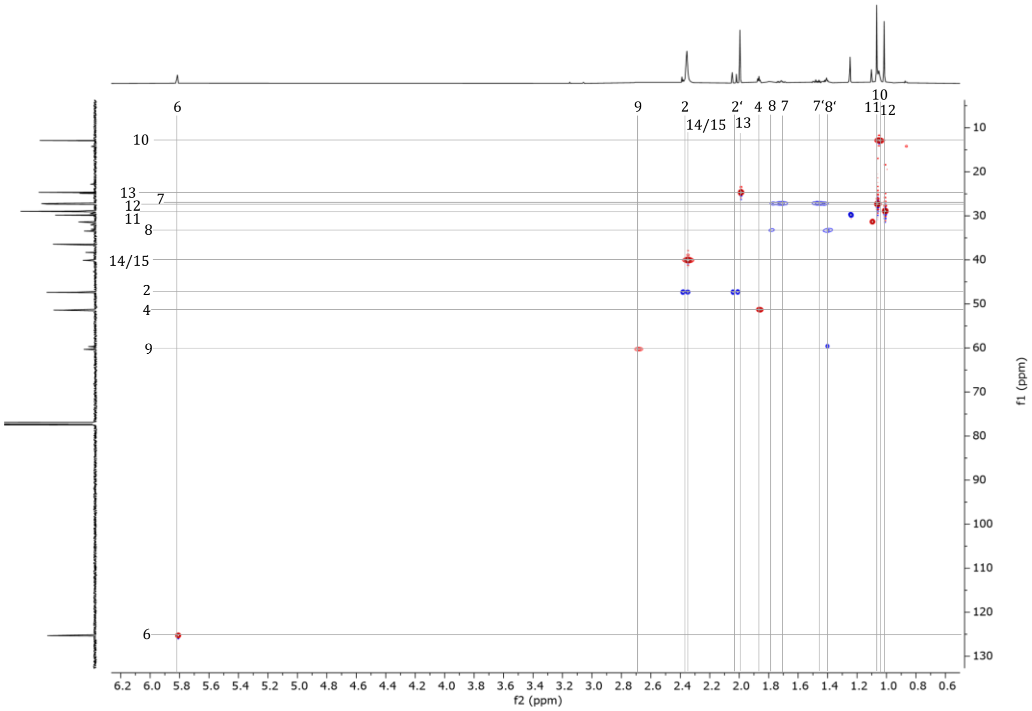

Figure S6. $^{1}H/^{13}C$ HSQC spectrum of 9-(*N*,*N*-dimethyl)-5-megastigmen-1-one (**1**) ($CDCl_3$, 600/151 MHz).

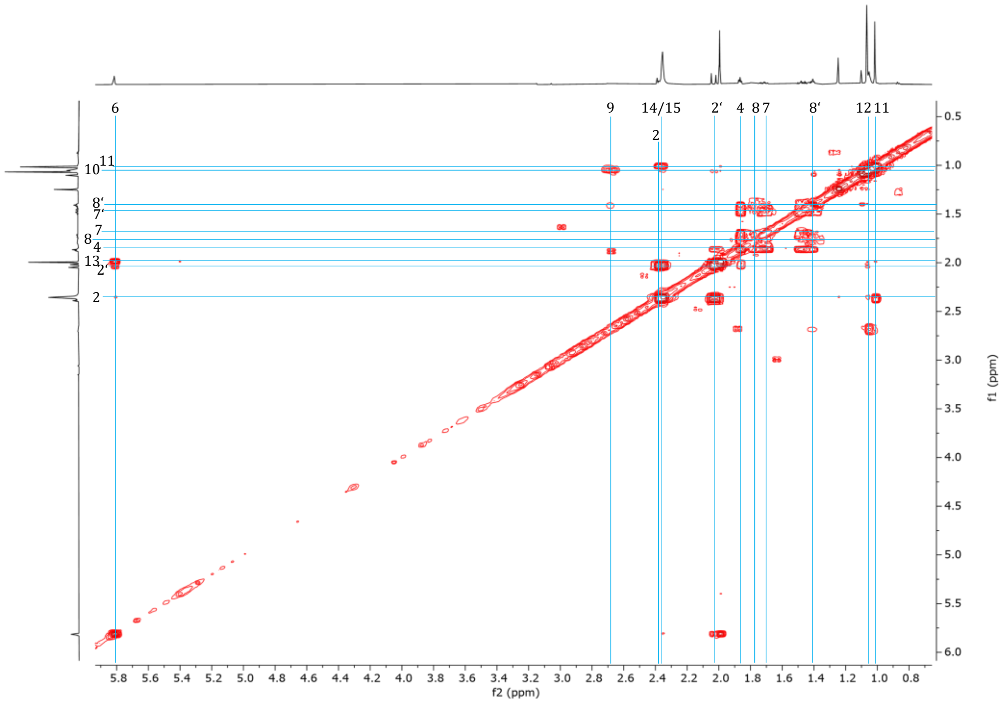

Figure S7. $^{1}H/^{1}H$ COSY spectrum of 9-(*N*,*N*-dimethyl)-5-megastigmen-1-one (**1**) ($CDCl_3$, 600 MHz).

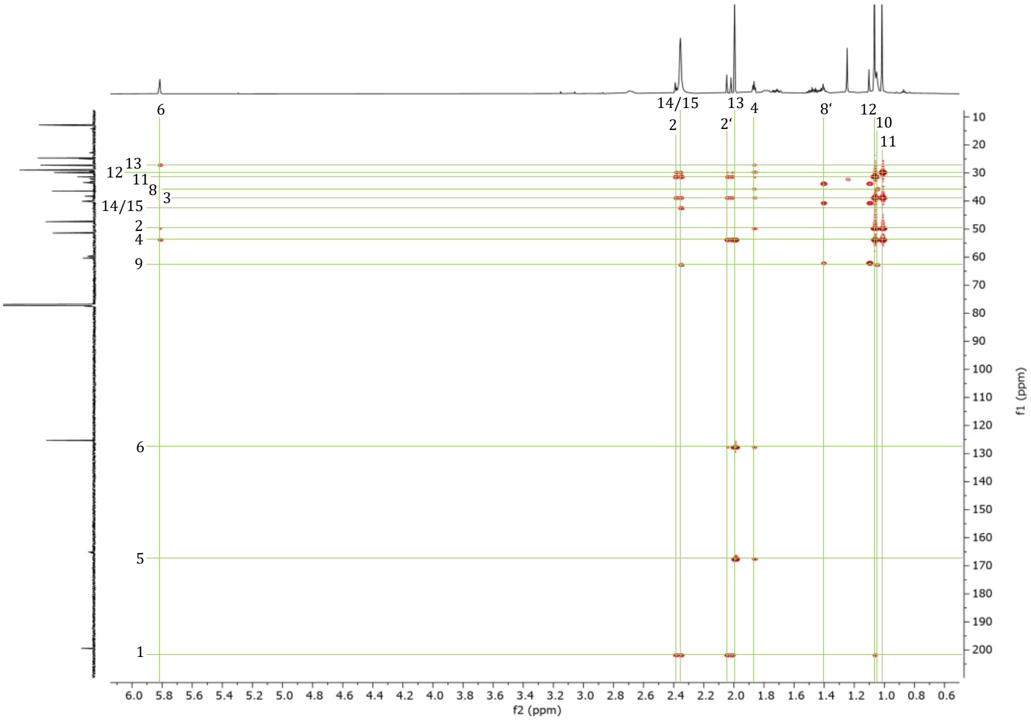

Figure S8. $^{1}H/^{13}C$ HMBC spectrum of 9-(*N*,*N*-dimethyl)-5-megastigmen-1-one (**1**) ($CDCl_3$, 600/151 MHz).

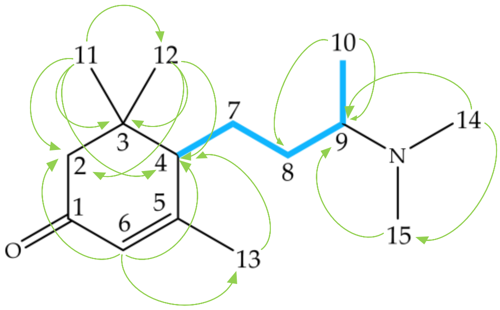

Figure S9. Key COSY (blue lines) and HMBC (green arrows) correlations of 9-(*N*,*N*-dimethyl)-5-megastigmen-1-one (**1**).

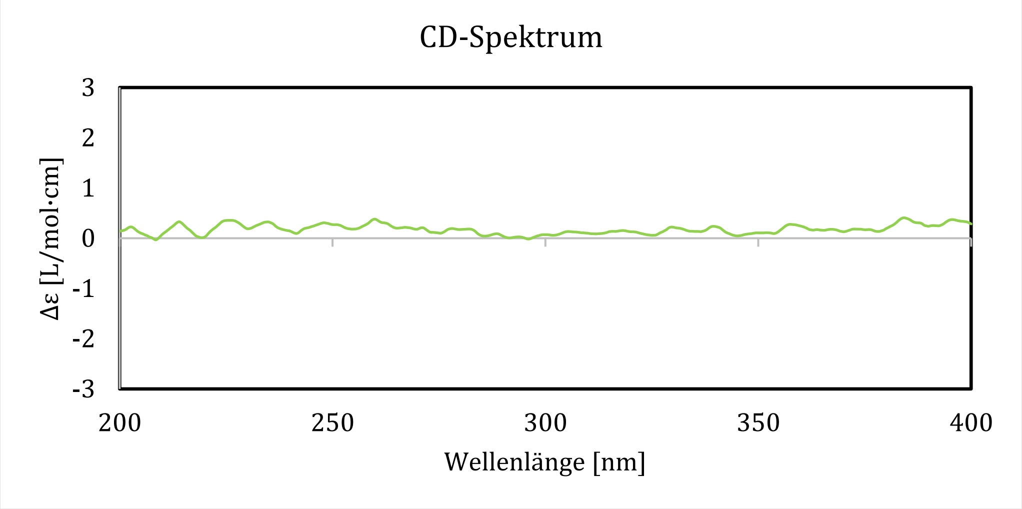

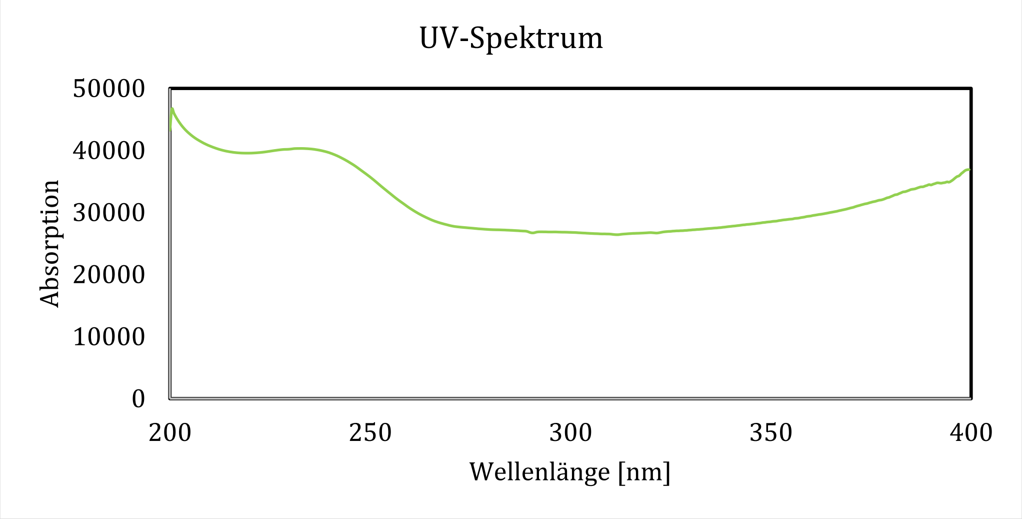

Figure S10. CD and UV spectrum of 9-(*N*,*N*-dimethyl)-5-megastigmen-1-one (**1**) in MeOH (0.1 mg/mL).

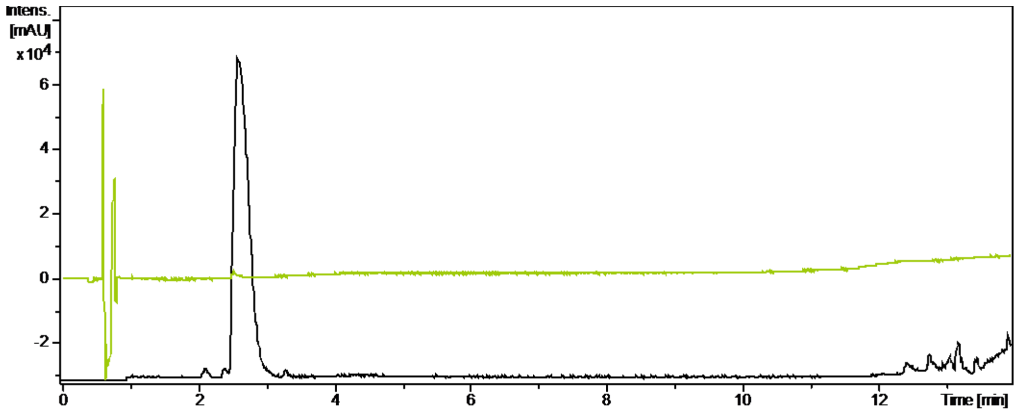

Figure S11. UHPLC/+ESI-QqTOF-MS/MS chromatogram of 5,6-dehydro-desacyl-epi-pachysamine A (**2**). Base peak chromatogram 200.0000-1000.0000 +All MS (black); UV chromatogram 200-400 nm (green).

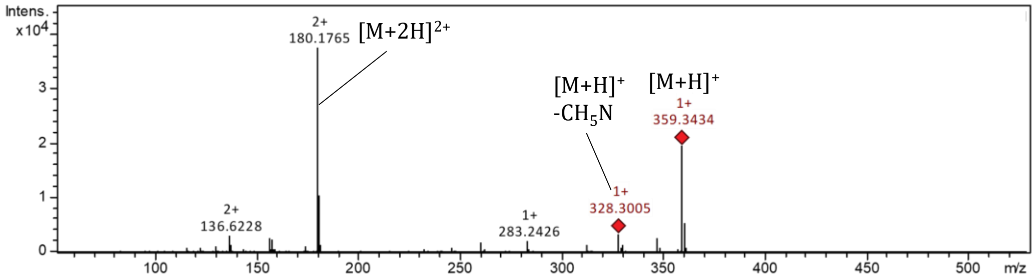

Figure S12. +ESI-QqTOF MS spectrum of 5,6-dehydro-desacyl-epipachysamine A (**2**); *m/z* 359.3434 $[M+H]^+$, *m/z* 180.1765 $[M+2H]^{2+}$.

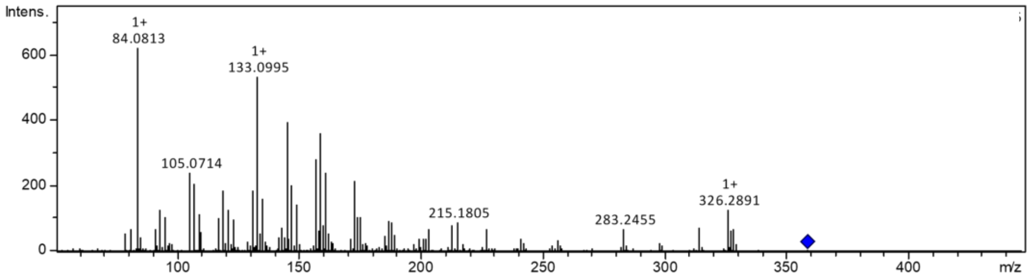

Figure S13. +ESI-QqTOF MS/MS spectrum of 5,6-dehydro-desacyl-epipachysamine A (**2**).

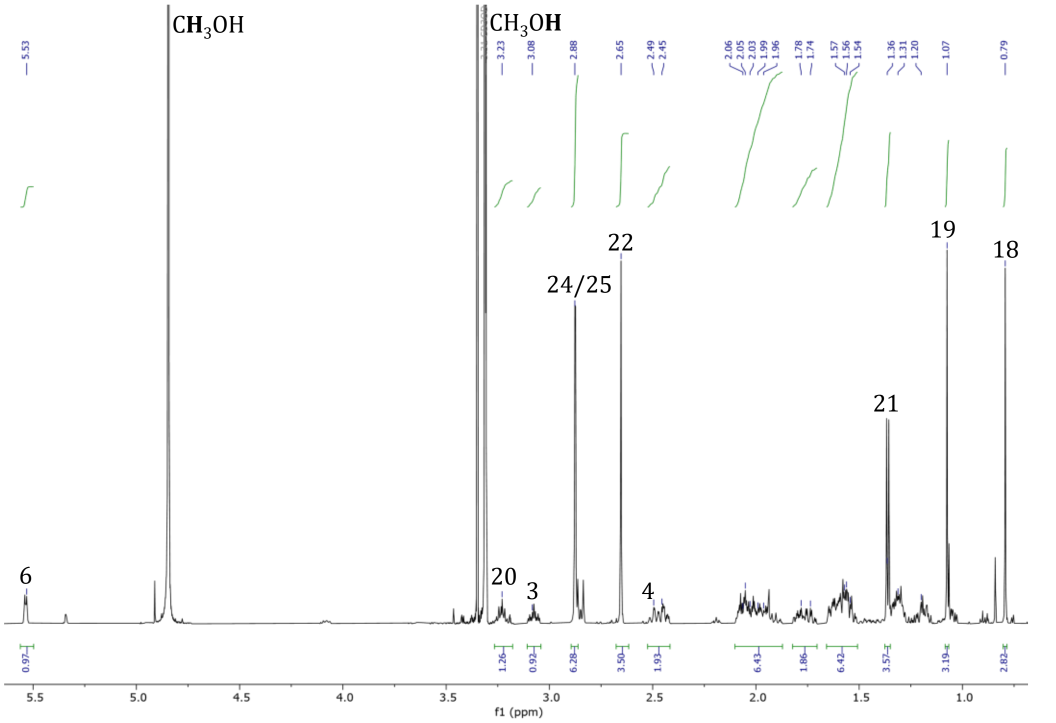

Figure S14. ^1H NMR spectrum of 5,6-dehydro-desacyl-epipachysamine A (**2**) (CD_3OD, 600 MHz). The assignment of the signals between 2.55 and 0.75 ppm can be found in the enlarged Figure S15.

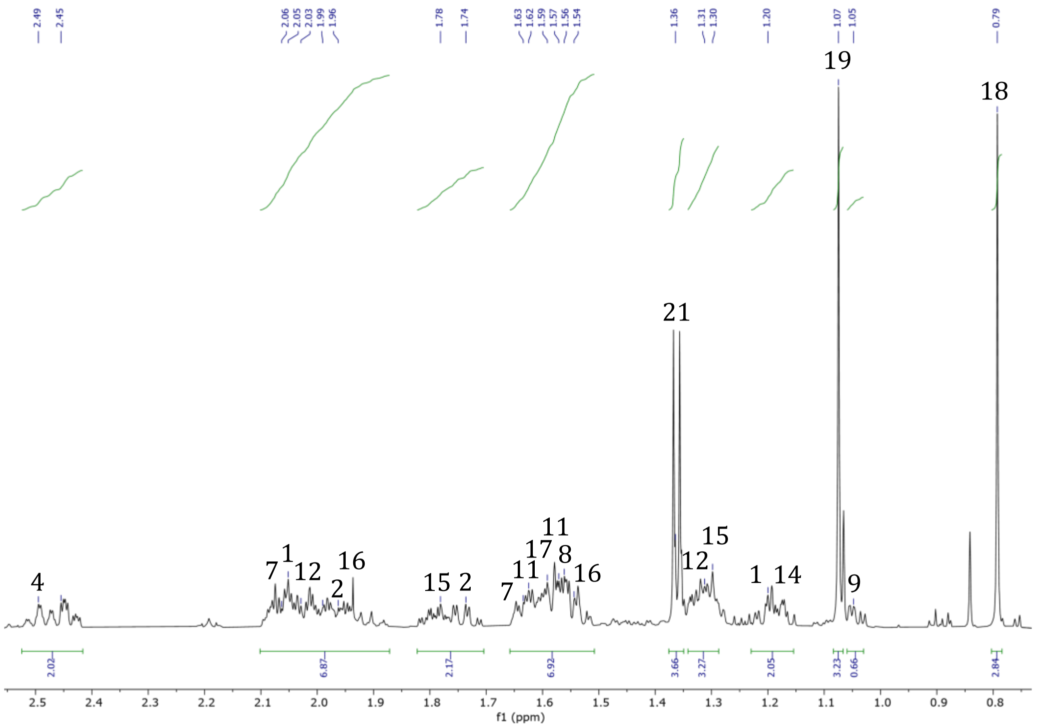

Figure S15. Detail of the ^{1}H NMR spectrum of 5,6-dehydro-desacyl-epipachysamine A (**2**) (CD_3OD, 600 MHz).

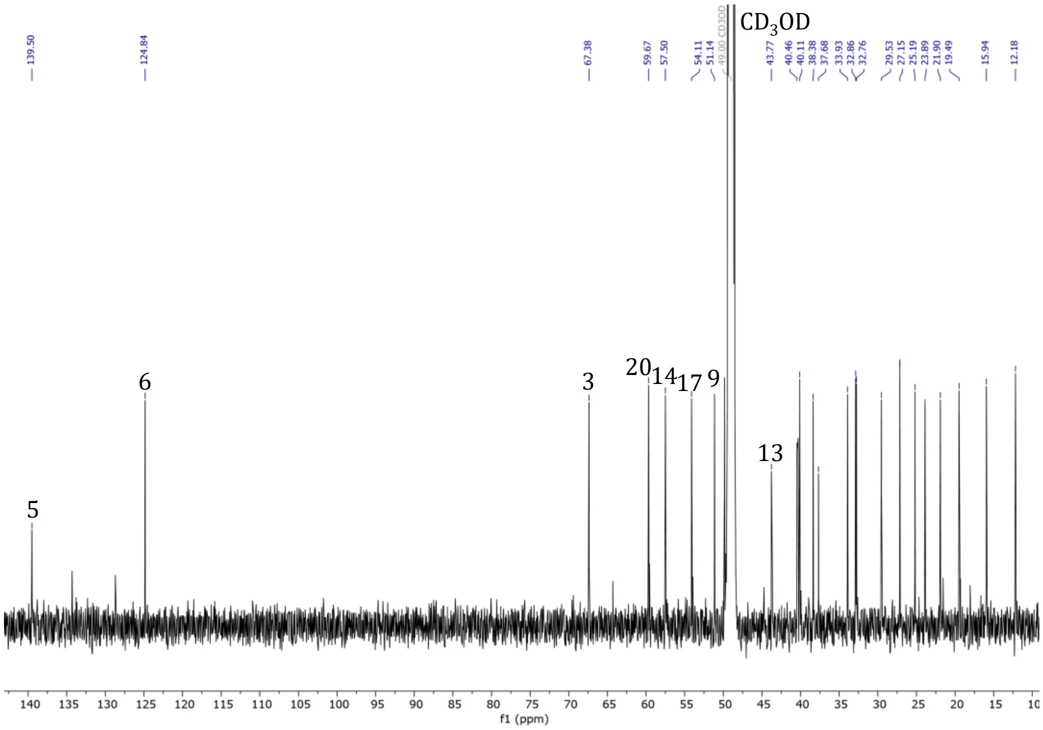

Figure S16. ^{13}C NMR spectrum of 5,6-dehydro-desacyl-epipachysamine A (**2**) (CD_3OD, 151 MHz). The assignment of the signals between 46 and 11 ppm can be found in the enlarged Figure S17.

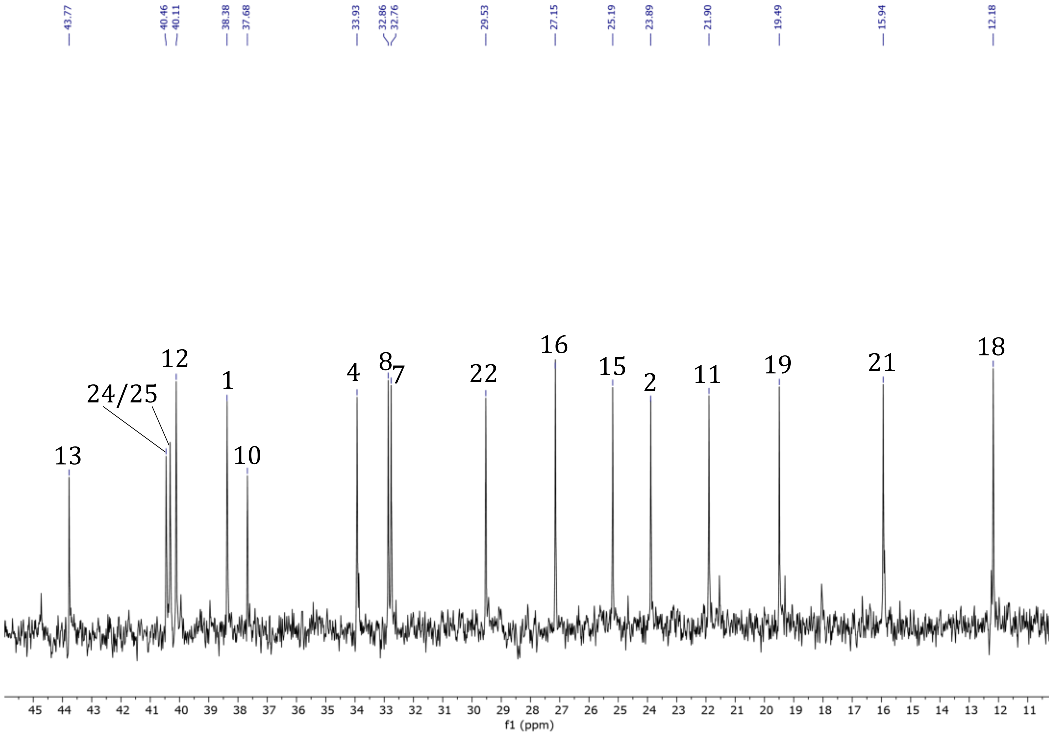

Figure S17. Detail of the ^{13}C NMR spectrum of 5,6-dehydro-desacyl-epipachysamine A (**2**) (CD_3OD, 151 MHz).

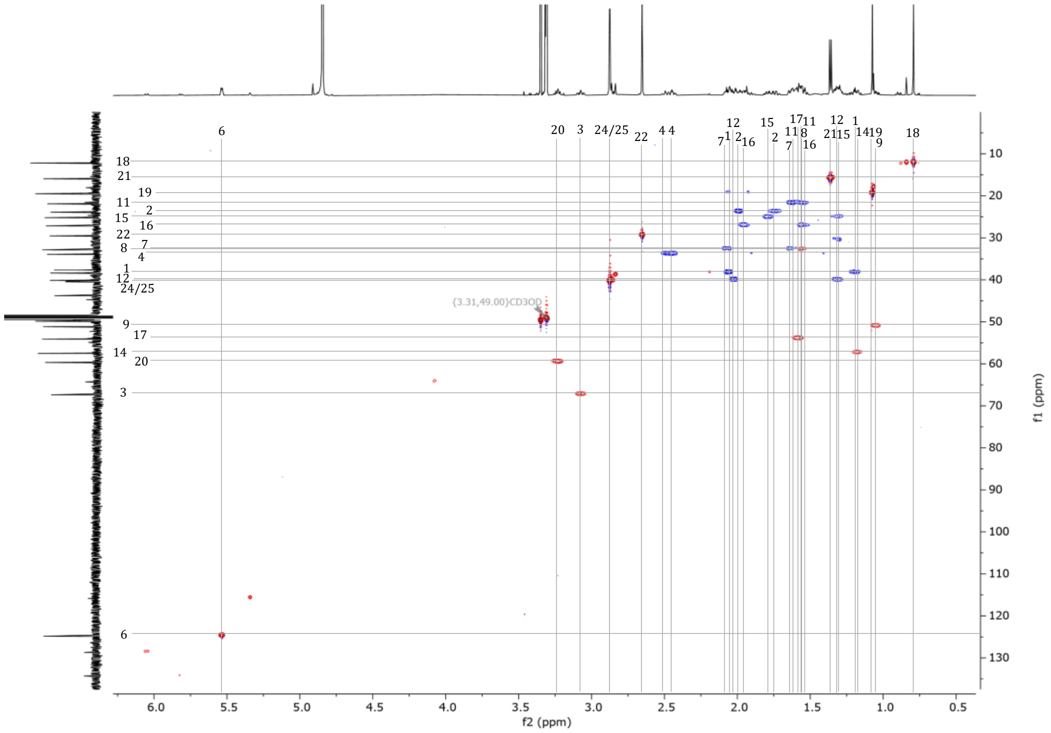

Figure S18. ^{1}H/^{13}C HSQC spectrum of 5,6-dehydro-desacyl-epipachysamine A (**2**) (CD_3OD, 600/151 MHz).

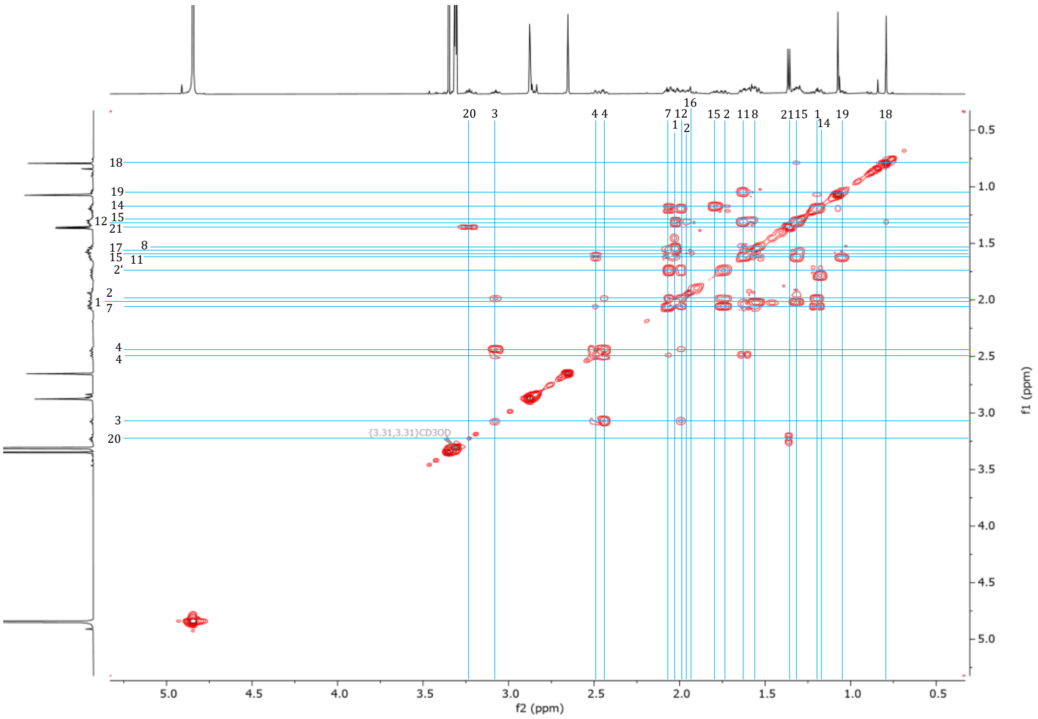

Figure S19. $^1H/^1H$ COSY spectrum of 5,6-dehydro-desacyl-epipachysamine A (**2**) (CD_3OD, 600 MHz).

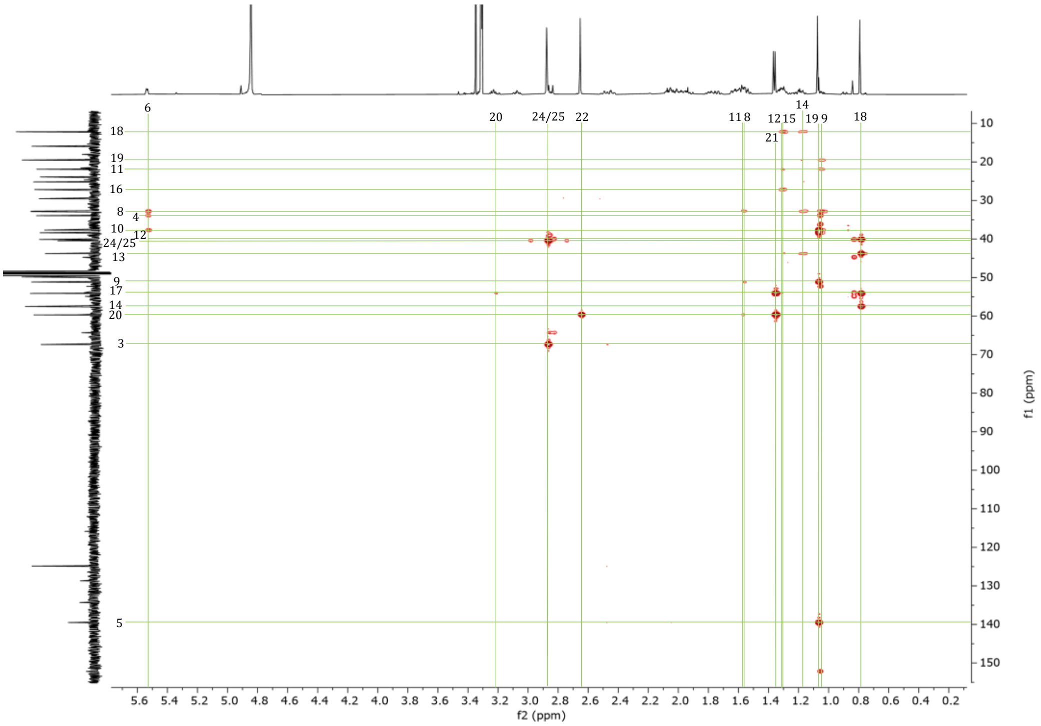

Figure S20. $^1H/^{13}C$ HMBC spectrum of 5,6-dehydro-desacyl-epipachysamine A (**2**) (CD_3OD, 600/151 MHz).

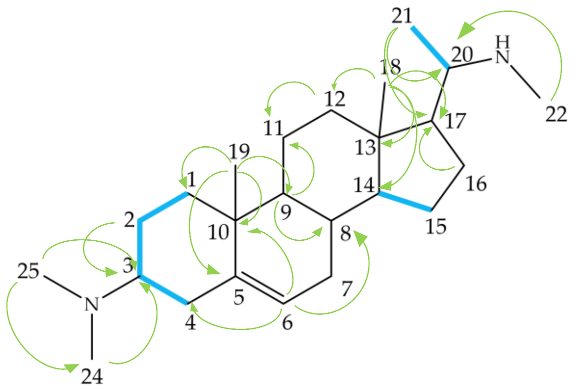

Figure S21. Key COSY (blue lines) and HMBC (green arrows) correlations of 5,6-dehydro-desacyl-epipachysamine A (**2**).

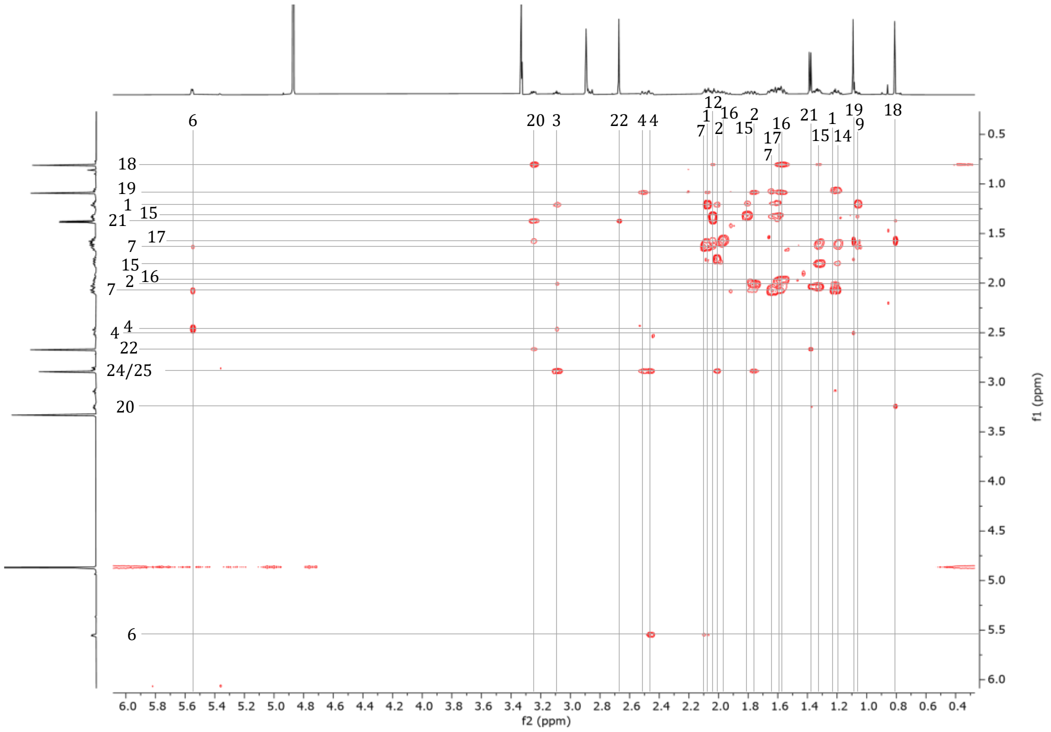

Figure S22. ¹H/¹H NOESY spectrum of 5,6-dehydro-desacyl-epipachysamine A (**2**) (CD_3OD, 600 MHz).

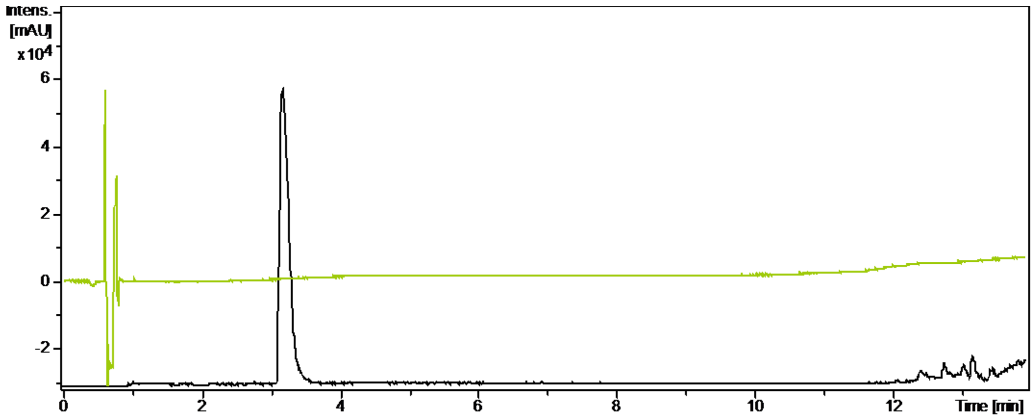

Figure S23. UHPLC/+ESI-QqTOF-MS/MS chromatogram of desacyl-epipachysamine A (**3**). Base peak chromatogram 200.0000-1000.0000 +All MS (black); UV chromatogram 200-400 nm (green).

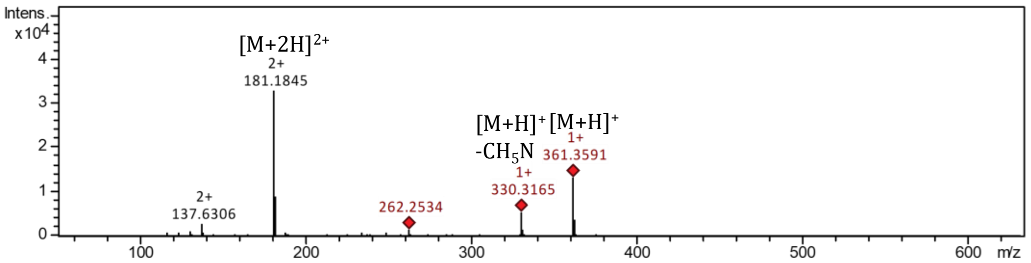

Figure S24. +ESI-QqTOF MS spectrum of desacyl-epipachysamine A (**3**); *m*/*z* 361.3591 $[M+H]^+$, *m*/*z* 181.1845 $[M+2H]^{2+}$.

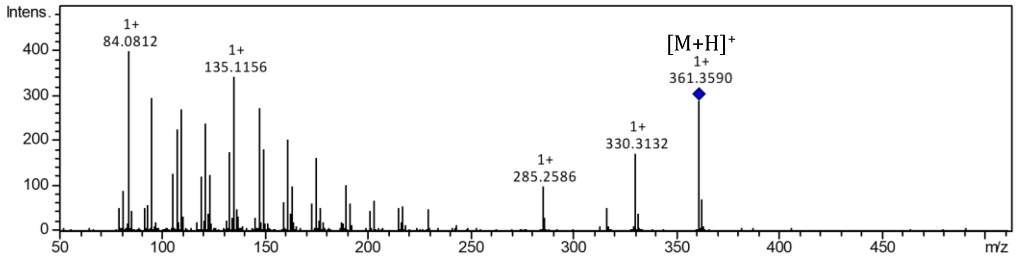

Figure S25. +ESI-QqTOF MS/MS spectrum of desacyl-epipachysamine A (**3**).

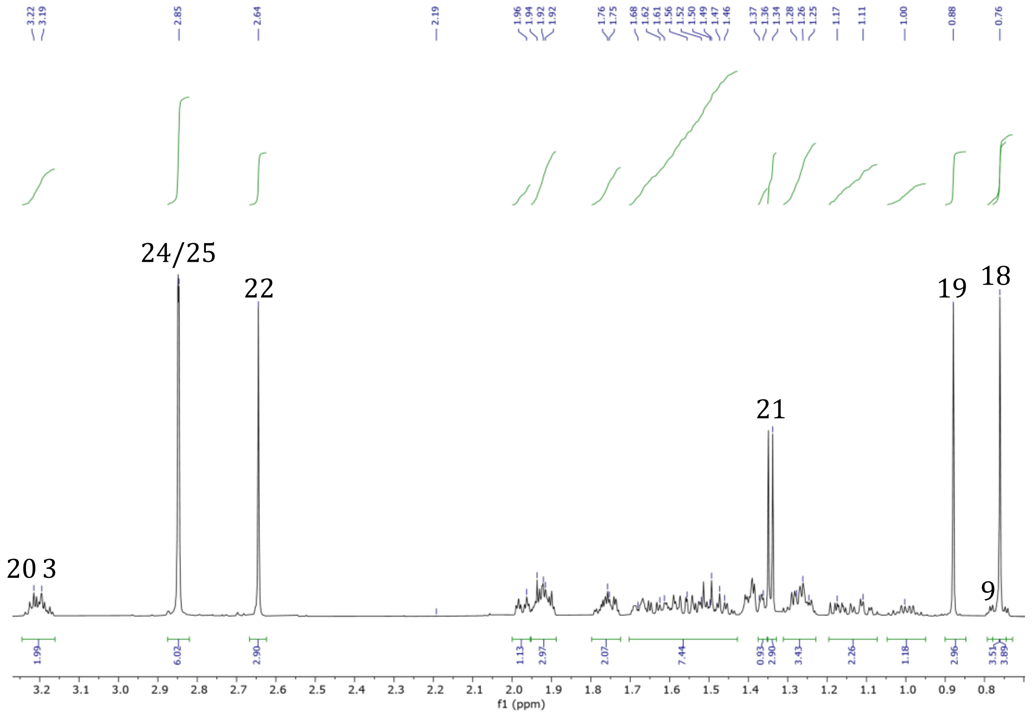

Figure S26. ^1H NMR spectrum of desacyl-epipachysamine A (**3**) (CD_3OD, 600 MHz). The assignment of the signals between 2.00 and 0.90 ppm can be found in the enlarged Figure S27.

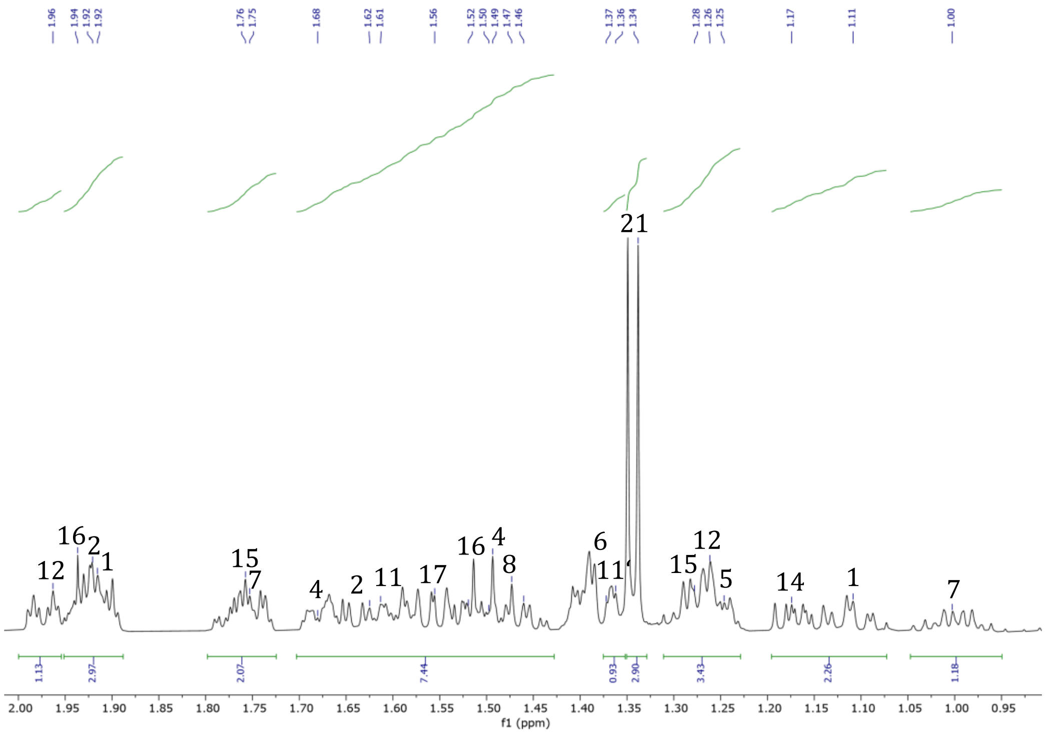

Figure S27. Detail of the ^{1}H NMR spectrum of desacyl-epipachysamine A (**3**) (CD_3OD, 600 MHz).

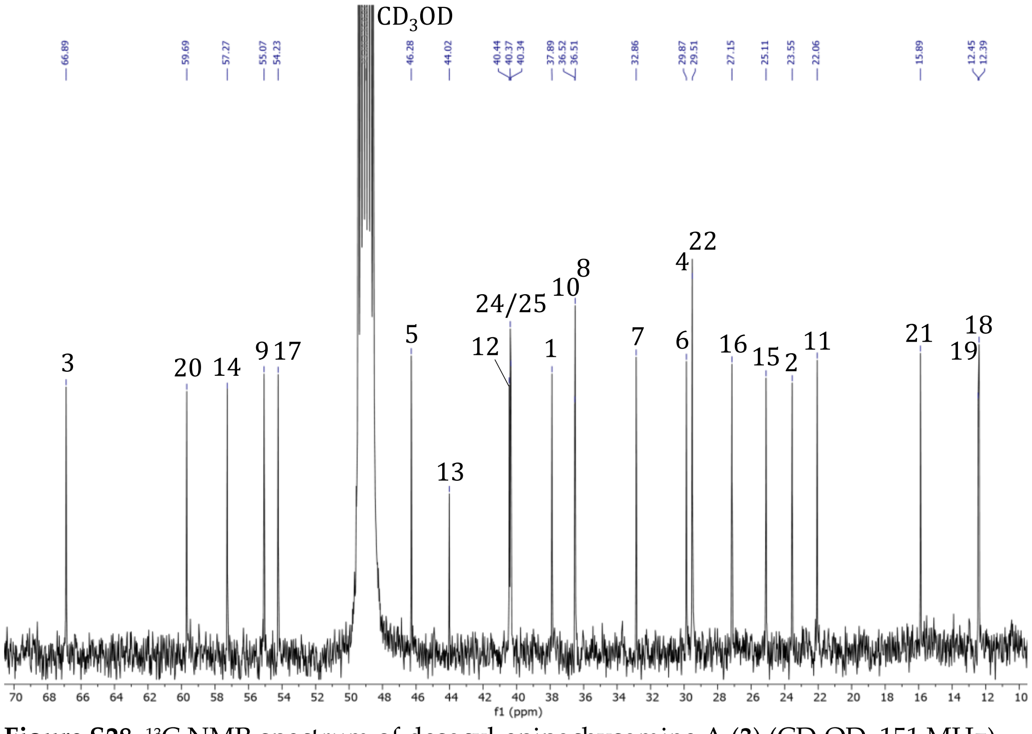

Figure S28. ^{13}C NMR spectrum of desacyl-epipachysamine A (**3**) (CD_3OD, 151 MHz).

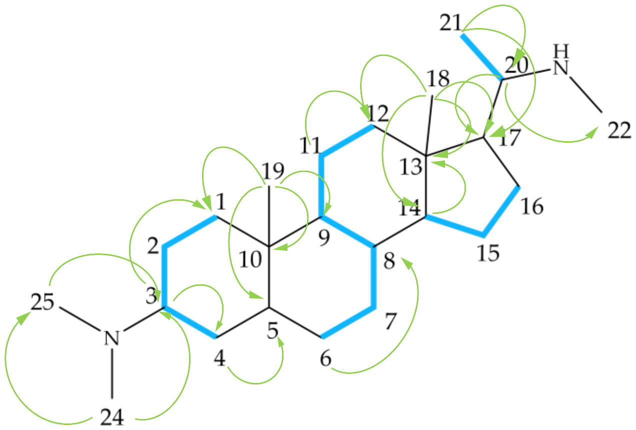

Figure S29. Key COSY (blue lines) and HMBC (green arrows) correlations of desacyl-epi-pachysamine A (**3**).

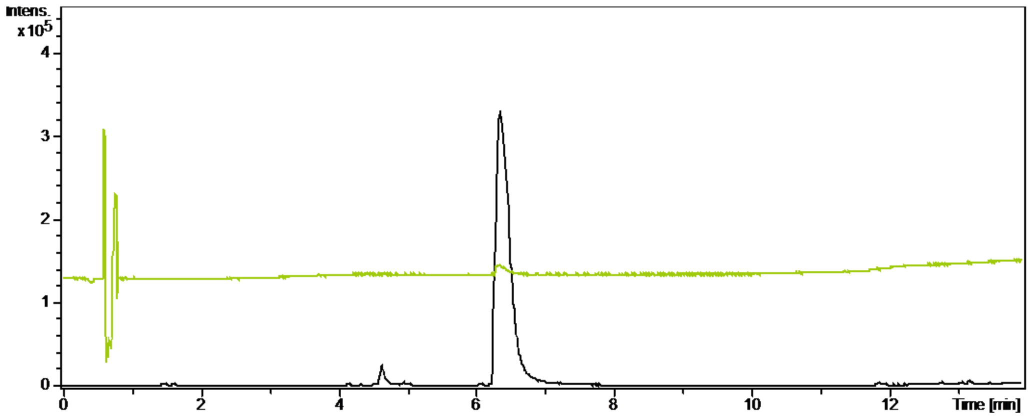

Figure S30. UHPLC/+ESI-QqTOF-MS/MS chromatogram of epipachysamine B (**4**). Base peak chromatogram 200.0000-1000.0000 +All MS (black); UV chromatogram 200-400 nm (green).

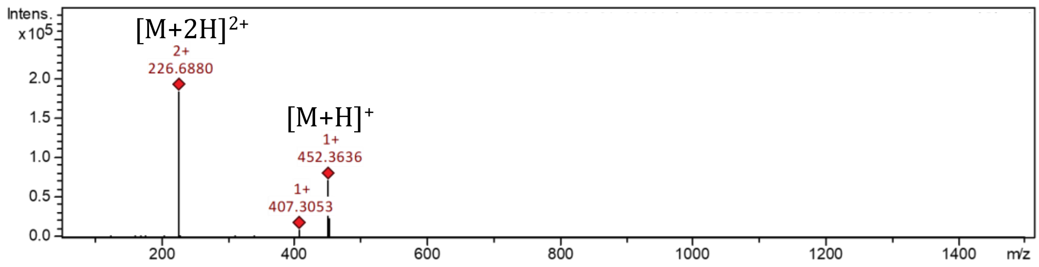

Figure S31. +ESI-QqTOF MS spectrum of epipachysamine B (**4**); *m/z* 452.3636 $[M+H]^+$, *m/z* 226.6880 $[M+2H]^{2+}$.

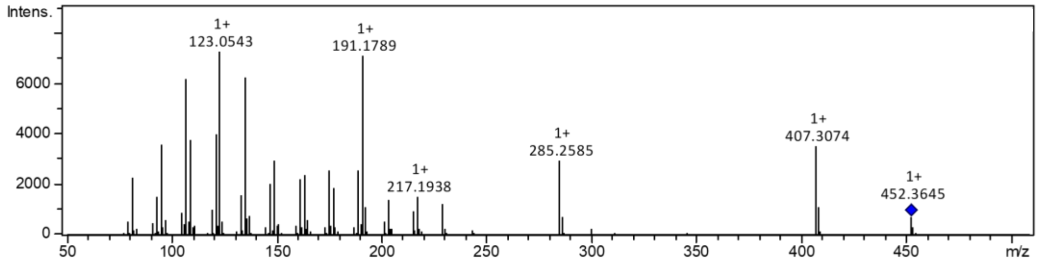

Figure S32. +ESI-QqTOF MS/MS spectrum of epipachysamine B (**4**).

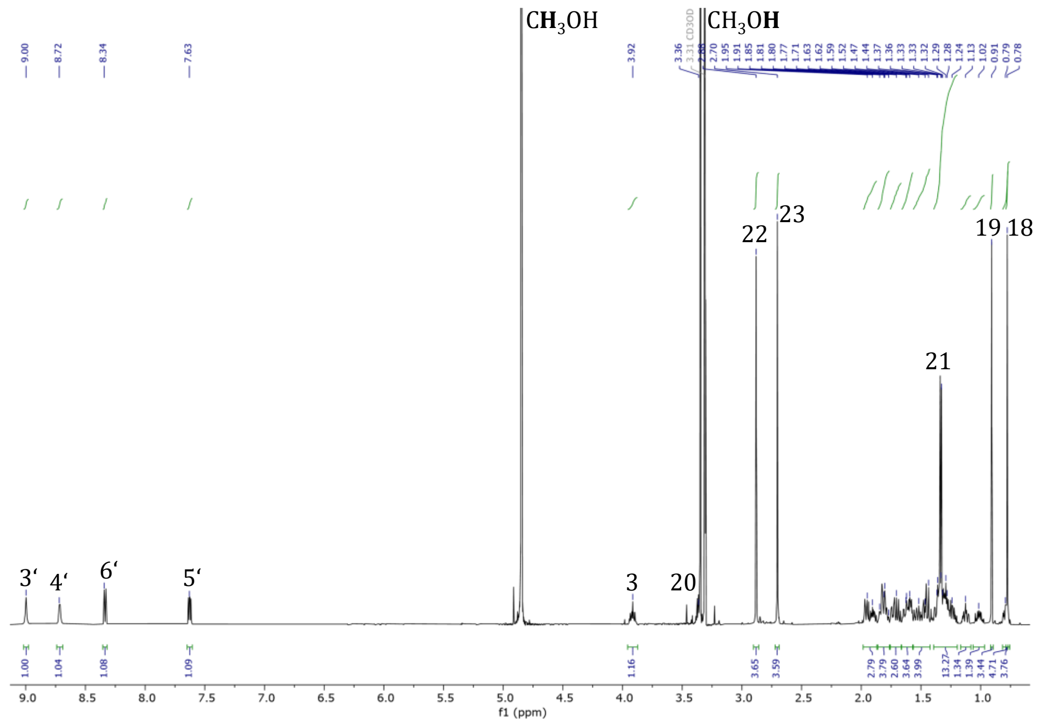

Figure S33. ^{1}H NMR spectrum of epipachysamine B (**4**) (CD_3OD, 600 MHz). The assignment of the signals between 2.00 and 0.70 ppm can be found in the enlarged Figure S34.

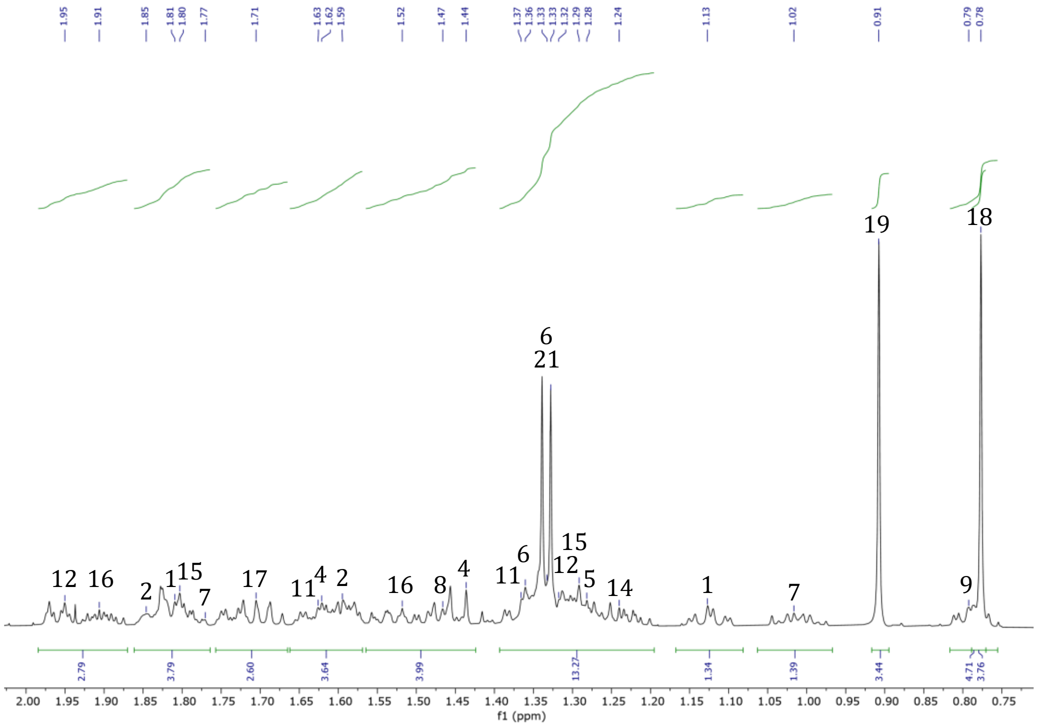

Figure S34. Detail of the ^{1}H NMR spectrum of epipachysamine B (**4**) (CD_3OD, 600 MHz).

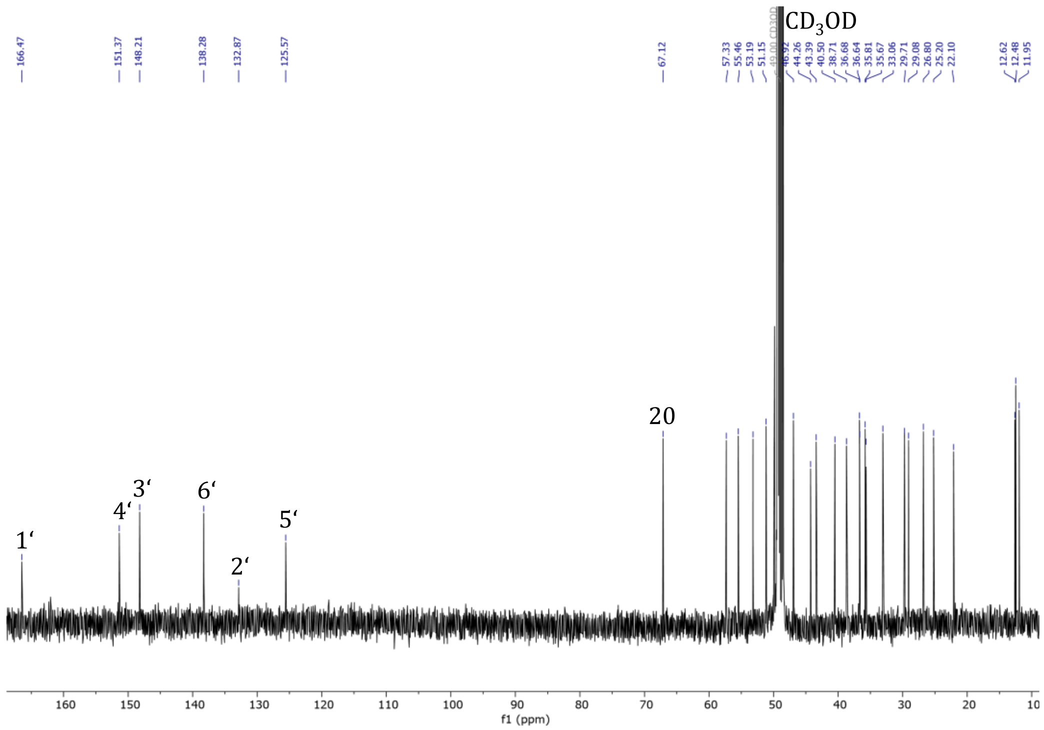

Figure S35. ^{13}C NMR spectrum of epipachysamine B (**4**) (CD_3OD, 151 MHz). The assignment of the signals between 69 and 11 ppm can be found in the enlarged Figure S36.

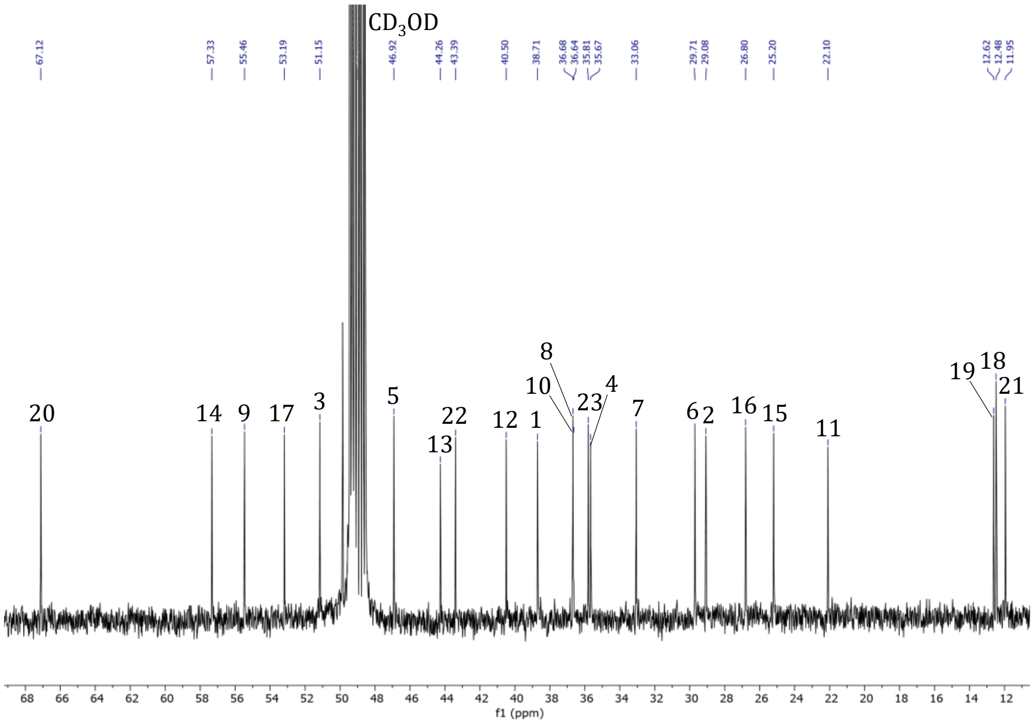

Figure S36. Detail of the ^{13}C NMR spectrum of epipachysamine B (**4**) (CD_3OD, 151 MHz).

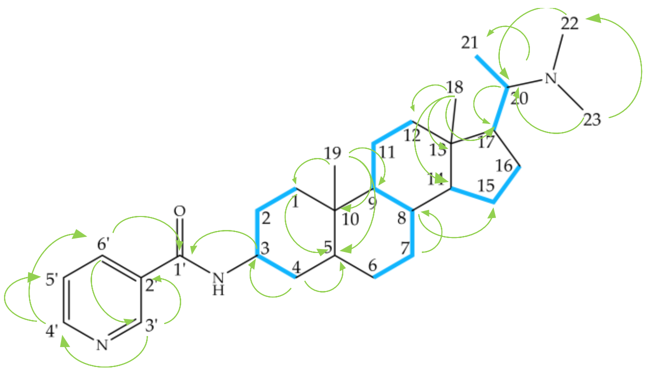

Figure S37. Key COSY (blue lines) and HMBC (green arrows) correlations of epipachysamine B (**4**).

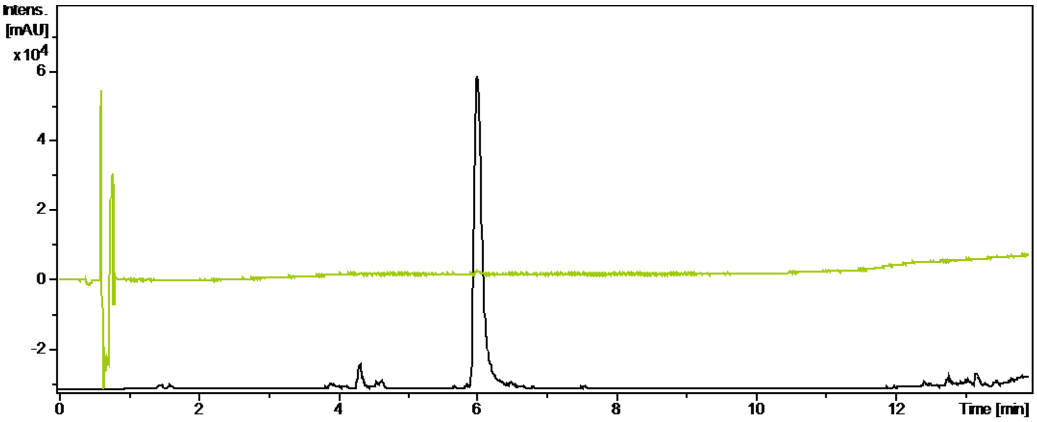

Figure S38. UHPLC/+ESI-QqTOF-MS/MS chromatogram of pactermine A (**5**). Base peak chromatogram 200.0000-1000.0000 +All MS (black); UV chromatogram 200-400 nm (green).

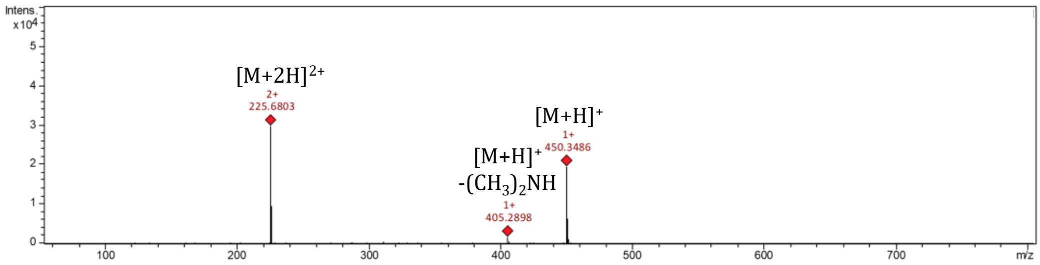

Figure S39. +ESI-QqTOF MS spectrum of pactermine A (**5**); *m/z* 450.3486 $[M+H]^+$, *m/z* 225.6803 $[M+2H]^{2+}$.

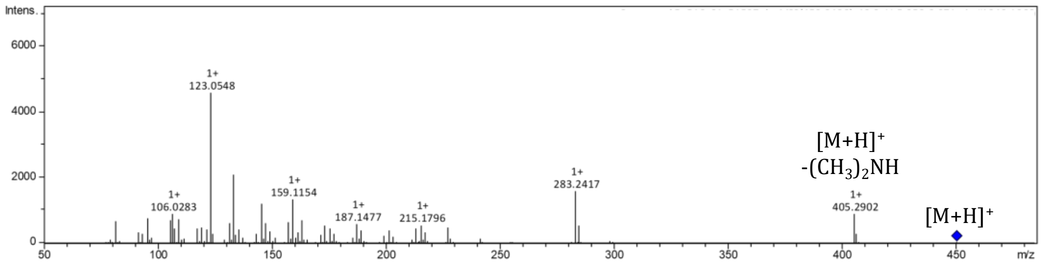

Figure S40. +ESI-QqTOF MS/MS spectrum of pactermine A (**5**).

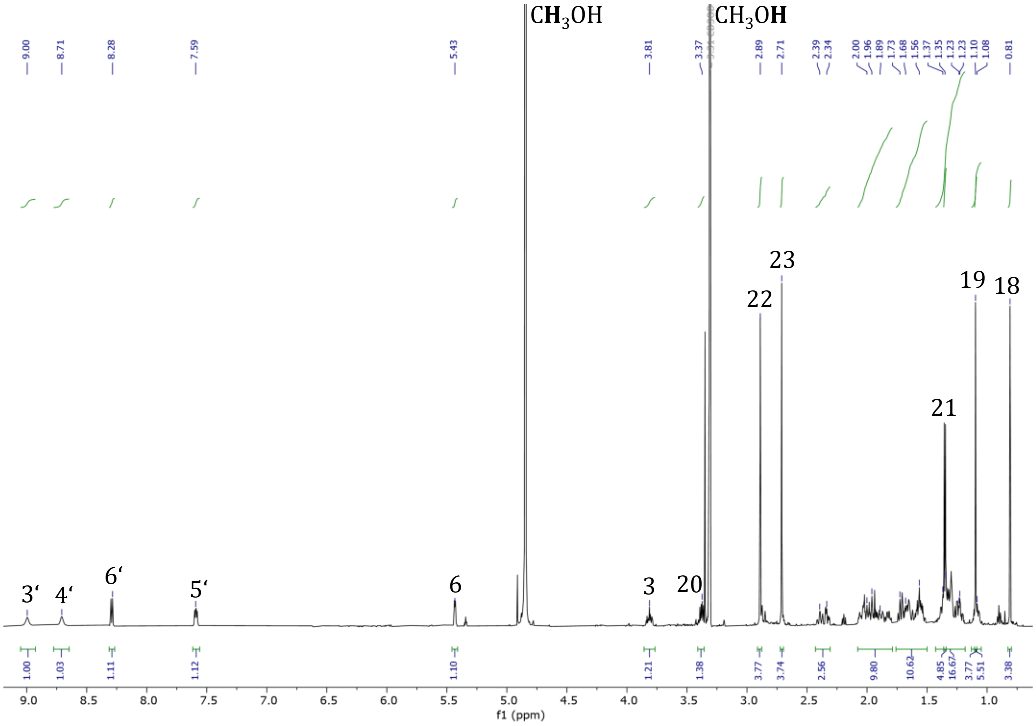

Figure S41. ^{1}H NMR spectrum of pactermine A (**5**) (CD_3OD, 600 MHz). The assignment of the signals between 2.50 and 0.70 ppm can be found in the enlarged Figure S42.

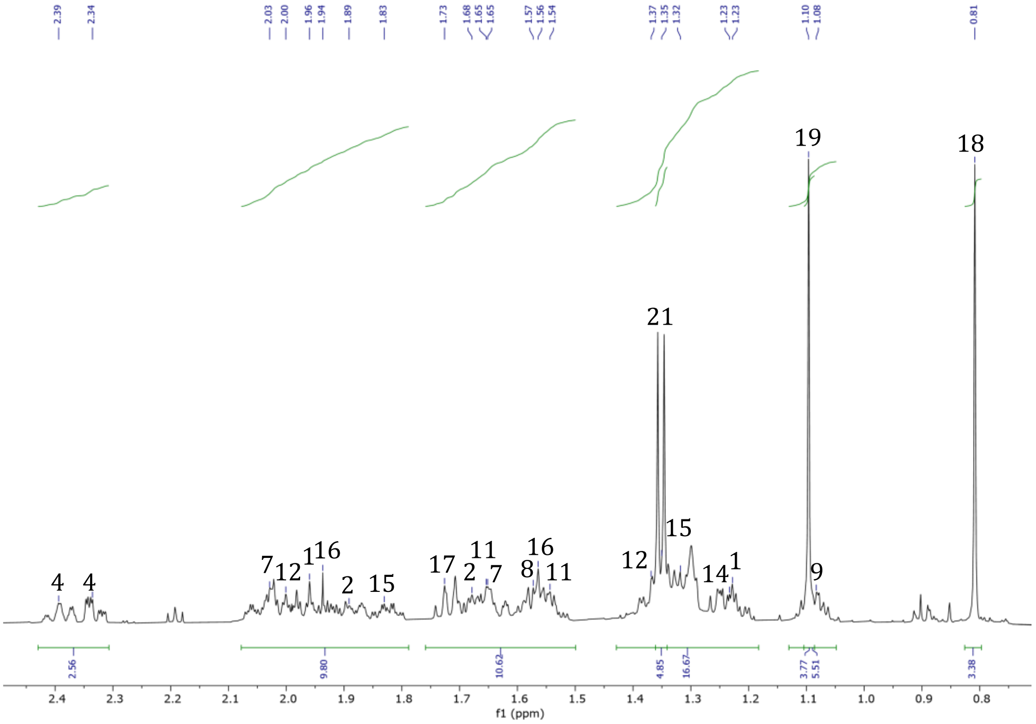

Figure S42. Detail of the ^{1}H NMR spectrum of pactermine A (**5**) (CD_3OD, 600 MHz).

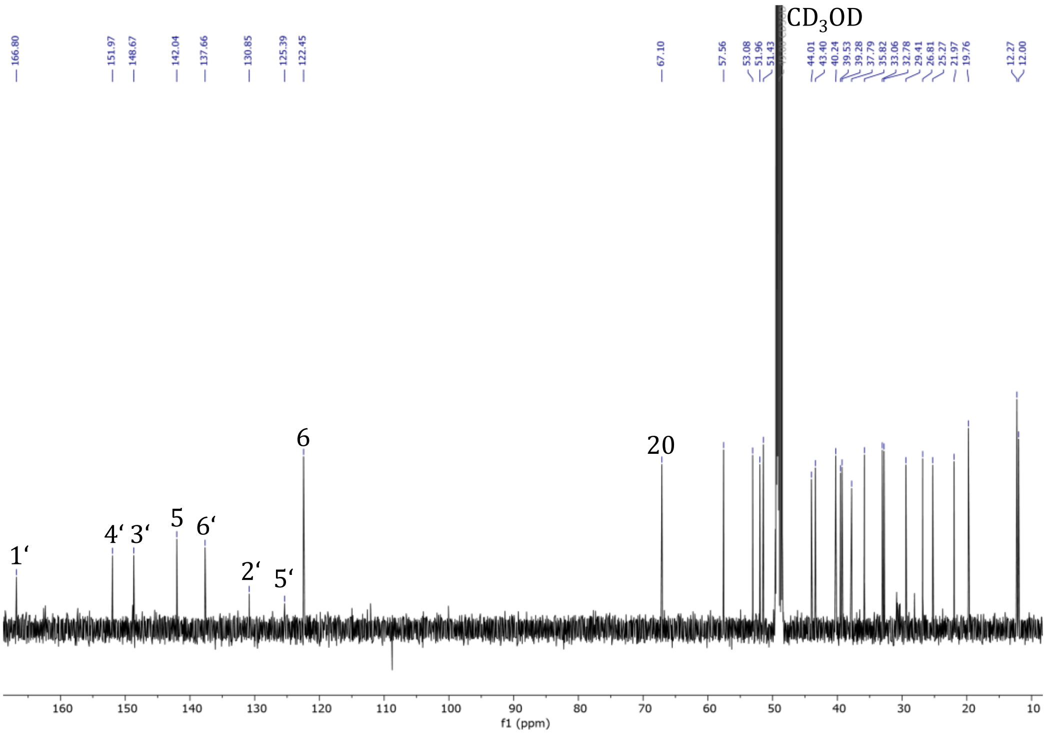

Figure S43. ^{13}C NMR spectrum of pactermine A (**5**) (CD_3OD, 151 MHz). The assignment of the signals between 67 and 11 ppm can be found in the enlarged Figure S44.

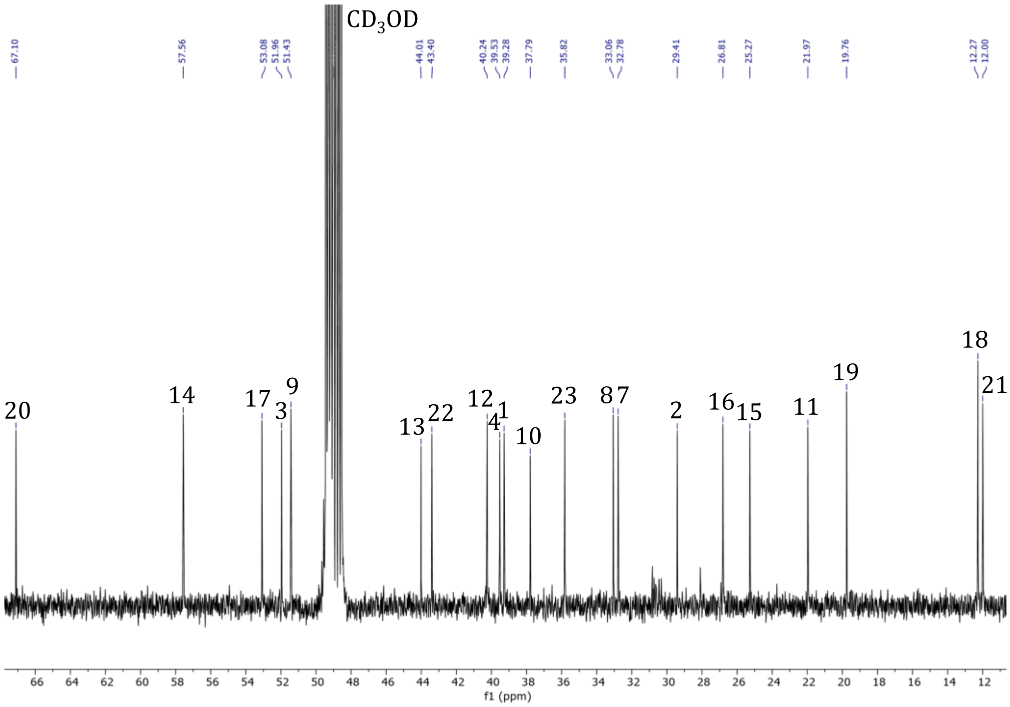

Figure S44. Detail of the ^{13}C NMR spectrum of pactermine A (**5**) (CD_3OD, 151 MHz).

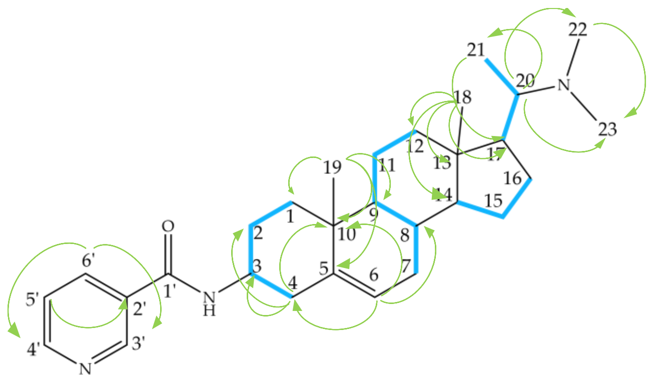

Figure S45. Key COSY (blue lines) and HMBC (green arrows) correlations of pactermine A (**5**).

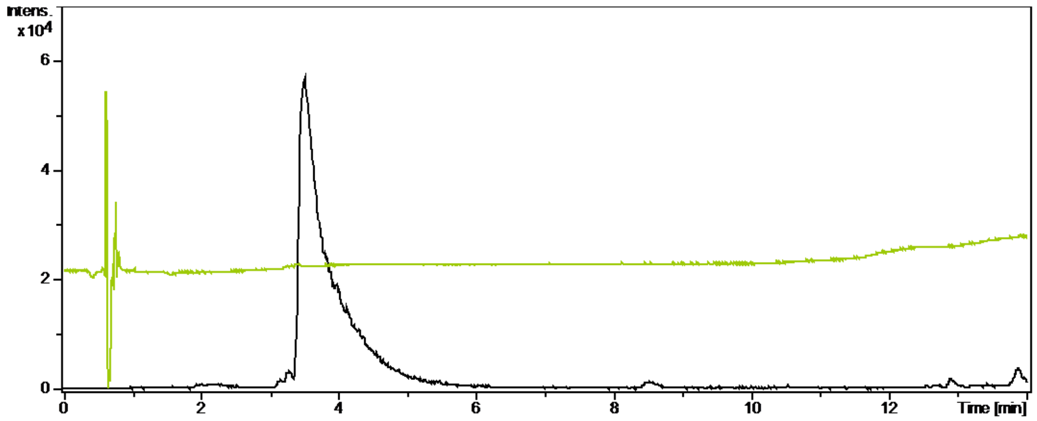

Figure S46. UHPLC/+ESI-QqTOF-MS/MS chromatogram of *N*3-chloromethyl-desacyl-epipachysamine A (**6**). Base peak chromatogram 200.0000-1000.0000 +All MS (black); UV chromatogram 200-400 nm (green).

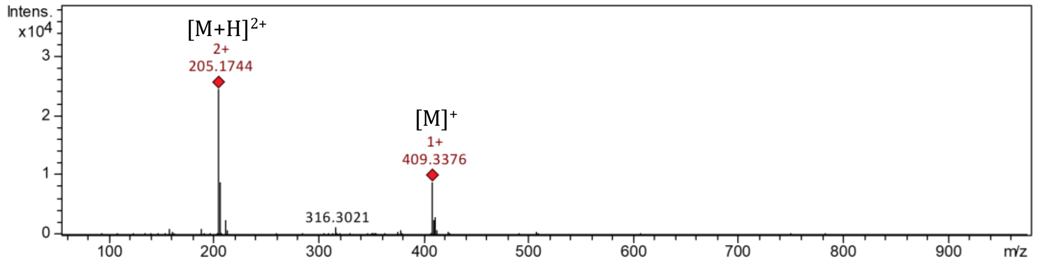

Figure S47. +ESI-QqTOF MS spectrum of *N*3-chloromethyl-desacyl-epipachysamine A (**6**); *m/z* 409.3376 $[M]^+$, *m/z* 205.1744 $[M+H]^{2+}$.

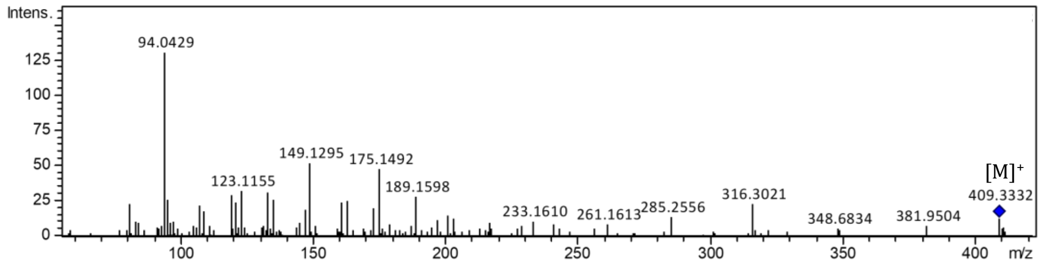

Figure S48. +ESI-QqTOF MS/MS spectrum of *N*3-chloromethyl-desacyl-epipachysamine A (**6**).

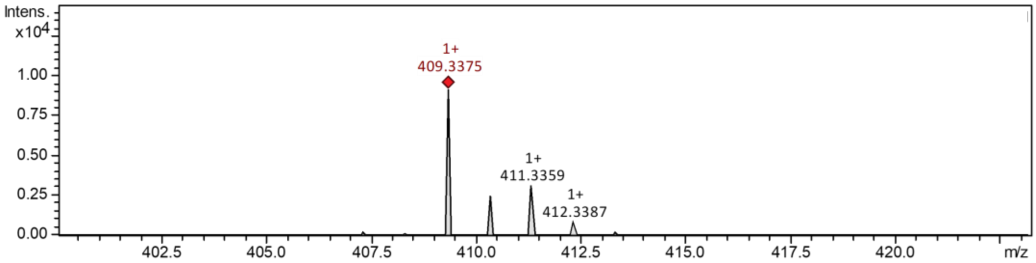

Figure S49. Detail of the +ESI-QqTOF MS spectrum of *N*3-chloromethyl-desacyl-epipachysamine A (**6**) around *m/z* 409.3376 $[M]^+$ and its isotope signals.

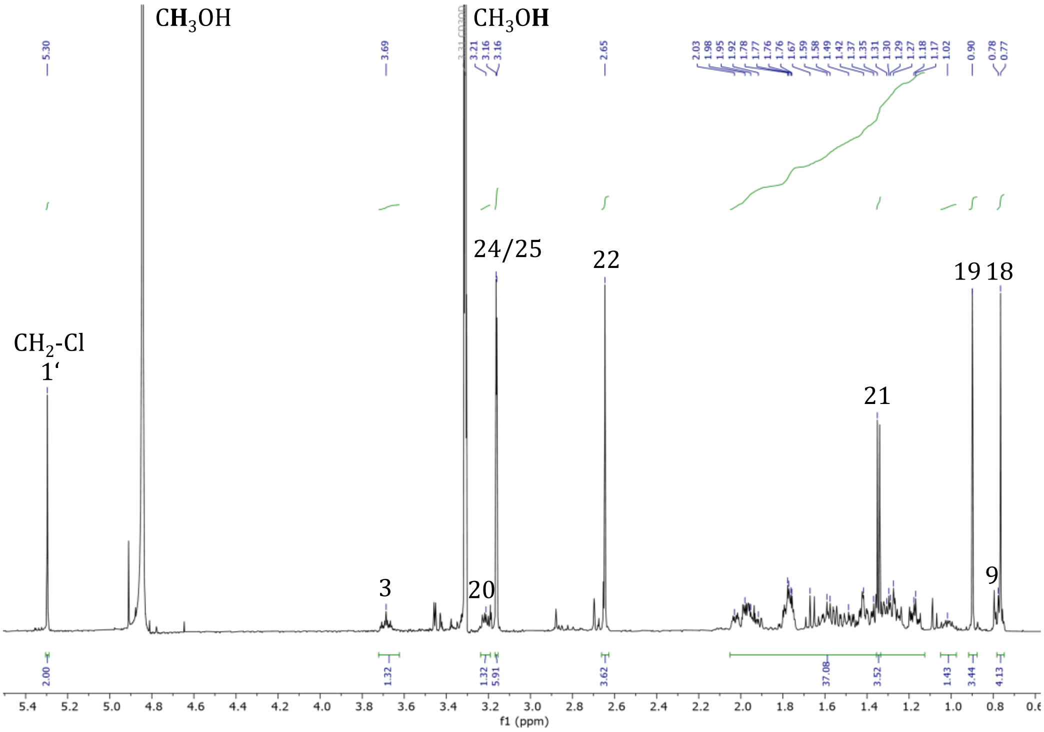

Figure S50. ^{1}H NMR spectrum of *N*3-chloromethyl-desacyl-epipachysamine A (**6**) (CD_3OD, 600 MHz). The assignment of the signals between 2.10 and 0.95 ppm can be found in the enlarged Figure S51.

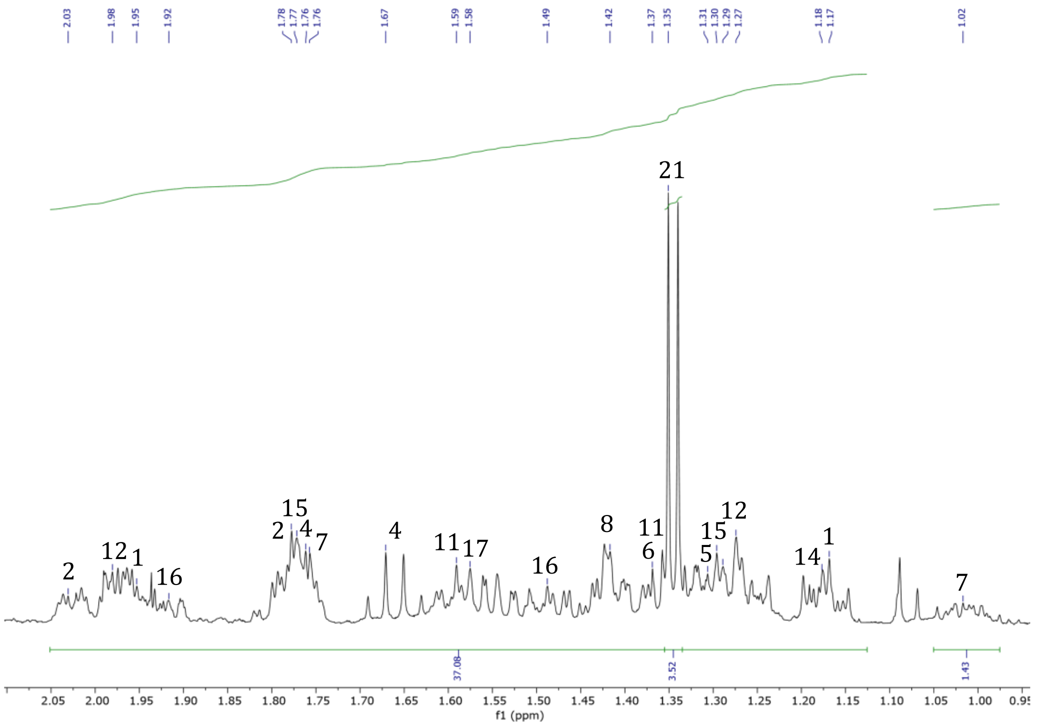

Figure S51. Detail of the ^{1}H NMR spectrum of *N*3-chloromethyl-desacyl-epipachysamine A (**6**) (CD_3OD, 600 MHz).

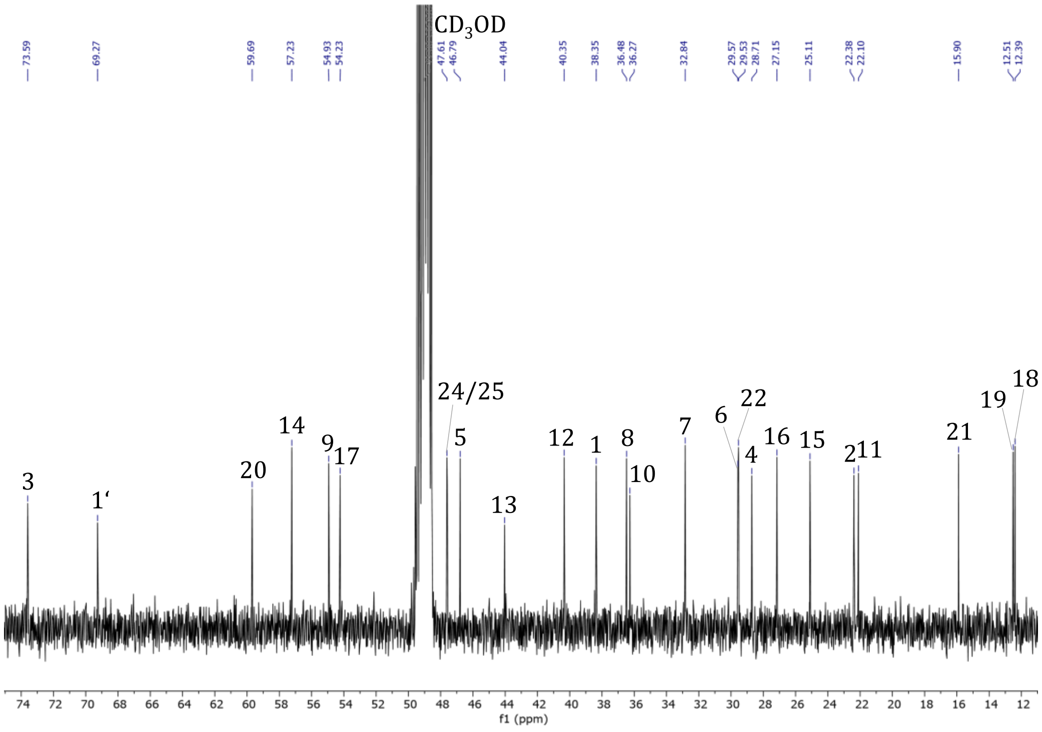

Figure S52. ^{13}C NMR spectrum of *N*3-chloromethyl-desacyl-epipachysamine A (**6**) (CD_3OD, 151 MHz).

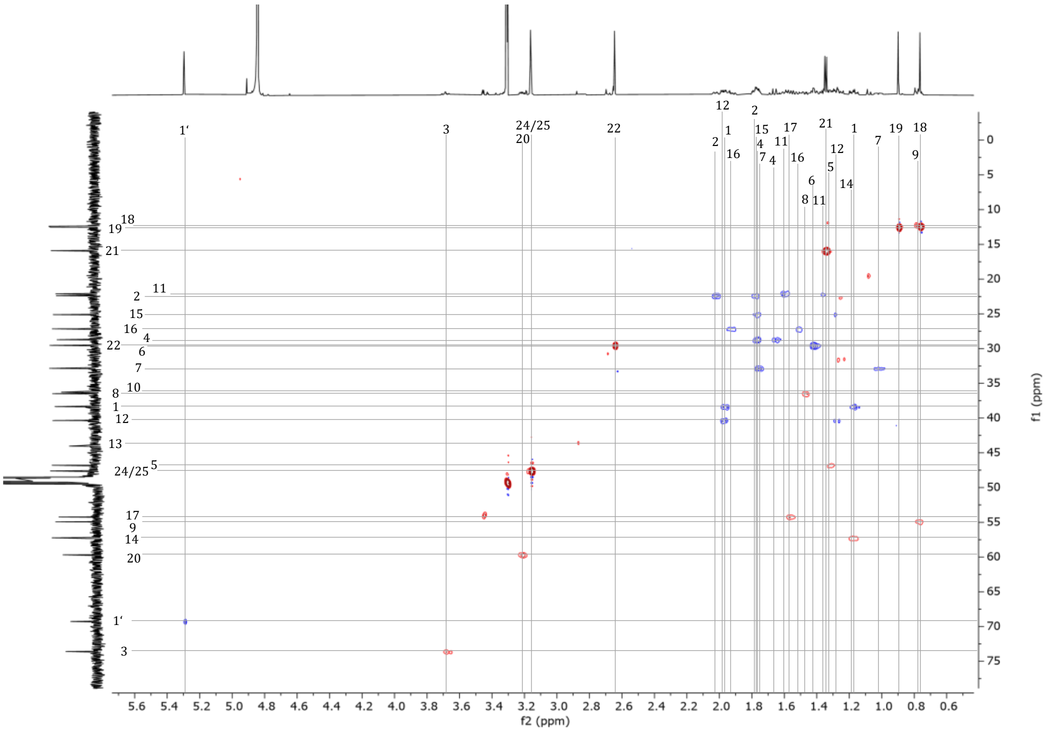

Figure S53. $^{1}H/^{13}C$ HSQC spectrum of *N*3-chloromethyl-desacyl-epipachysamine A (**6**) (CD_3OD, 600/151 MHz).

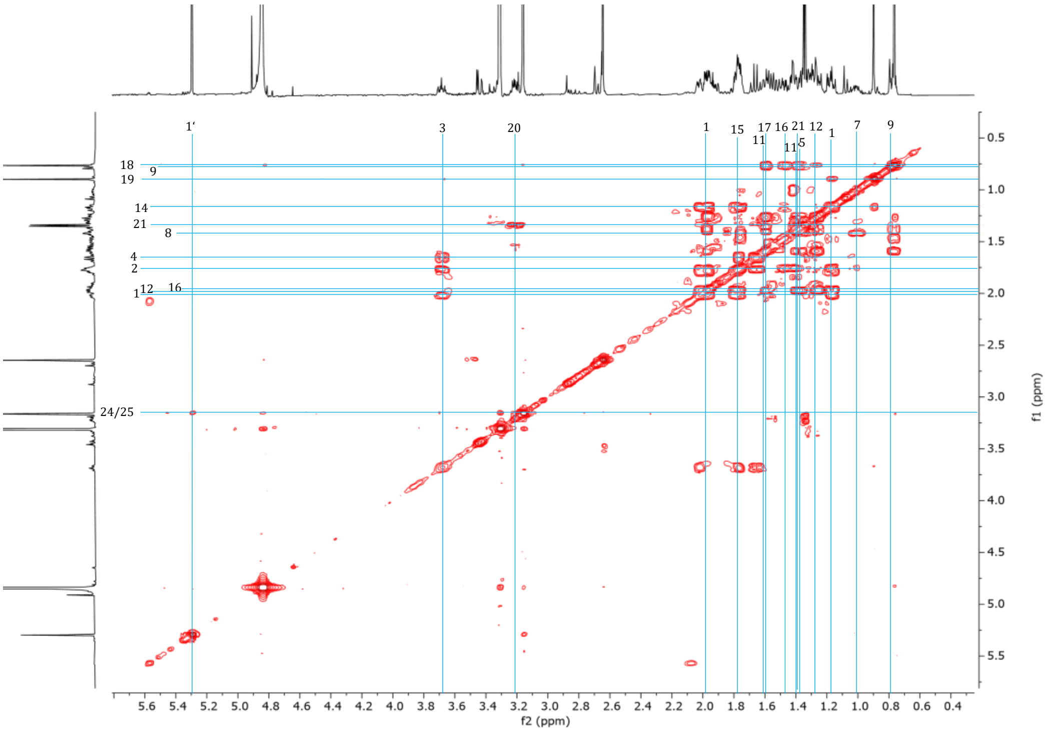

Figure S54. $^{1}H/^{1}H$ COSY spectrum of *N*3-chloromethyl-desacyl-epipachysamine A (**6**) (CD_3OD, 600 MHz).

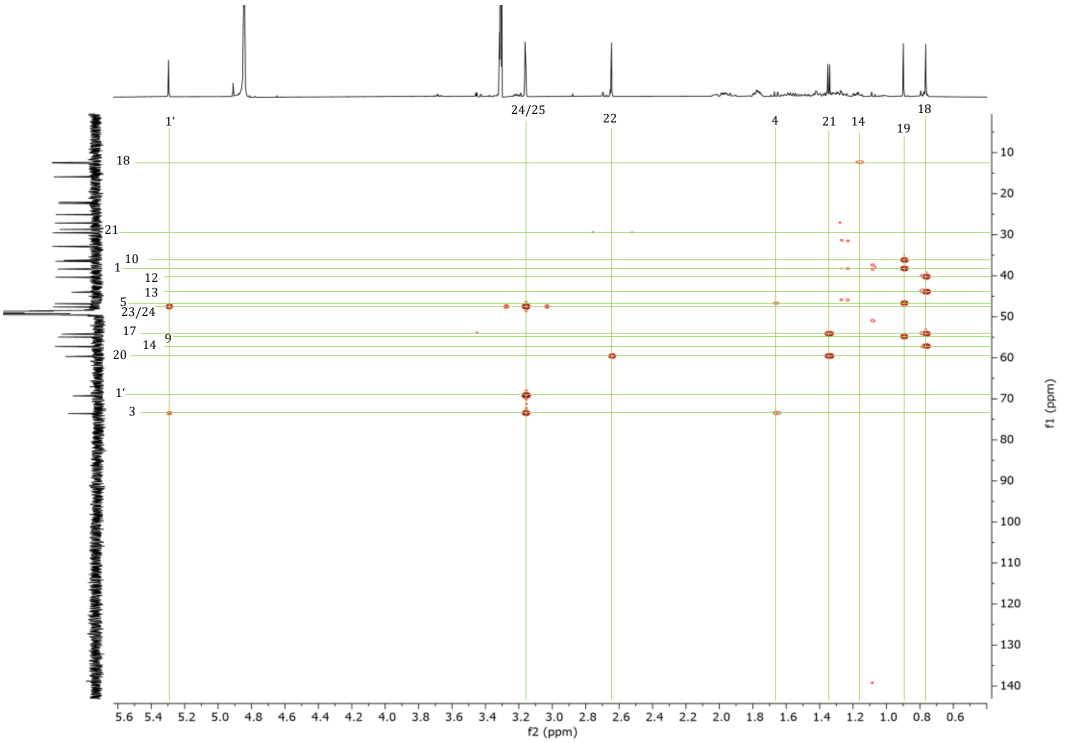

Figure S55. $^{1}H/^{13}C$ HMBC spectrum of *N*3-chloromethyl-desacyl-epipachysamine A (**6**) (CD_3OD, 600/151 MHz).

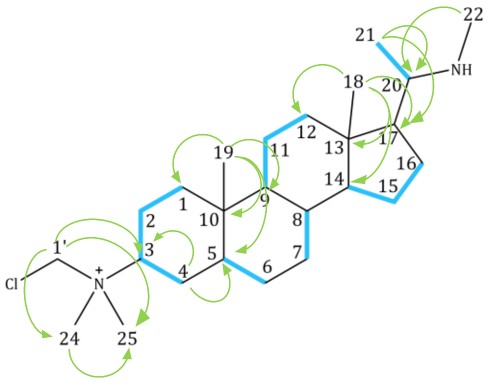

Figure S56. Key COSY (blue lines) and HMBC (green arrows) correlations of *N*3-chloromethyl-desacyl-epipachysamine A (**6**).

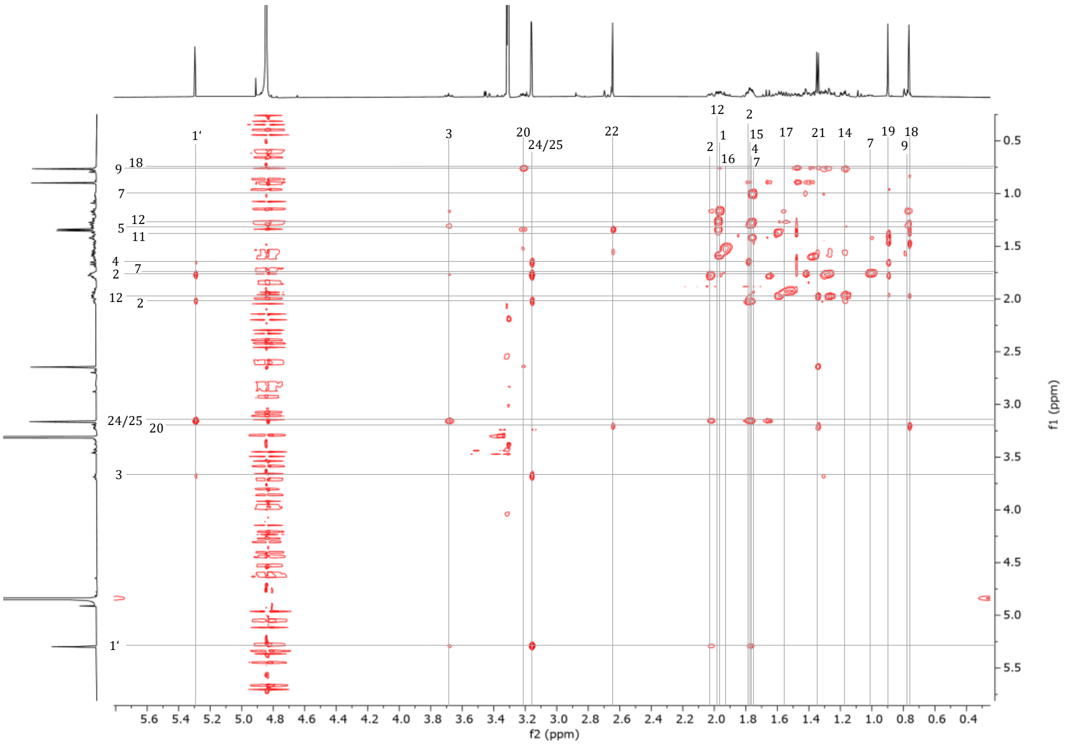

Figure S57. $^1H/^1H$ NOESY spectrum of *N*3-chloromethyl-desacyl-epipachysamine A (**6**) (CD_3OD, 600 MHz).

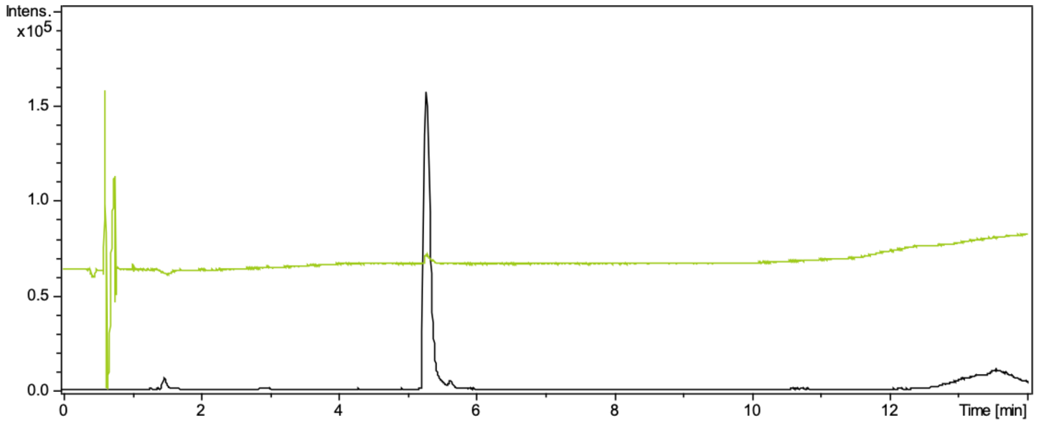

Figure S58. UHPLC/+ESI-QqTOF-MS/MS chromatogram of 3*α*,4*α*-diapachysanaximine A (**7**). Base peak chromatogram 200.0000-1000.0000 +All MS (black); UV chromatogram 200-400 nm (green).

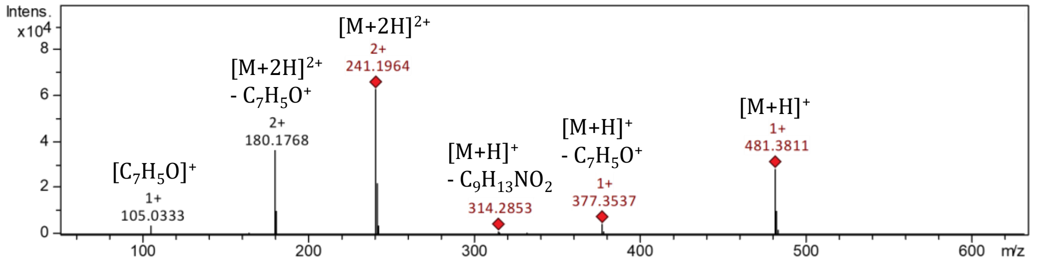

Figure S59. +ESI-QqTOF MS spectrum of 3*α*,4*α*-diapachysanaximine A (**7**); *m*/*z* 481.3811 $[M+H]^+$, *m*/*z* 241.1964 $[M+2H]^{2+}$.

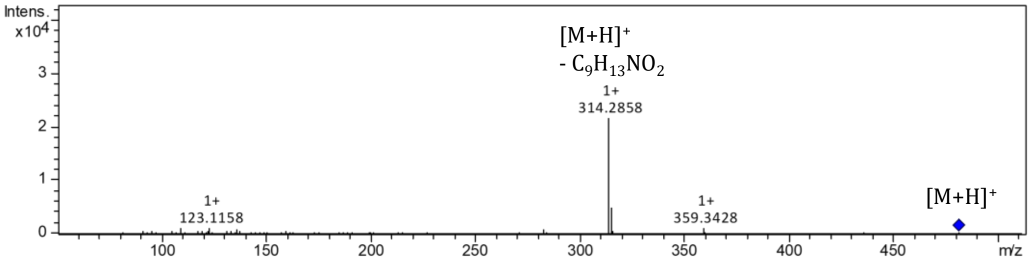

Figure S60. +ESI-QqTOF MS/MS spectrum of 3α,4α-diapachysanaximine A (**7**).

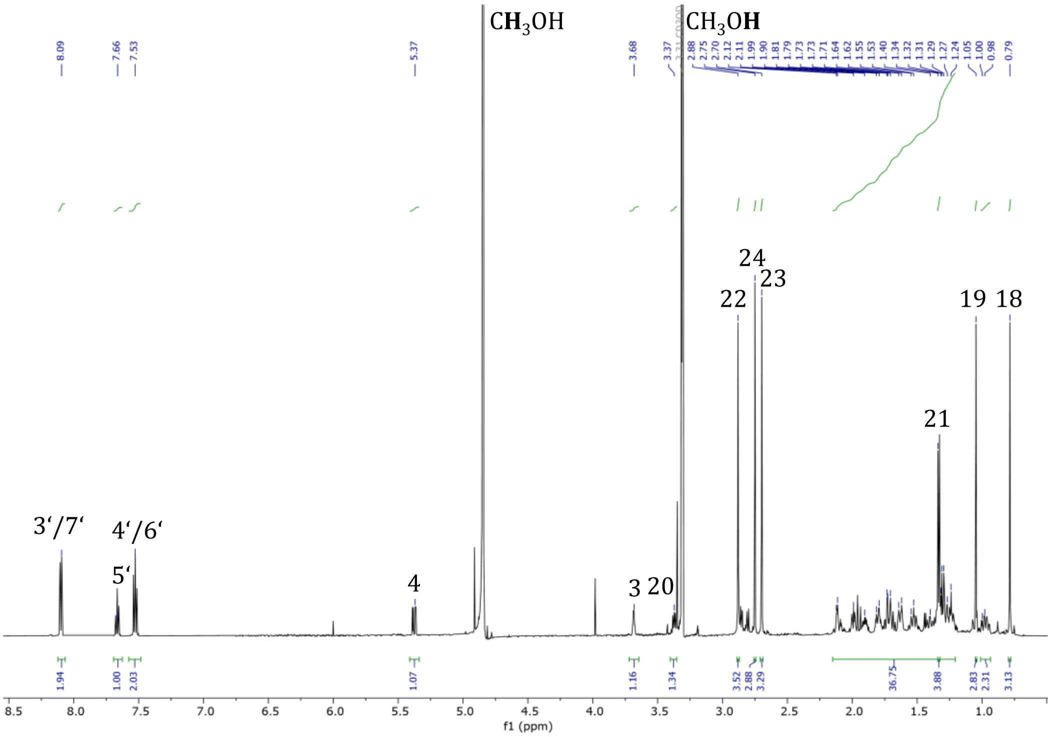

Figure S61. ^{1}H NMR spectrum of 3α,4α-diapachysanaximine A (**7**) (CD$_3$OD, 600 MHz). The assignment of the signals between 2.25 and 0.75 ppm can be found in the enlarged Figure S62.

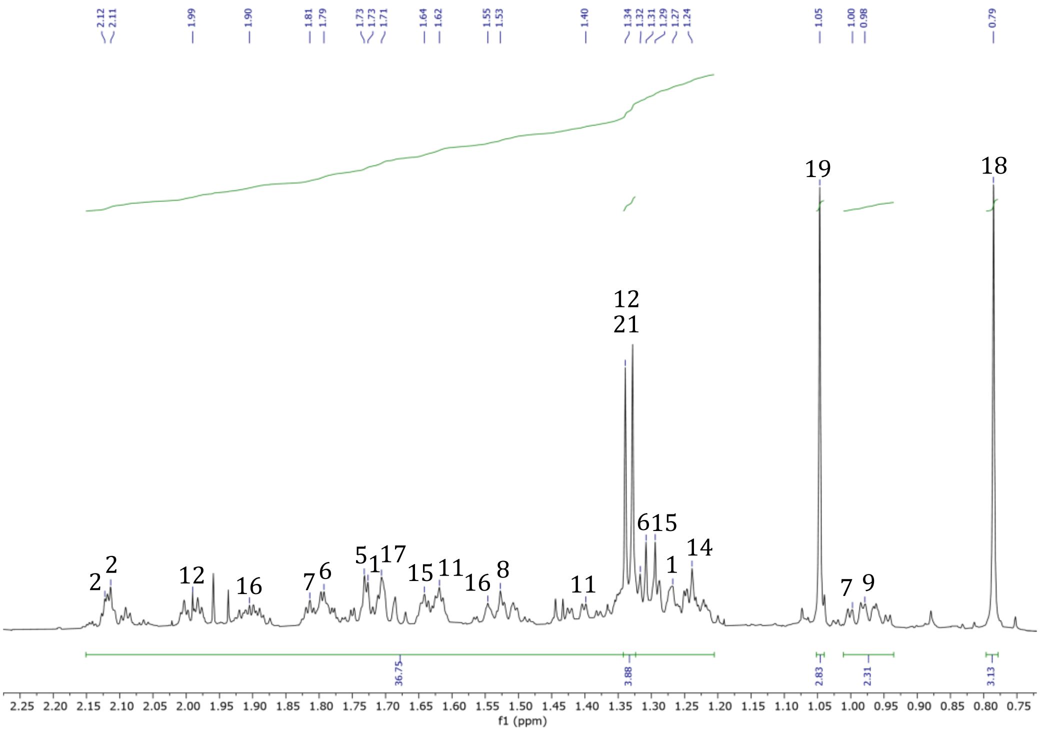

Figure S62. Detail of the ^{1}H NMR spectrum of 3α,4α-diapachysanaximine A (**7**) (CD_3OD, 600 MHz).

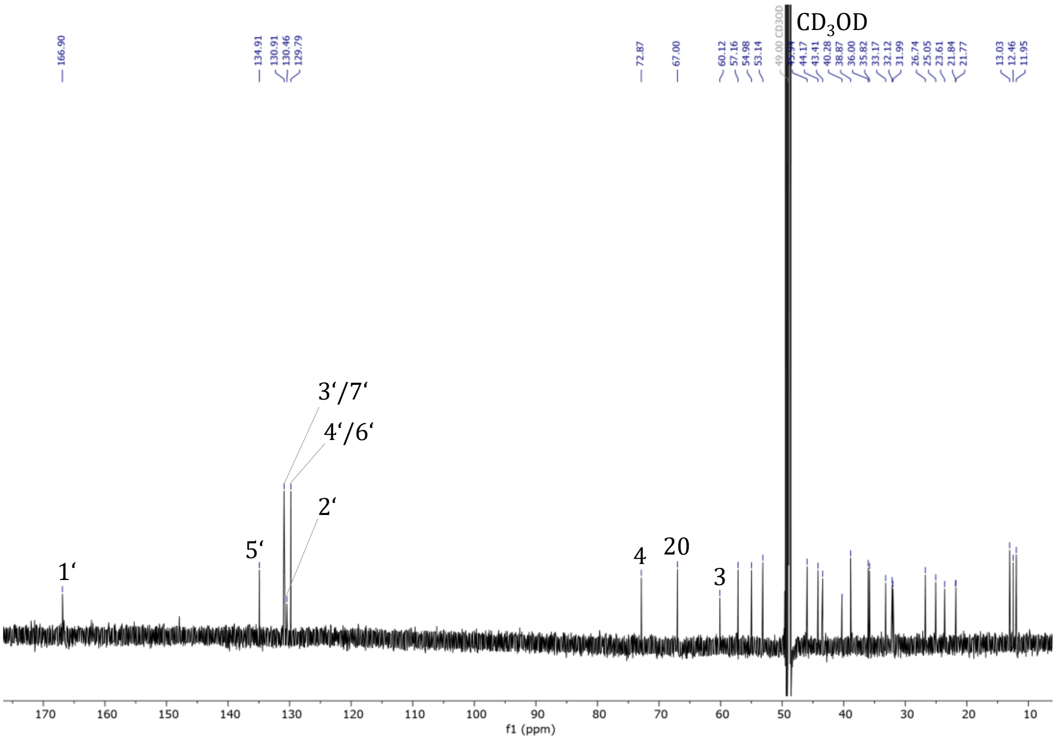

Figure S63. ^{13}C NMR spectrum of 3α,4α-diapachysanaximine A (**7**) (CD_3OD, 151 MHz). The assignment of the signals between 74 and 11 ppm can be found in the enlarged Figure S64.

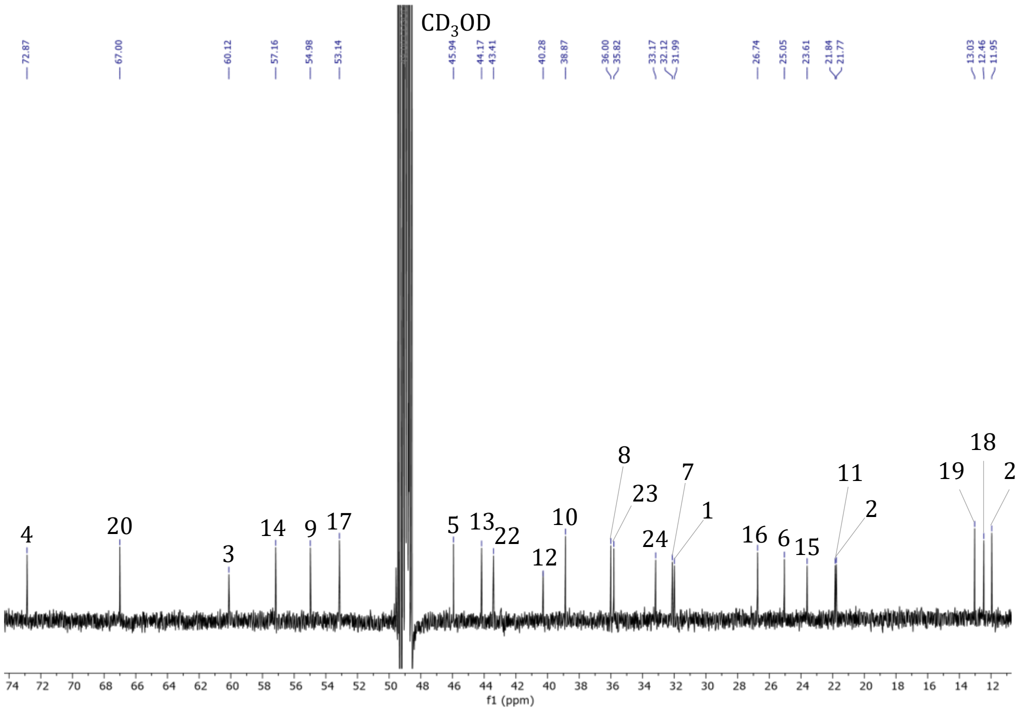

Figure S64. Detail of the ^{13}C NMR spectrum of 3α,4α-diapachysanaximine A (**7**) (CD_3OD, 151 MHz).

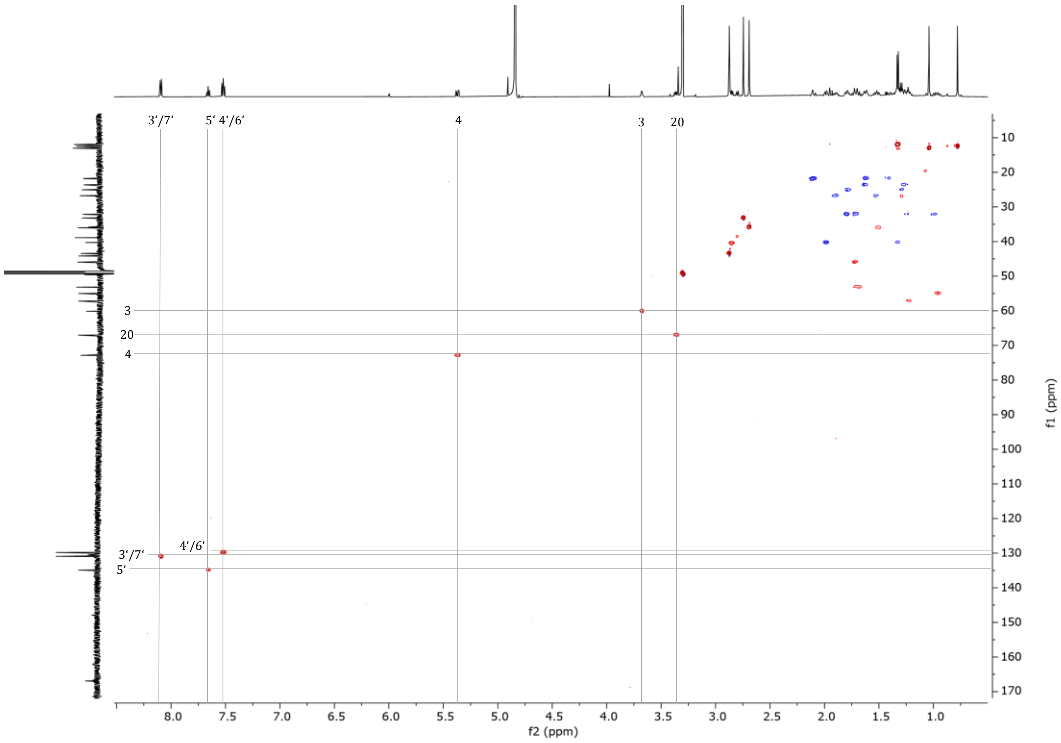

Figure S65. $^{1}H/^{13}C$ HSQC spectrum of 3α,4α-diapachysanaximine A (**7**) (CD_3OD, 600/151 MHz).

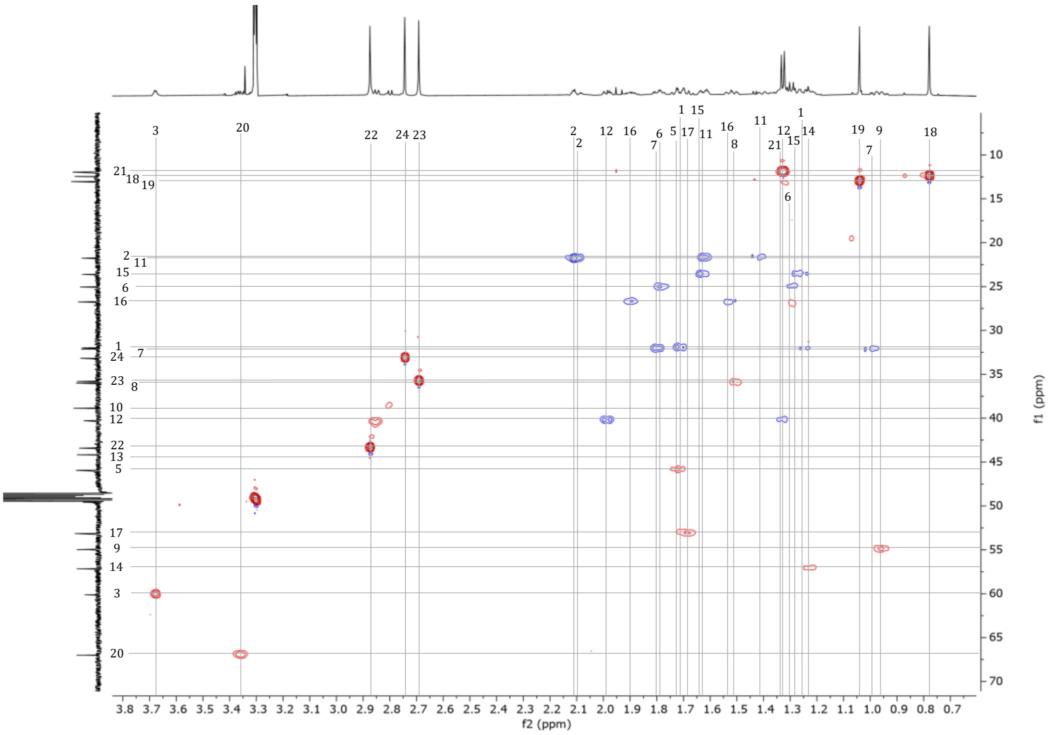

Figure S66. Detail of the $^{1}H/^{13}C$ HSQC spectrum of 3α,4α-diapachysanaximine A (**7**) (CD_3OD, 600/151 MHz).

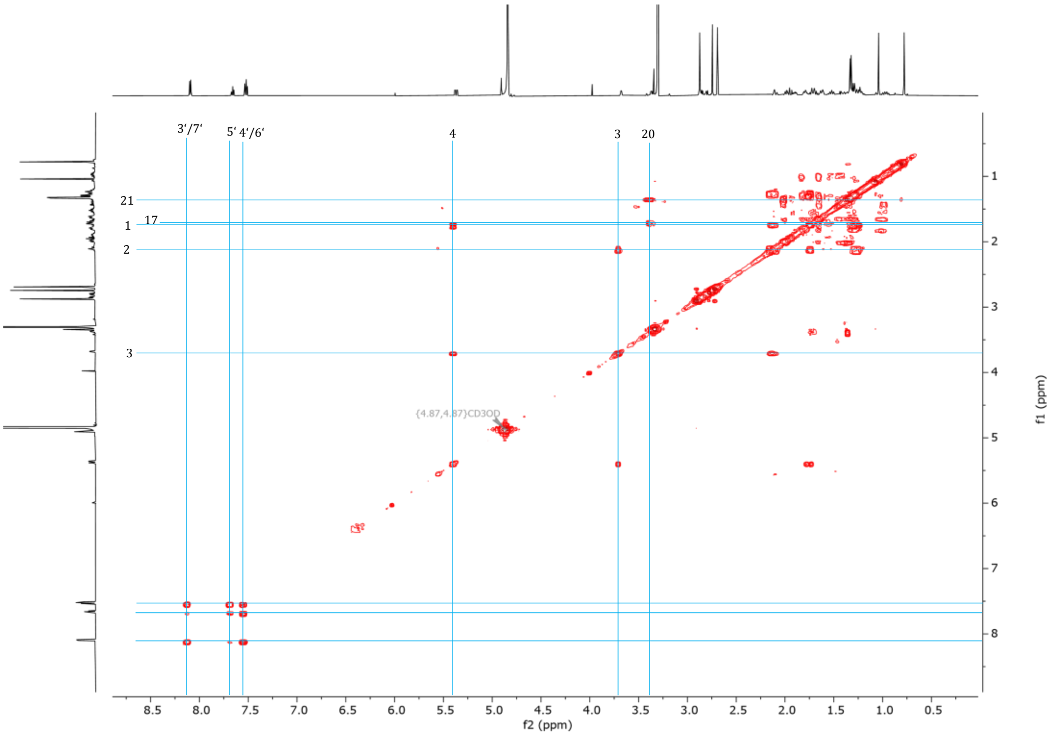

Figure S67. $^{1}H/^{1}H$ COSY spectrum of 3α,4α-diapachysanaximine A (**7**) (CD_3OD, 600 MHz).

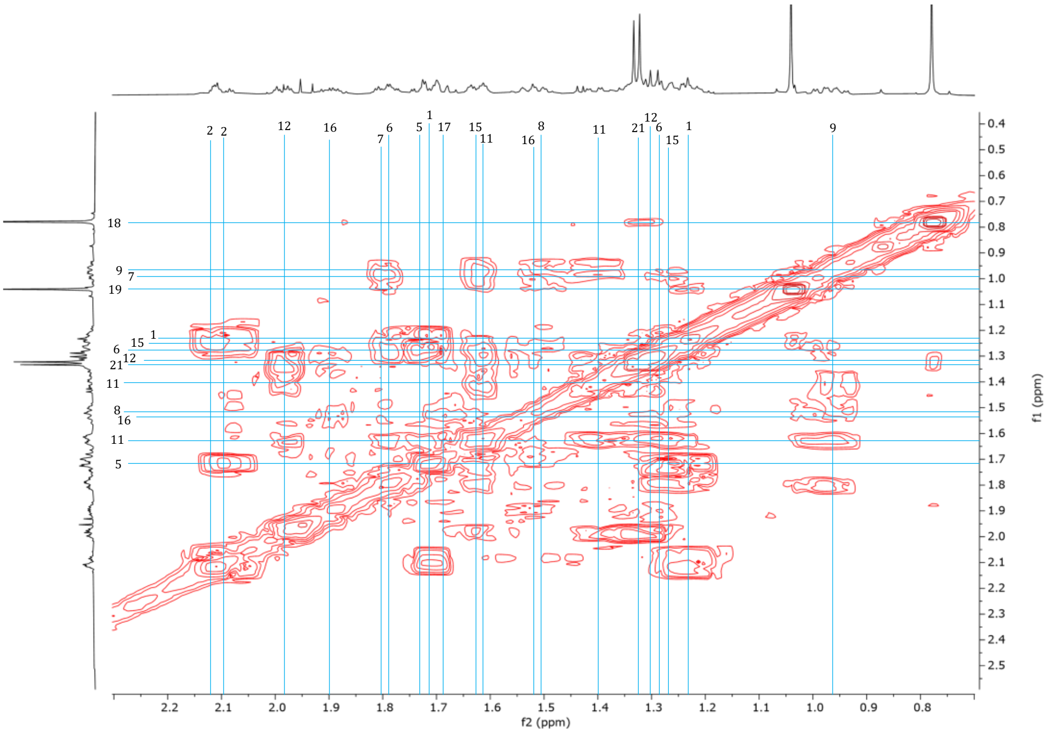

Figure S68. Detail of the $^1H/^1H$ COSY spectrum of 3α,4α-diapachysanaximine A (**7**) (CD_3OD, 600 MHz).

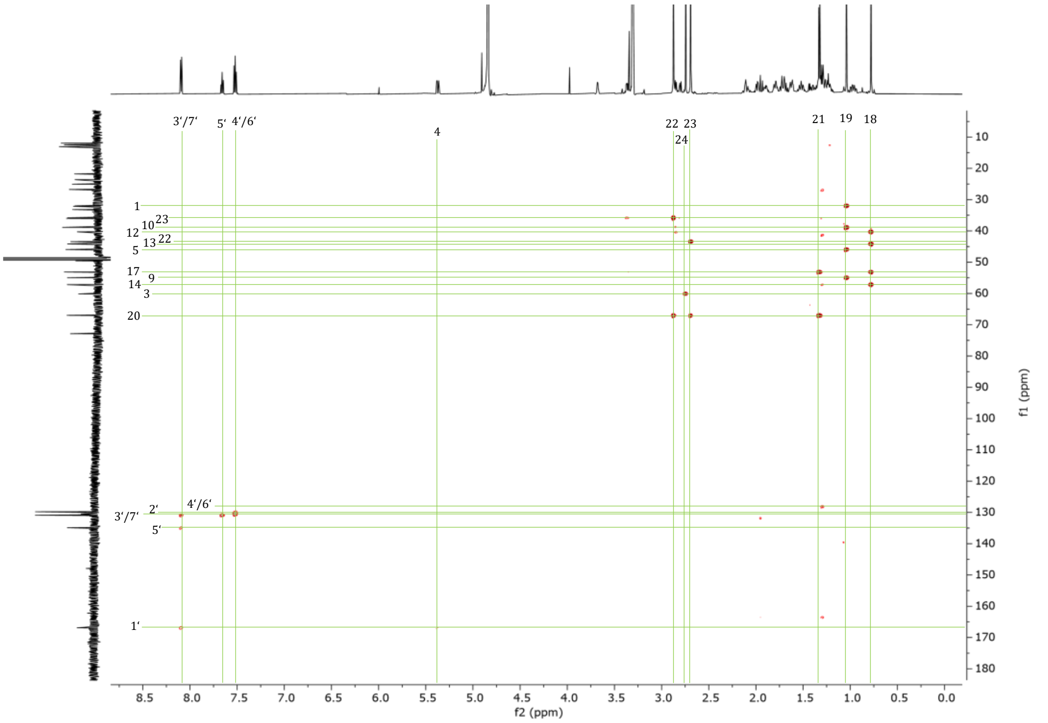

Figure S69. $^1H/^{13}C$ HMBC spectrum of 3α,4α-diapachysanaximine A (**7**) (CD_3OD, 600/151 MHz).

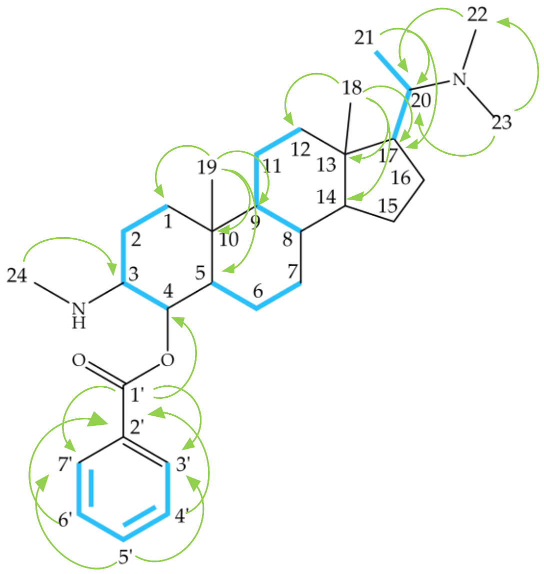

Figure S70. Key COSY (blue lines) and HMBC (green arrows) correlations of 3*α*,4*α*-diapachysanaximine A (**7**).

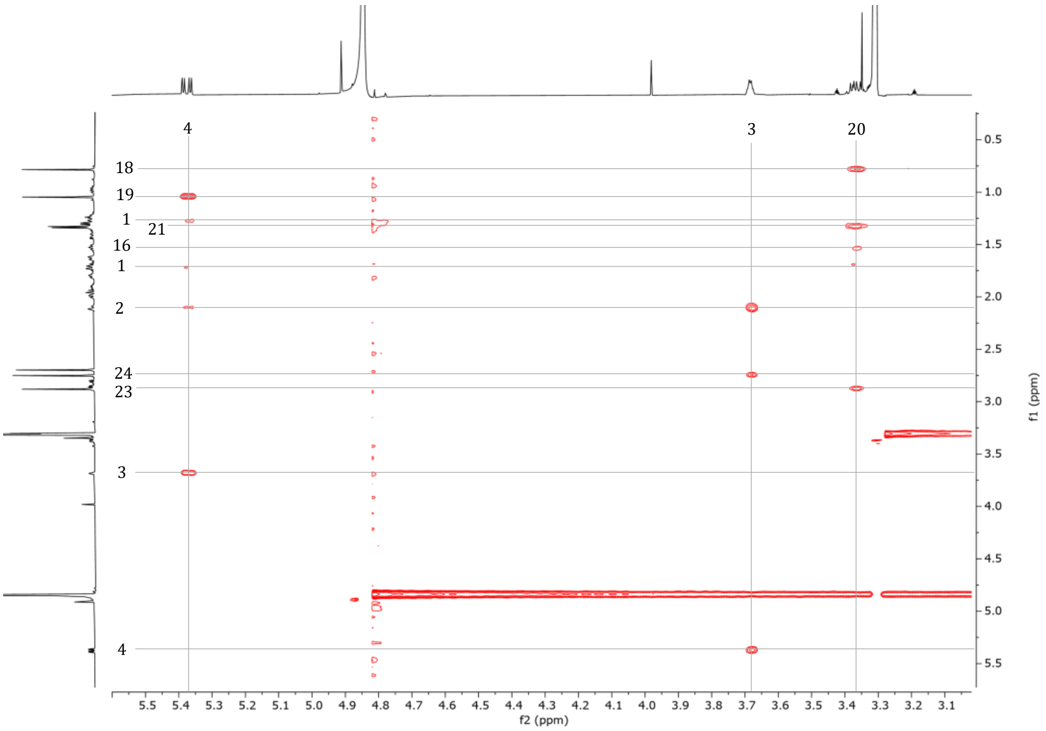

Figure S71. ^{1}H/^{1}H NOESY spectrum of 3*α*,4*α*-diapachysanaximine A (**7**) (CD_3OD, 600 MHz).

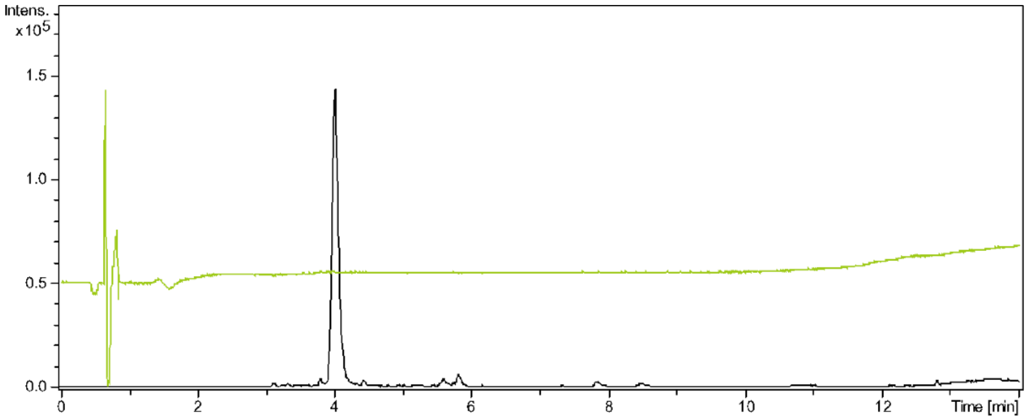

Figure S72. UHPLC/+ESI-QqTOF-MS/MS chromatogram of pachysamine A (**8**). Base peak chromatogram 200.0000-1000.0000 +All MS (black); UV chromatogram 200-400 nm (green).

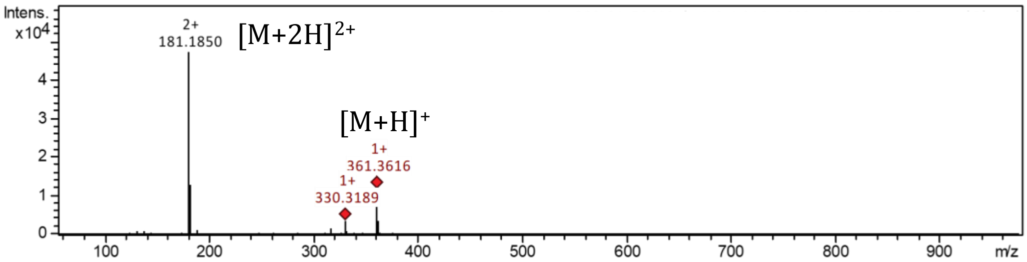

Figure S73. +ESI-QqTOF MS spectrum of pachysamine A (**8**); *m/z* 361.3616 $[M+H]^+$, *m/z* 181.1850 $[M+2H]^{2+}$.

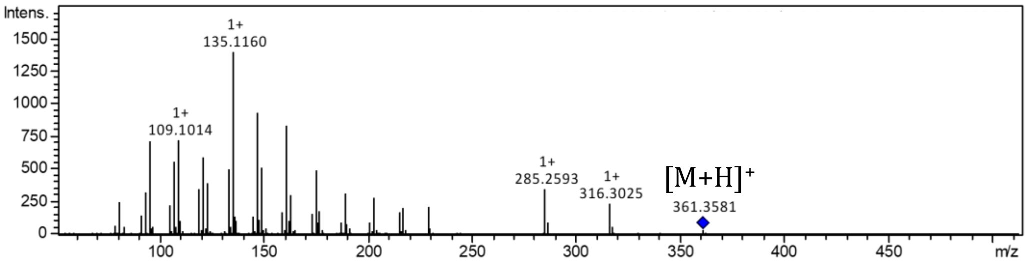

Figure S74. +ESI-QqTOF MS/MS spectrum of pachysamine A (**8**).

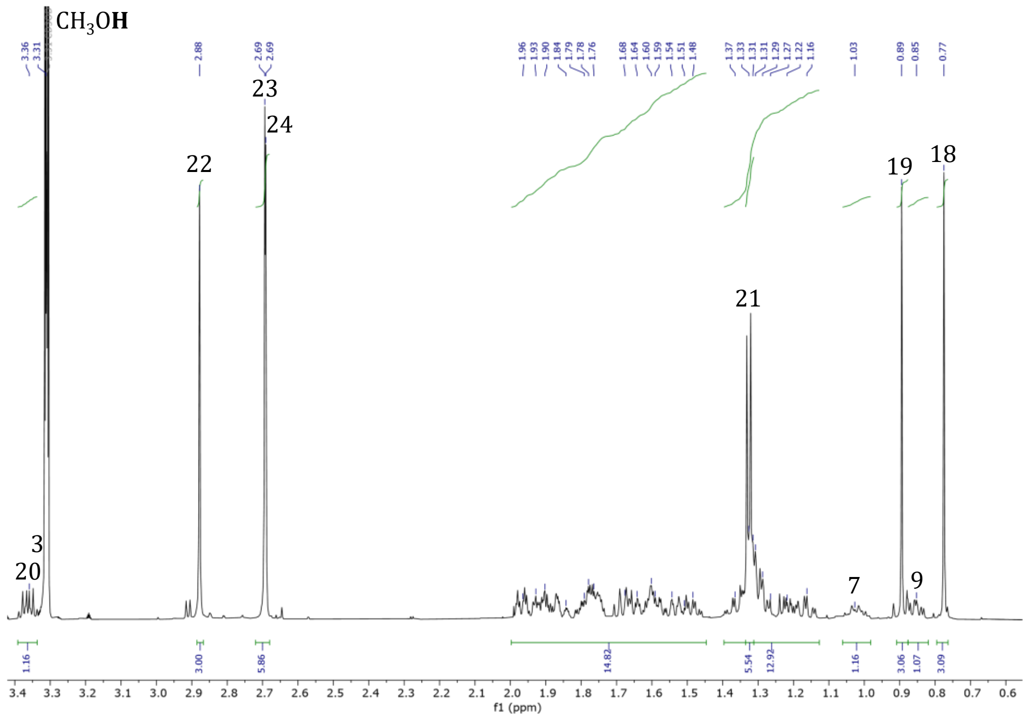

Figure S75. ^{1}H NMR spectrum of pachysamine A (**8**) (CD_3OD, 600 MHz). The assignment of the signals between 2.05 and 0.95 ppm can be found in the enlarged Figure S76.

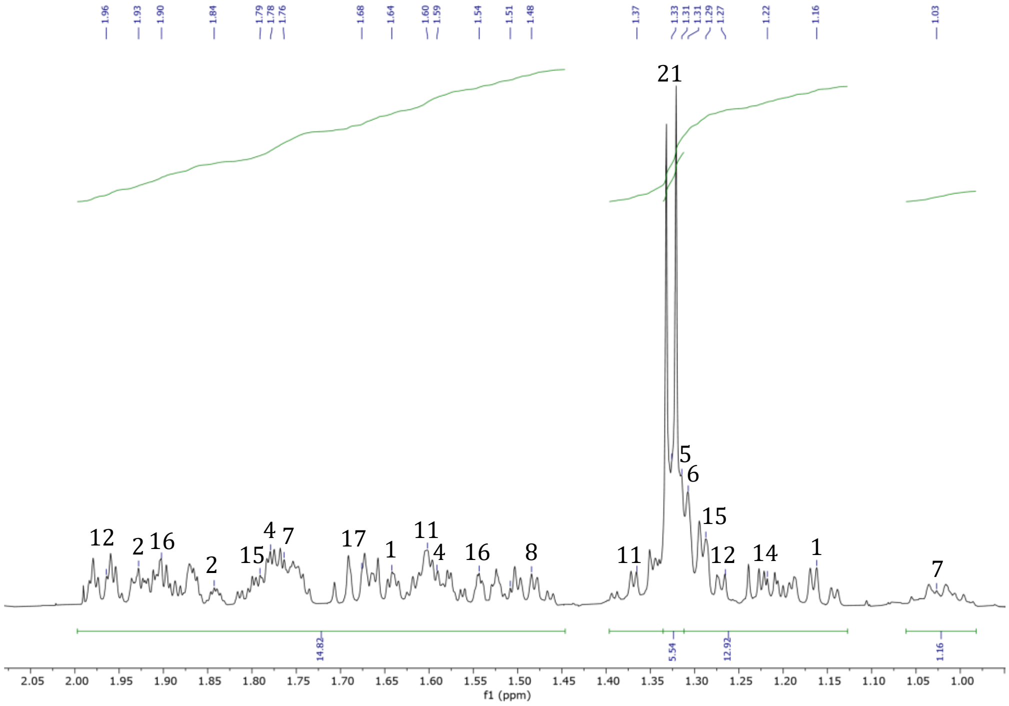

Figure S76. Detail of the ^{1}H NMR spectrum of pachysamine A (**8**) (CD_3OD, 600 MHz).

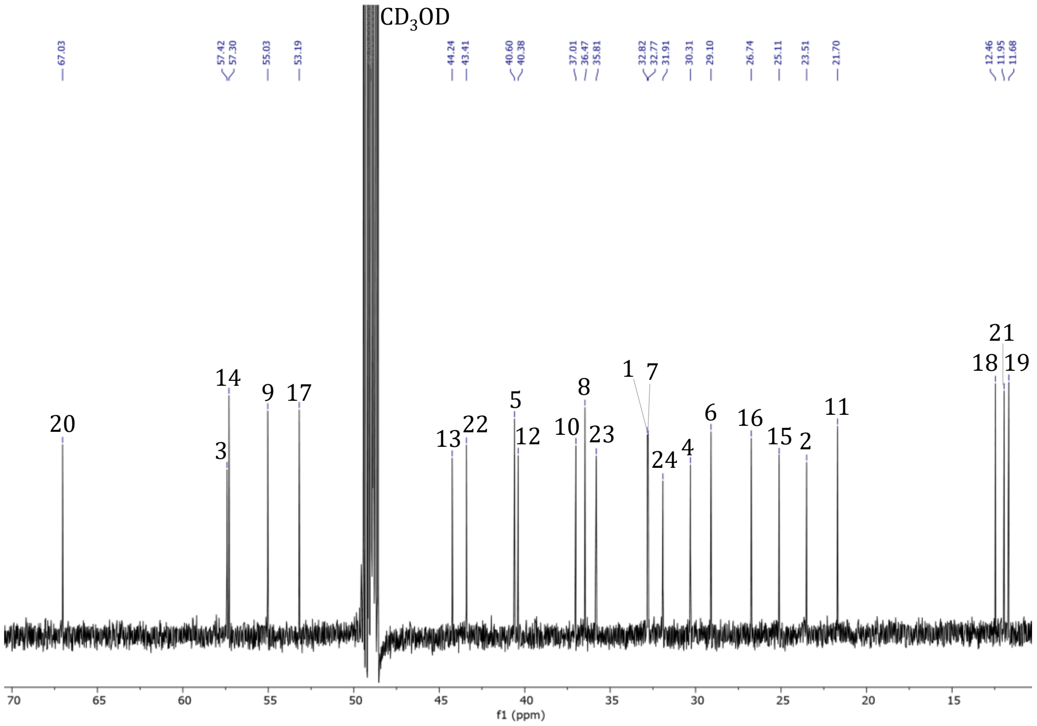

Figure S77. ^{13}C NMR spectrum of pachysamine A (**8**) (CD_3OD, 151 MHz).

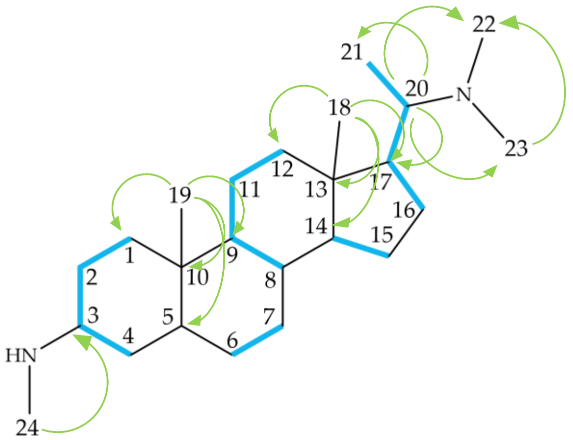

Figure S78. Key COSY (blue lines) and HMBC (green arrows) correlations of pachysamine A (**8**).

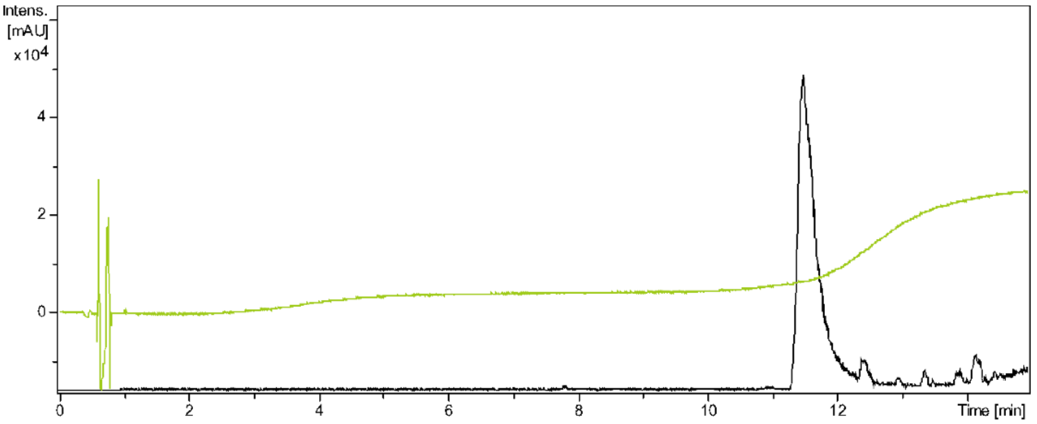

Figure S79. UHPLC/+ESI-QqTOF-MS/MS chromatogram of 3β-dimethylamino-pregnane-20-one (**9**). Base peak chromatogram 200.0000-1000.0000 +All MS (black); UV chromatogram 200-400 nm (green).

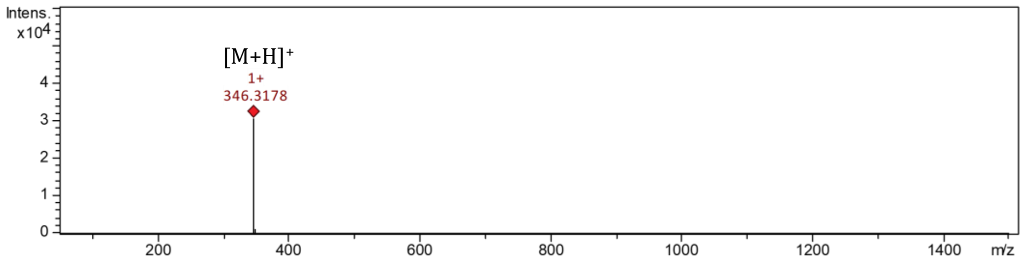

Figure S80. +ESI-QqTOF MS spectrum of 3β-dimethylamino-pregnane-20-one (**9**); *m/z* 346.3178 $[M+H]^+$.

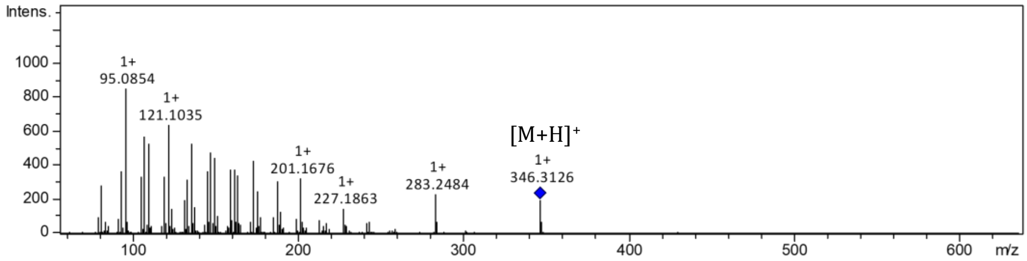

Figure S81. +ESI-QqTOF MS/MS spectrum of 3β-dimethylamino-pregnane-20-one (**9**).

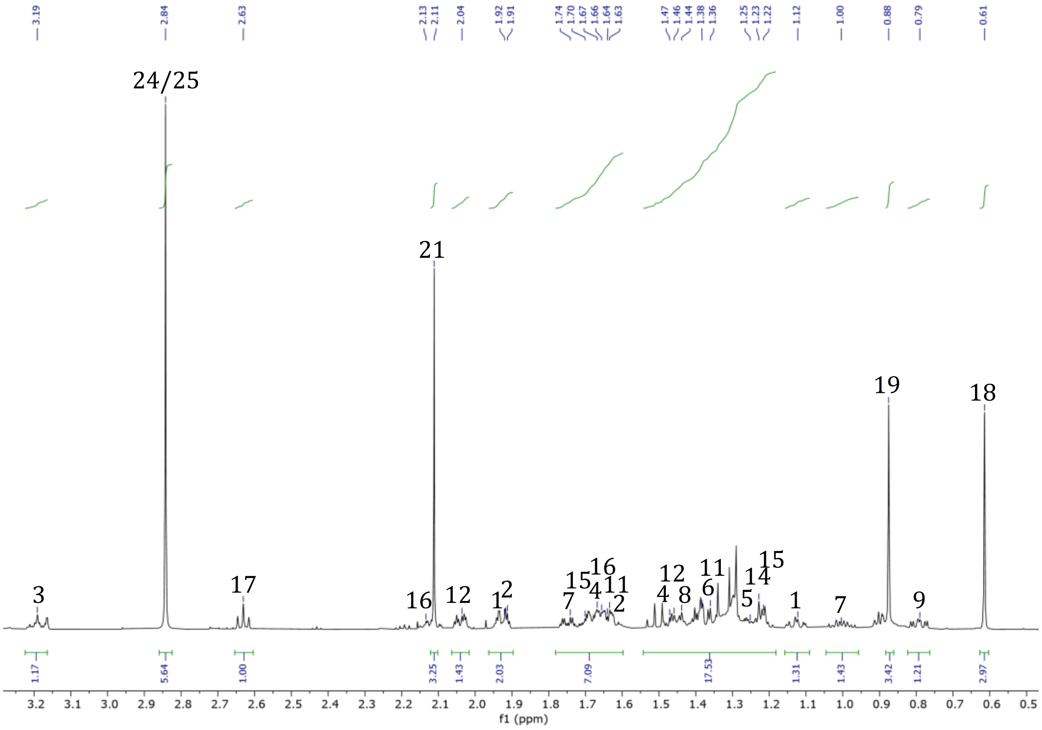

Figure S82. ^{1}H NMR spectrum of 3β-dimethylamino-pregnane-20-one (**9**) (CD_3OD, 600 MHz).

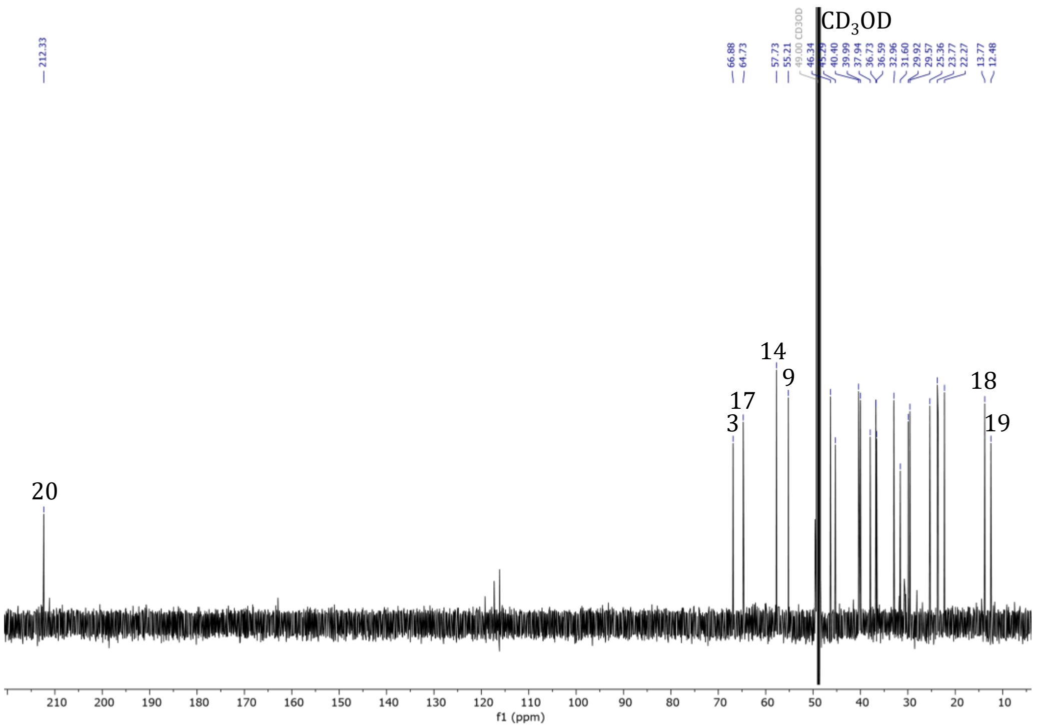

Figure S83. ^{13}C NMR spectrum of 3β-dimethylamino-pregnane-20-one (**9**) (CD_3OD, 151 MHz). The assignment of the signals between 69 and 11 ppm can be found in the enlarged Figure S84.

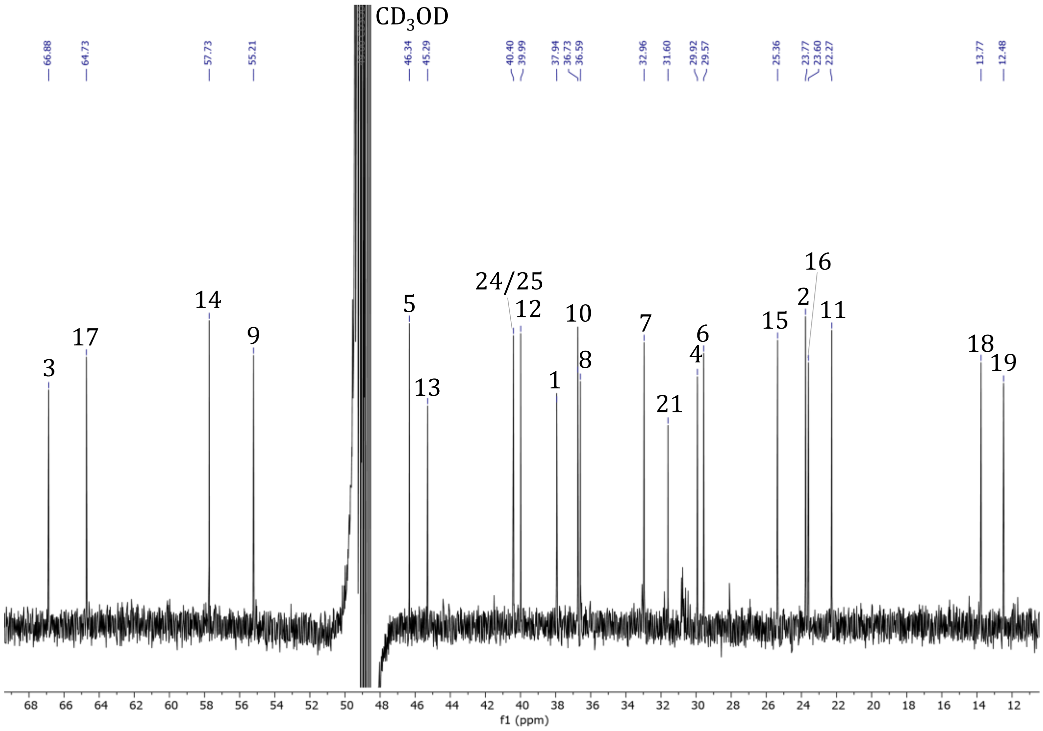

Figure S84. Detail of the ^{13}C NMR spectrum of 3β-dimethylamino-pregnane-20-one (**9**) (CD_3OD, 151 MHz).

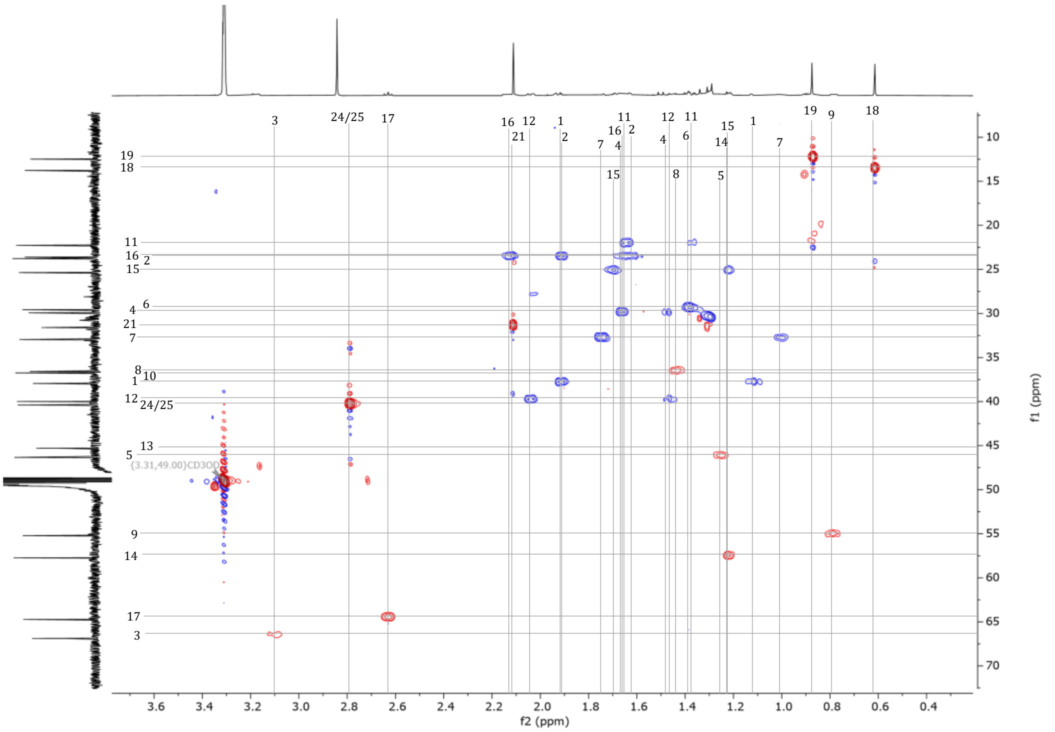

Figure S85. $^{1}H/^{13}C$ HSQC spectrum of 3β-dimethylamino-pregnane-20-one (**9**) (CD_3OD, 600/151 MHz).

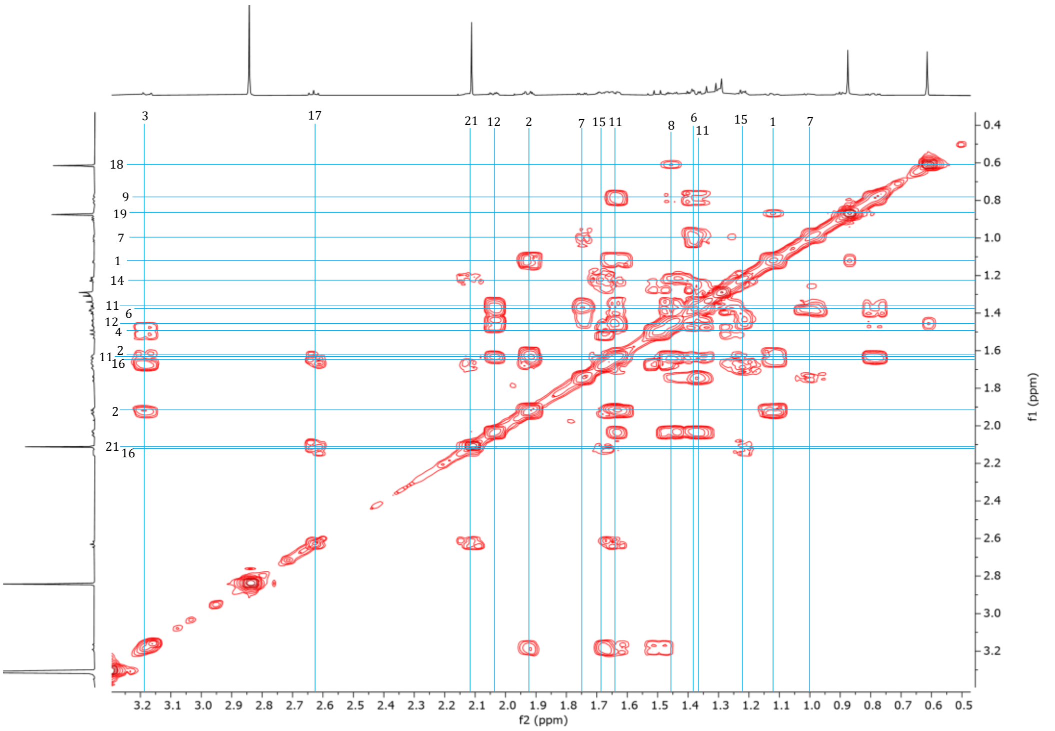

Figure S86. $^{1}H/^{1}H$ COSY spectrum of 3β-dimethylamino-pregnane-20-one (**9**) (CD_3OD, 600 MHz).

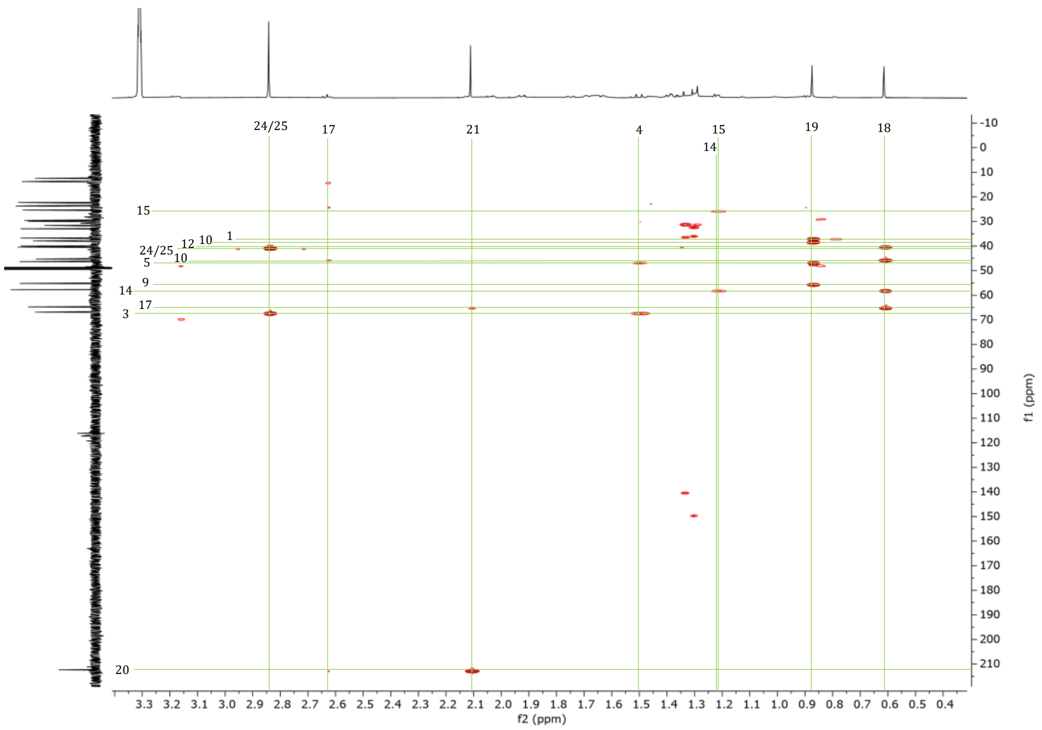

Figure S87. $^1H/^{13}C$ HMBC spectrum of 3β-dimethylamino-pregnane-20-one (**9**) (CD_3OD, 600/151 MHz).

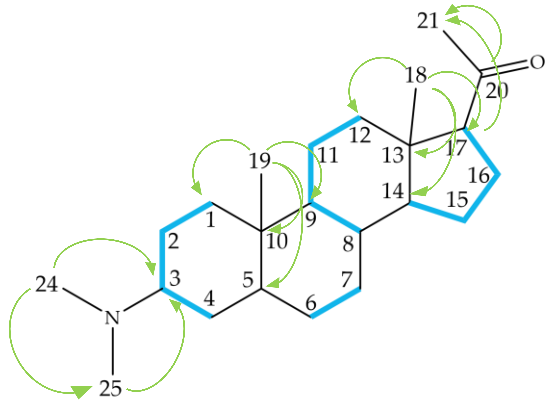

Figure S88. Key COSY (blue lines) and HMBC (green arrows) correlations of 3β-dimethyl-amino-pregnane-20-one (**9**).

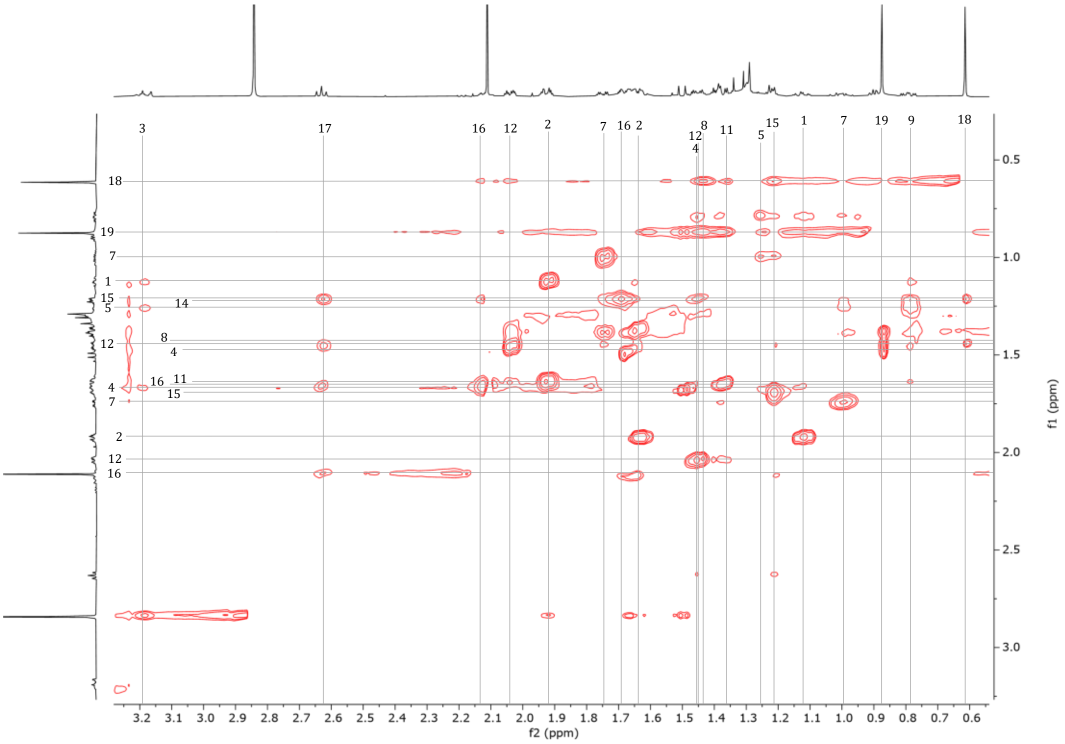

Figure S89. $^{1}H/^{1}H$ NOESY spectrum of 3β-dimethylamino-pregnane-20-one (**9**) (CD_3OD, 600 MHz).

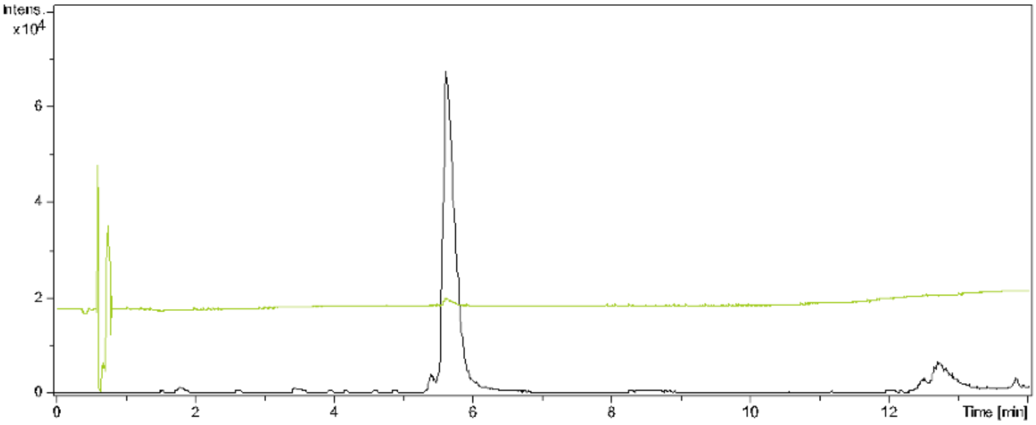

Figure S90. UHPLC/+ESI-QqTOF-MS/MS chromatogram of pachysandrine D (**10**). Base peak chromatogram 200.0000-1000.0000 +All MS (black); UV chromatogram 200-400 nm (green).

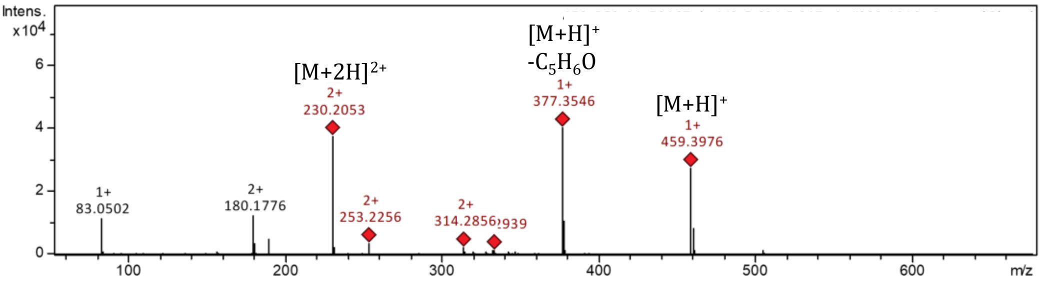

Figure S91. +ESI-QqTOF MS spectrum of pachysandrine D (**10**); *m/z* 459.3976 $[M+H]^+$, *m/z* 230.2053 $[M+2H]^{2+}$.

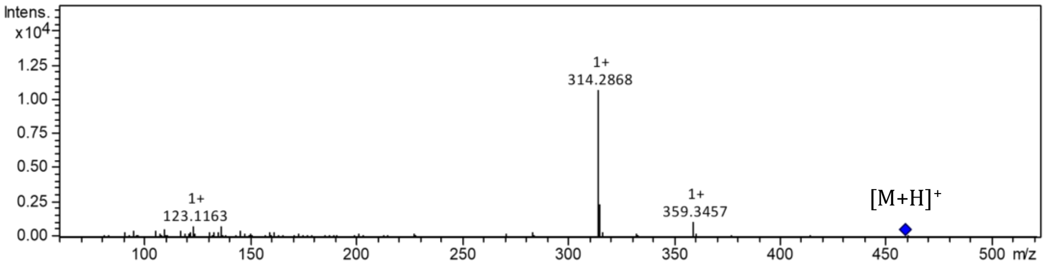

Figure S92. +ESI-QqTOF MS/MS spectrum of pachysandrine D (**10**).

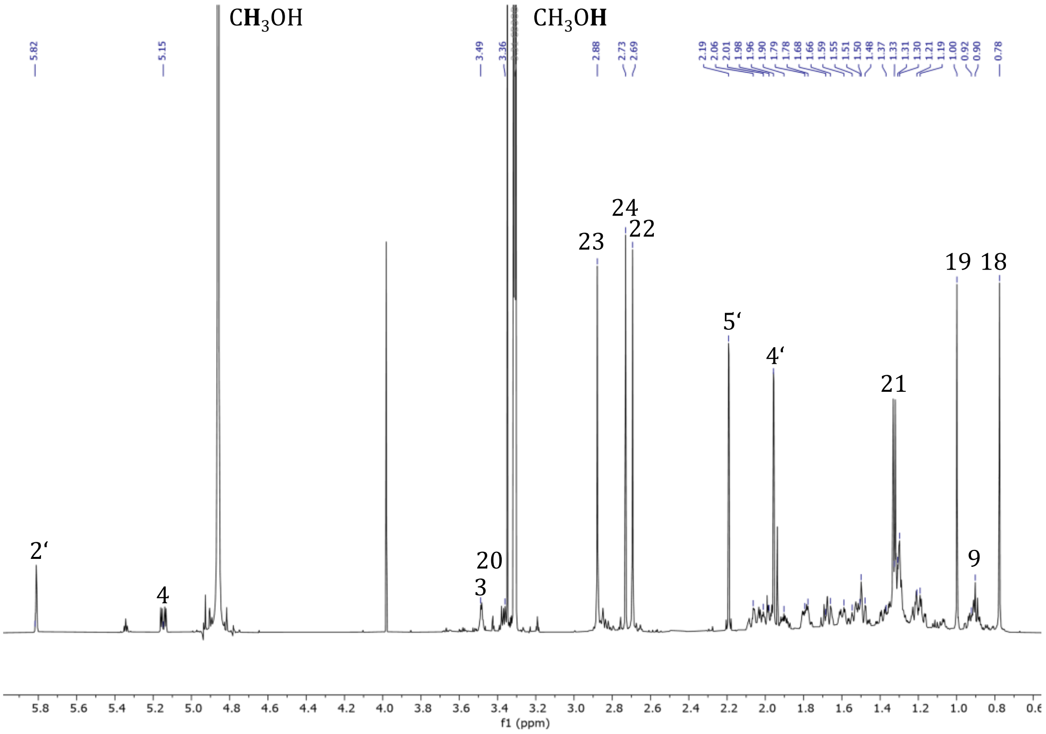

Figure S93. ^{1}H NMR spectrum of pachysandrine D (**10**) (CD_3OD, 600 MHz). The assignment of the signals between 2.25 and 0.70 ppm can be found in the enlarged Figure S94.

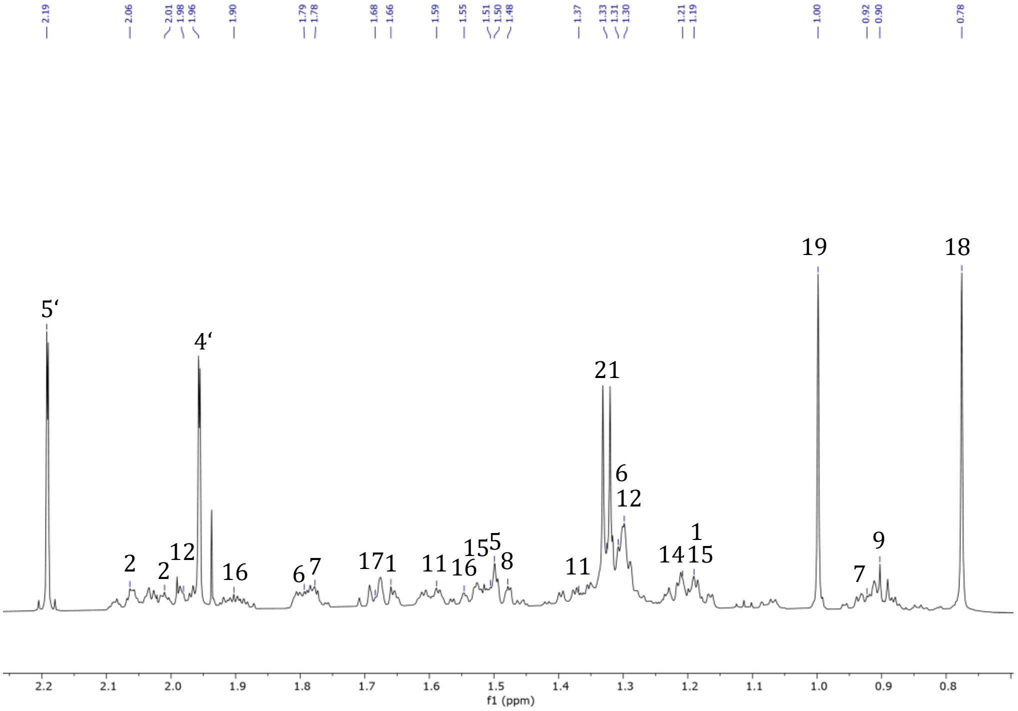

Figure S94. Detail of the ^{1}H NMR spectrum of pachysandrine D (**10**) (CD_3OD, 600 MHz).

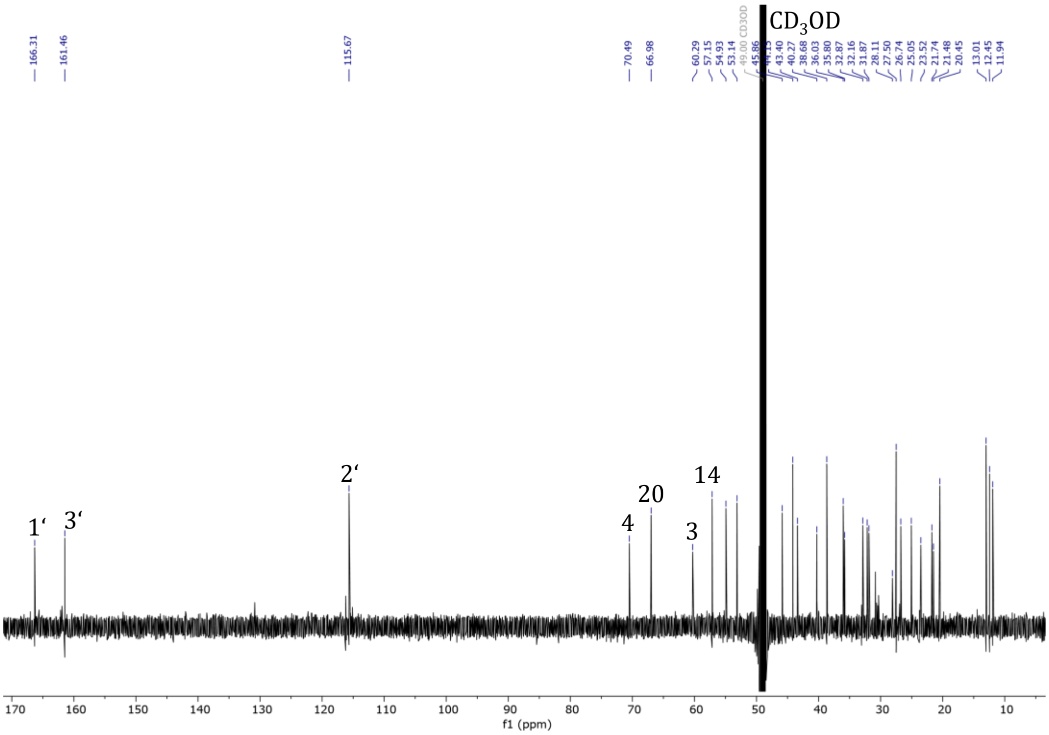

Figure S95. ^{13}C NMR spectrum of pachysandrine D (**10**) (CD_3OD, 151 MHz). The assignment of the signals between 72 and 11 ppm can be found in the enlarged Figure S96.

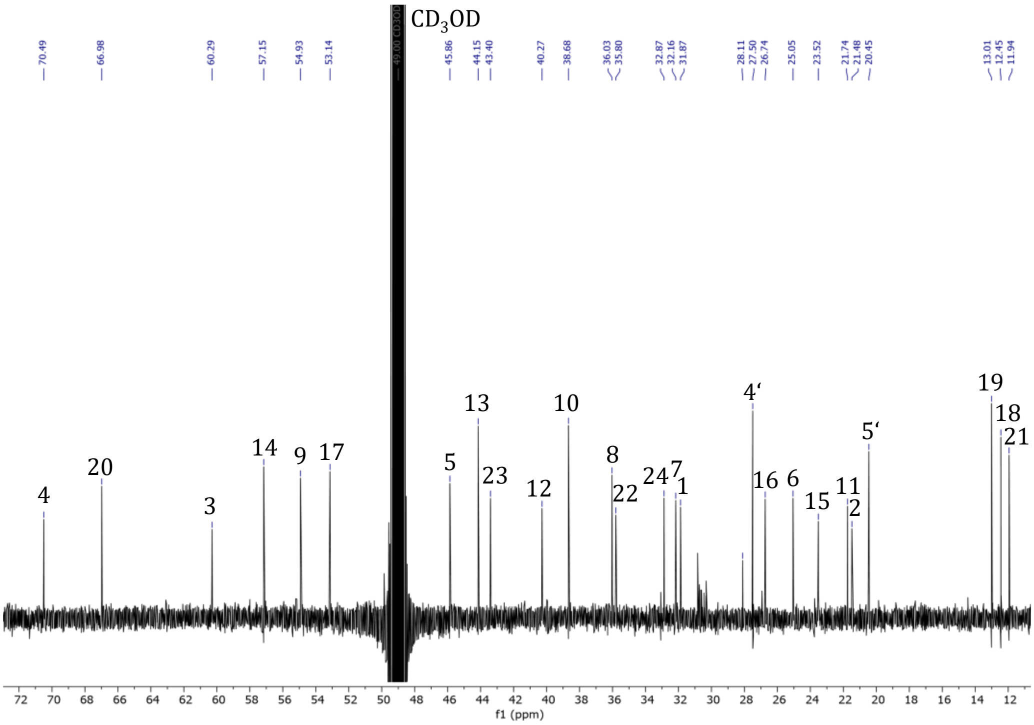

Figure S96. Detail of the ^{13}C NMR spectrum of pachysandrine D (**10**) (CD_3OD, 151 MHz).

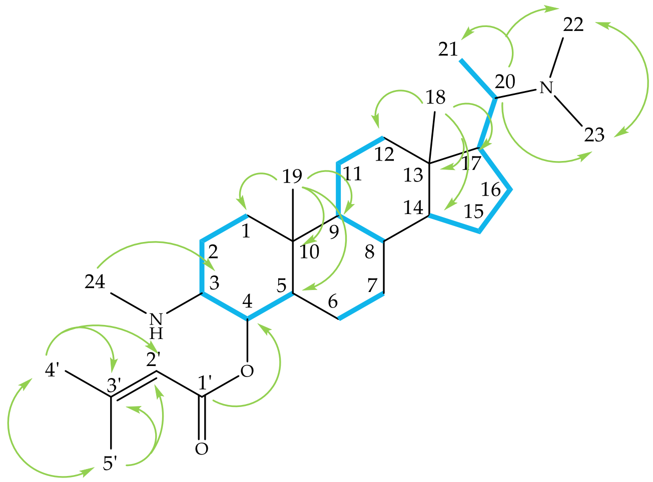

Figure S97. Key COSY (blue lines) and HMBC (green arrows) correlations of pachysandrine D (**10**).

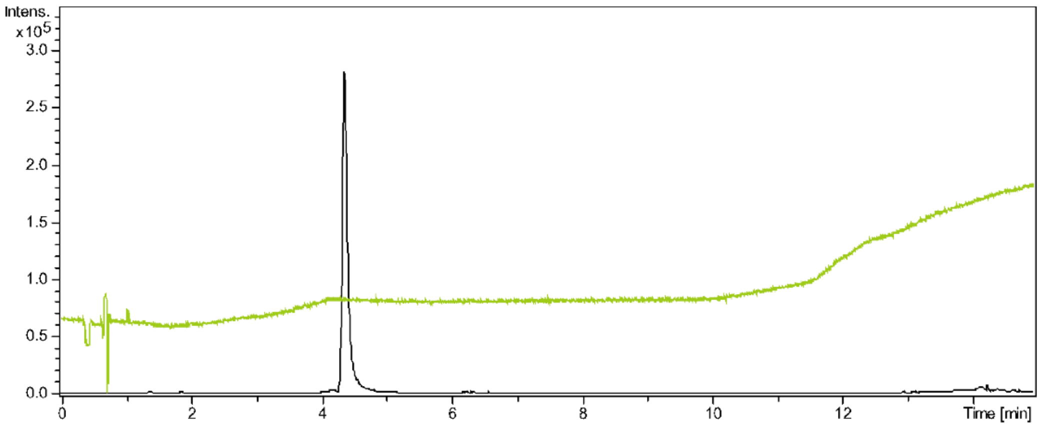

Figure S98. UHPLC/+ESI-QqTOF-MS/MS chromatogram of terminaline (**11**). Base peak chromatogram 200.0000-1000.0000 +All MS (black); UV chromatogram 200-400 nm (green).

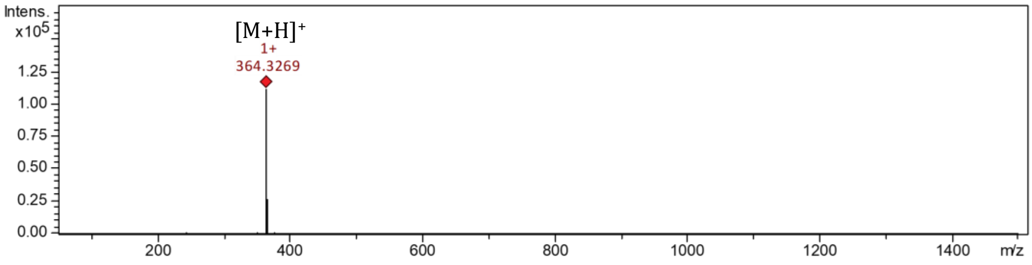

Figure S99. +ESI-QqTOF MS spectrum of terminaline (**11**); *m/z* 364.3269 [M+H]$^+$.

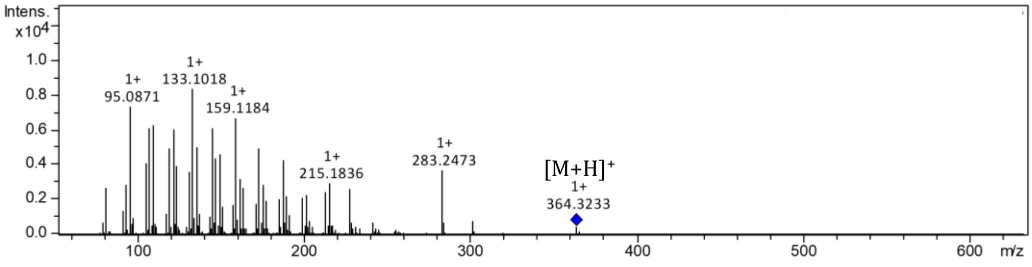

Figure S100. +ESI-QqTOF MS/MS spectrum of terminaline (**11**).

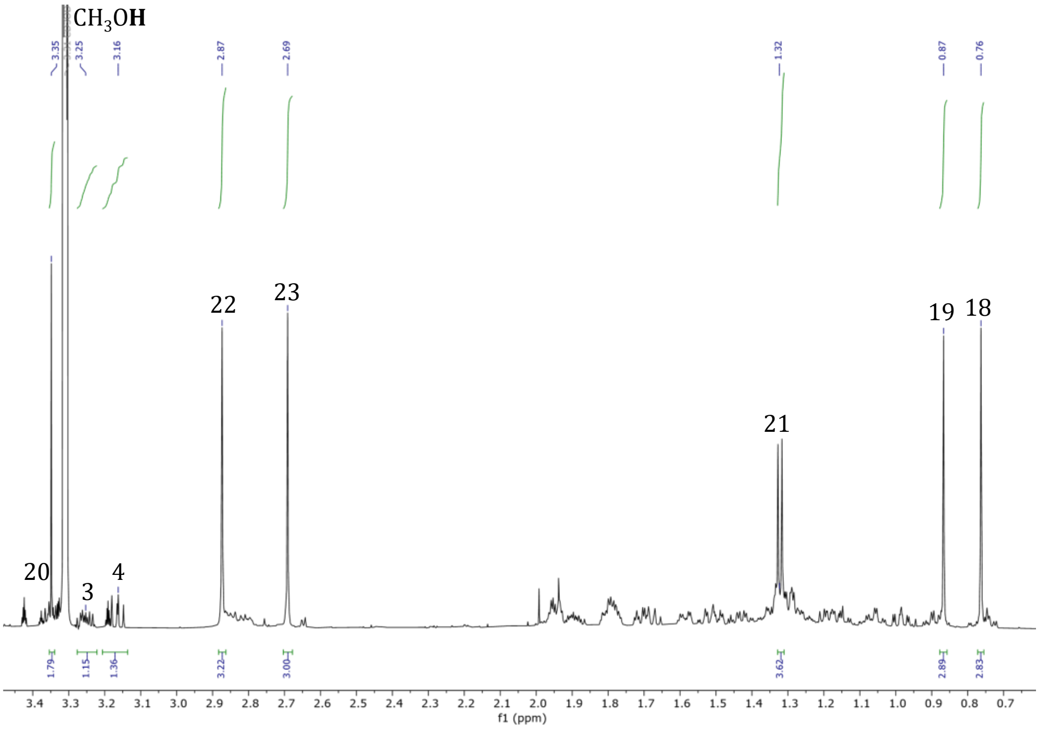

Figure S101. ^{1}H NMR spectrum of terminaline (**11**) (CD_3OD, 600 MHz). The assignment of the signals between 2.05 and 0.65 ppm can be found in the enlarged Figure S102.

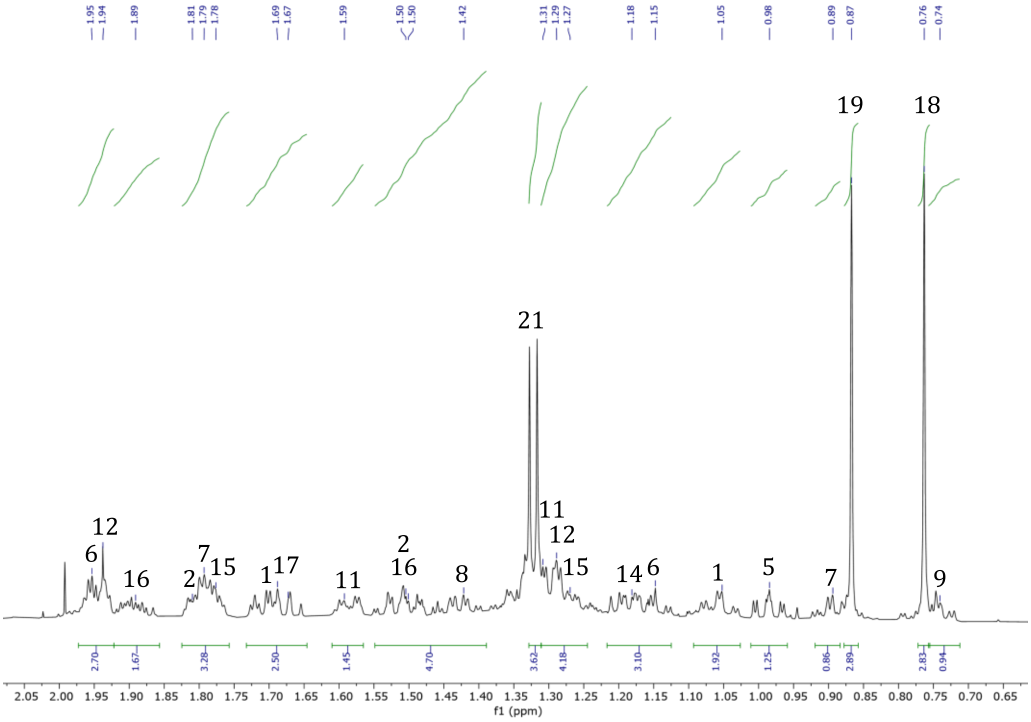

Figure S102. Detail of the ^{1}H NMR spectrum of terminaline (**11**) (CD_3OD, 600 MHz).

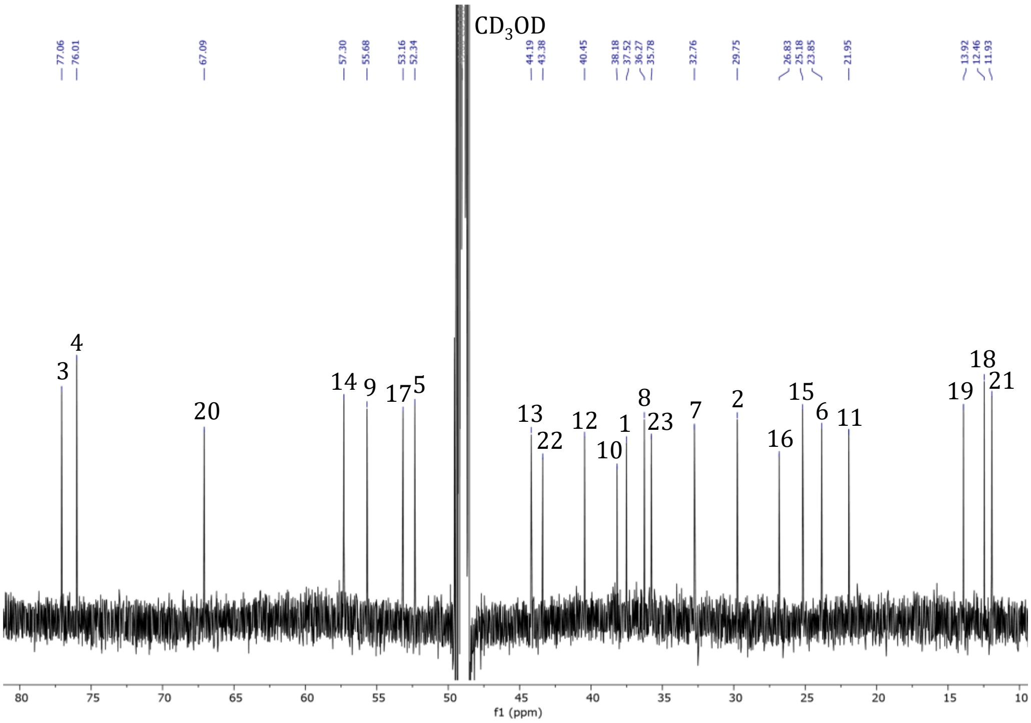

Figure S103. ^{13}C NMR spectrum of terminaline (**11**) (CD_3OD, 151 MHz).

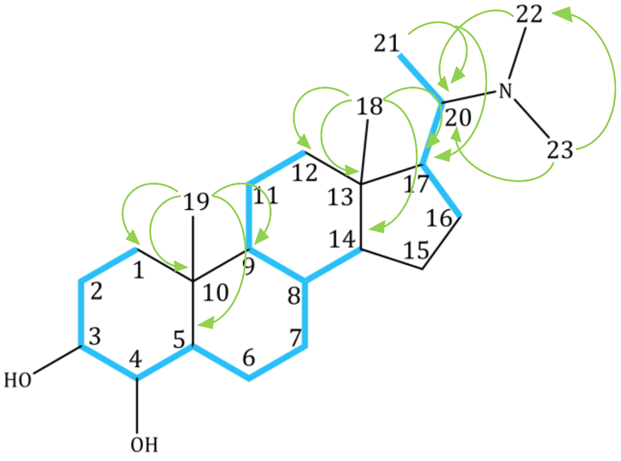

Figure S104. Key COSY (blue lines) and HMBC (green arrows) correlations of terminaline (**11**).

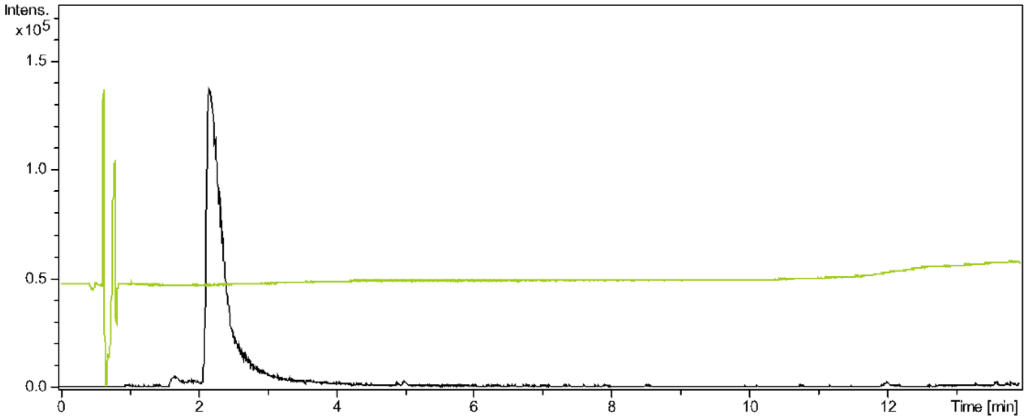

Figure S105. UHPLC/+ESI-QqTOF-MS/MS chromatogram of *N*-methyl-desacyl-epi-pachysamine A (**12**). Base peak chromatogram 200.0000-1000.0000 +All MS (black); UV chromatogram 200-400 nm (green).

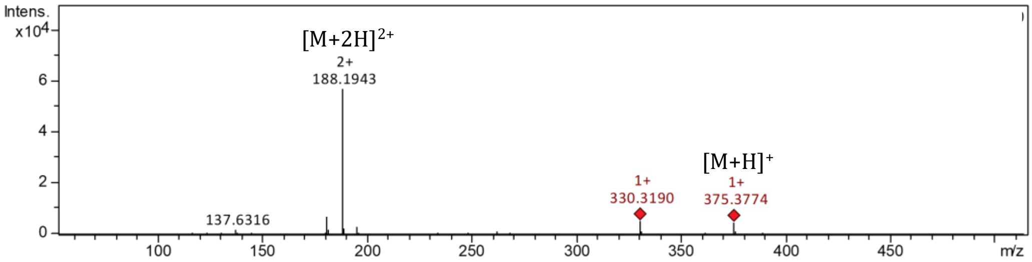

Figure S106. +ESI-QqTOF MS spectrum of *N*-methyl-desacyl-epipachysamine A (**12**); *m*/*z* 375.3774 $[M+H]^+$, *m*/*z* 188.1943 $[M+2H]^{2+}$.

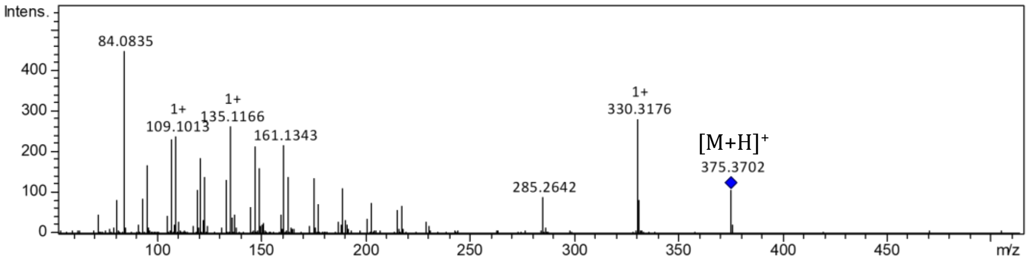

Figure S107. +ESI-QqTOF MS/MS spectrum of *N*-methyl-desacyl-epipachysamine A (**12**).

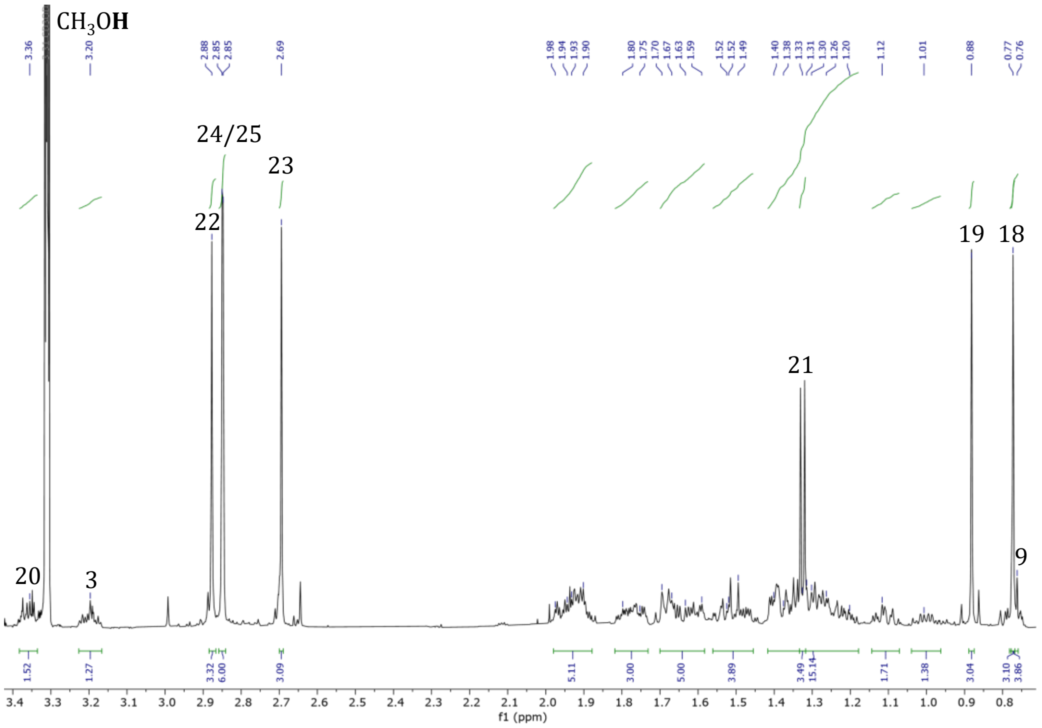

Figure S108. ^{1}H NMR spectrum of *N*-methyl-desacyl-epipachysamine A (**12**) (CD_3OD, 600 MHz). The assignment of the signals between 2.00 and 0.95 ppm can be found in the enlarged Figure S109.

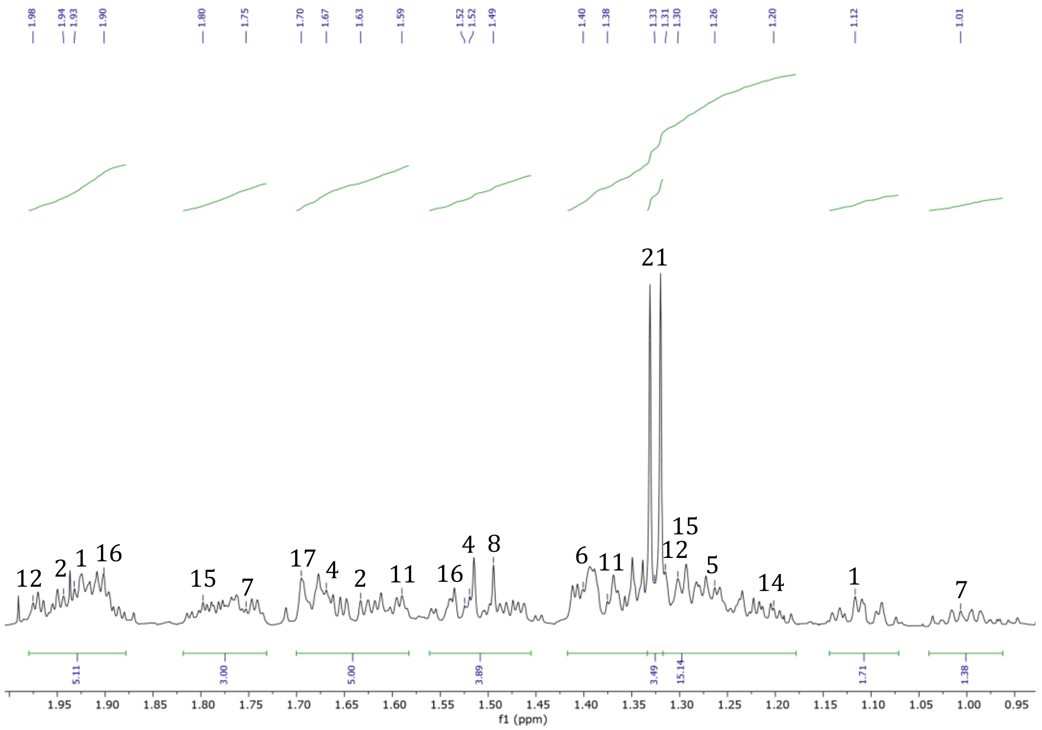

Figure S109. Detail of the ^{1}H NMR spectrum of *N*-methyl-desacyl-epipachysamine A (**12**) (CD_3OD, 600 MHz).

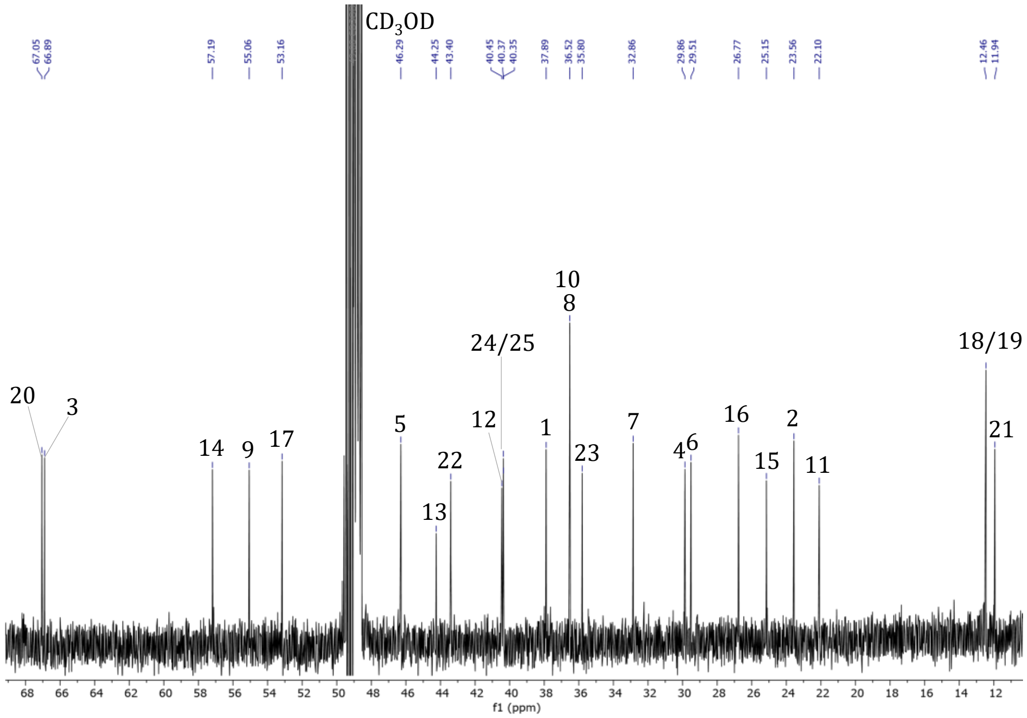

Figure S110. ^{13}C NMR spectrum of *N*-methyl-desacyl-epipachysamine A (**12**) (CD_3OD, 151 MHz).

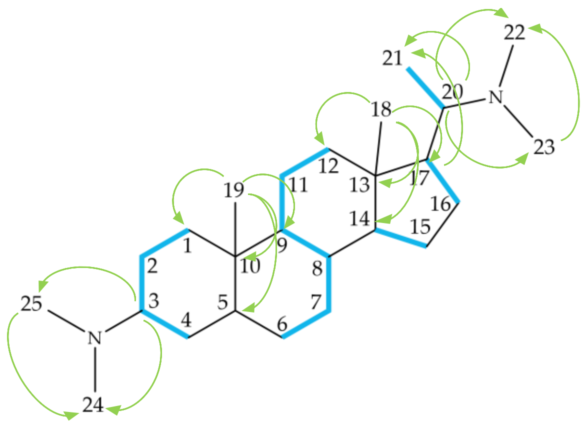

Figure S111. Key COSY (blue lines) and HMBC (green arrows) correlations of *N*-methyl-desacyl-epipachysamine A (**12**).

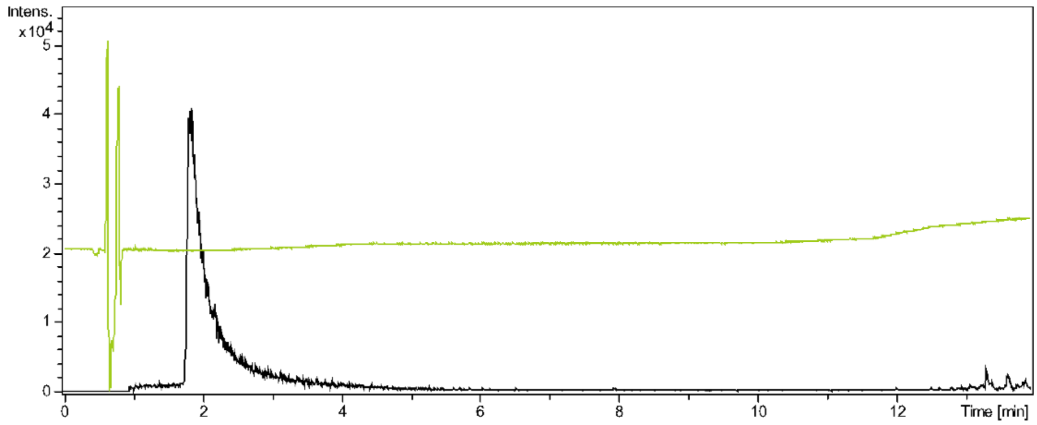

Figure S112. UHPLC/+ESI-QqTOF-MS/MS chromatogram of sarcodinine (**13**). Base peak chromatogram 200.0000-1000.0000 +All MS (black); UV chromatogram 200-400 nm (green).

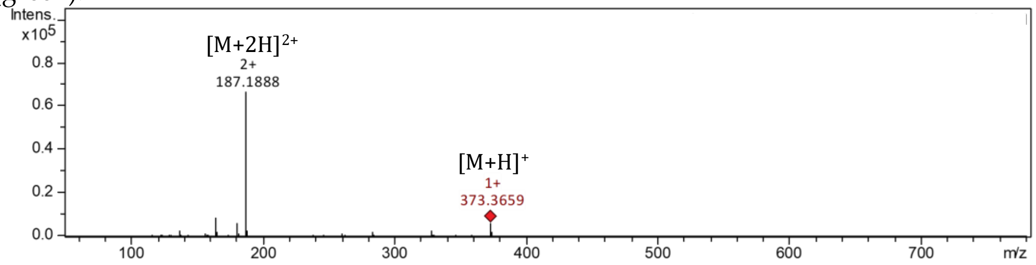

Figure S113. +ESI-QqTOF MS spectrum of sarcodinine (**13**); *m/z* 373.3659 $[M+H]^+$, *m/z* 187.1888 $[M+2H]^{2+}$.

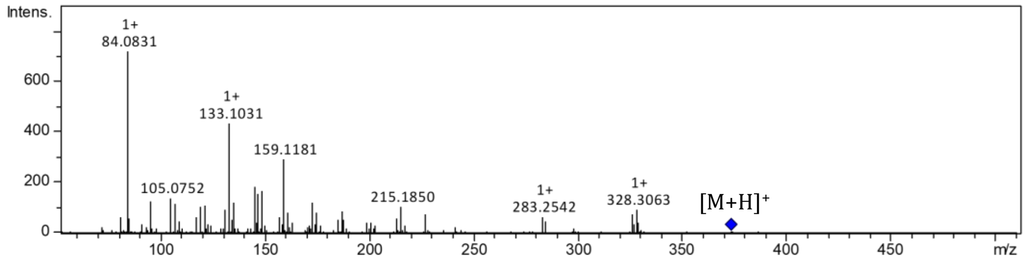

Figure S114. +ESI-QqTOF MS/MS spectrum of sarcodinine (**13**).

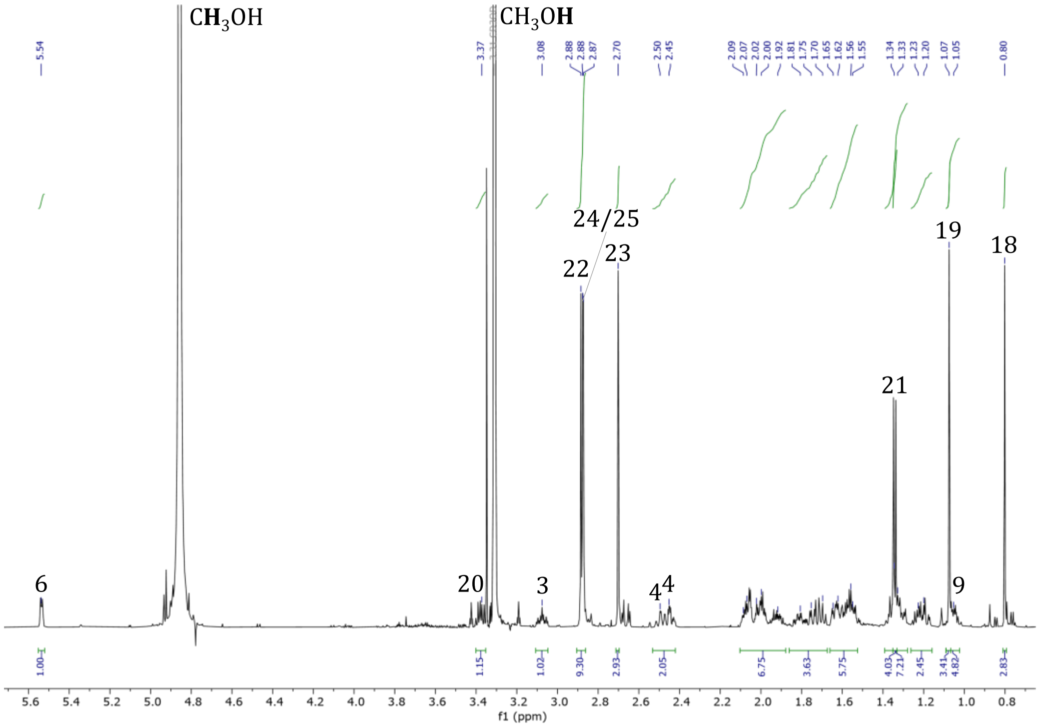

Figure S115. ^{1}H NMR spectrum of sarcodinine (**13**) (CD_3OD, 600 MHz). The assignment of the signals between 2.55 and 0.75 ppm can be found in the enlarged Figure S116.

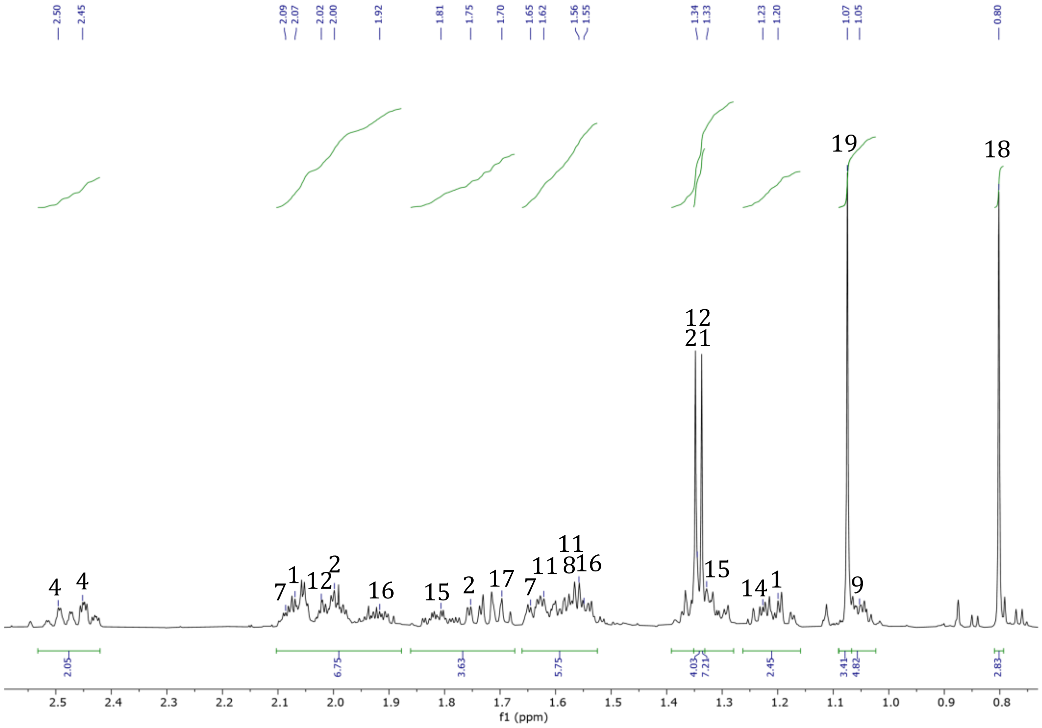

Figure S116. Detail of the ^{1}H NMR spectrum of sarcodinine (**13**) (CD_3OD, 600 MHz).

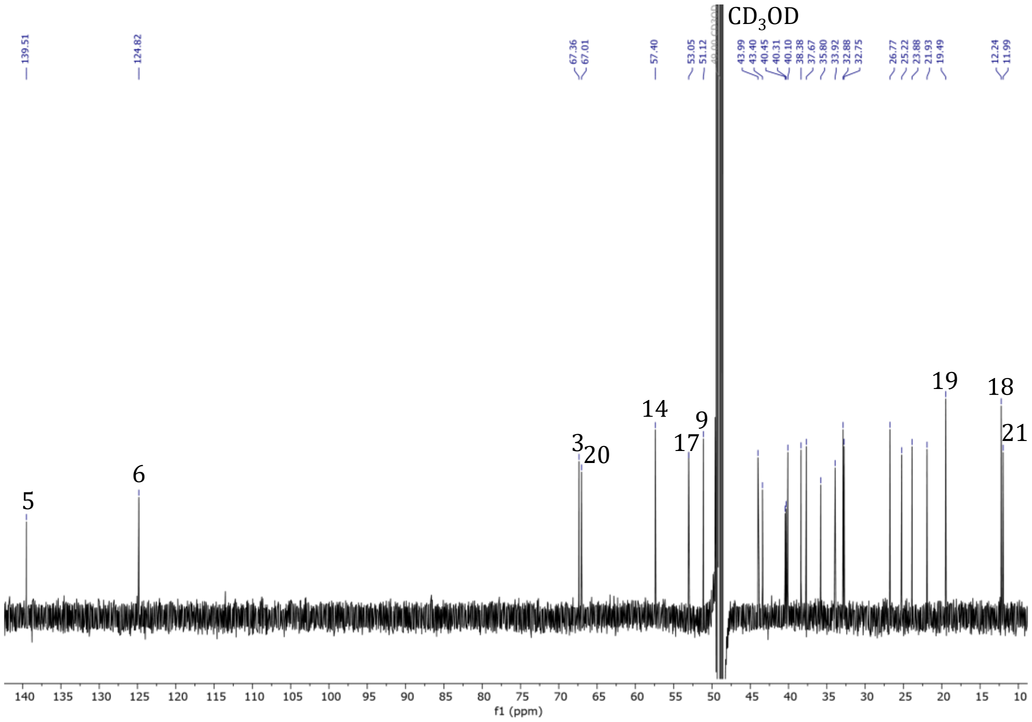

Figure S117. ^{13}C NMR spectrum of sarcodinine (**13**) (CD_3OD, 151 MHz). The assignment of the signals between 70 and 11 ppm can be found in the enlarged Figure S118.

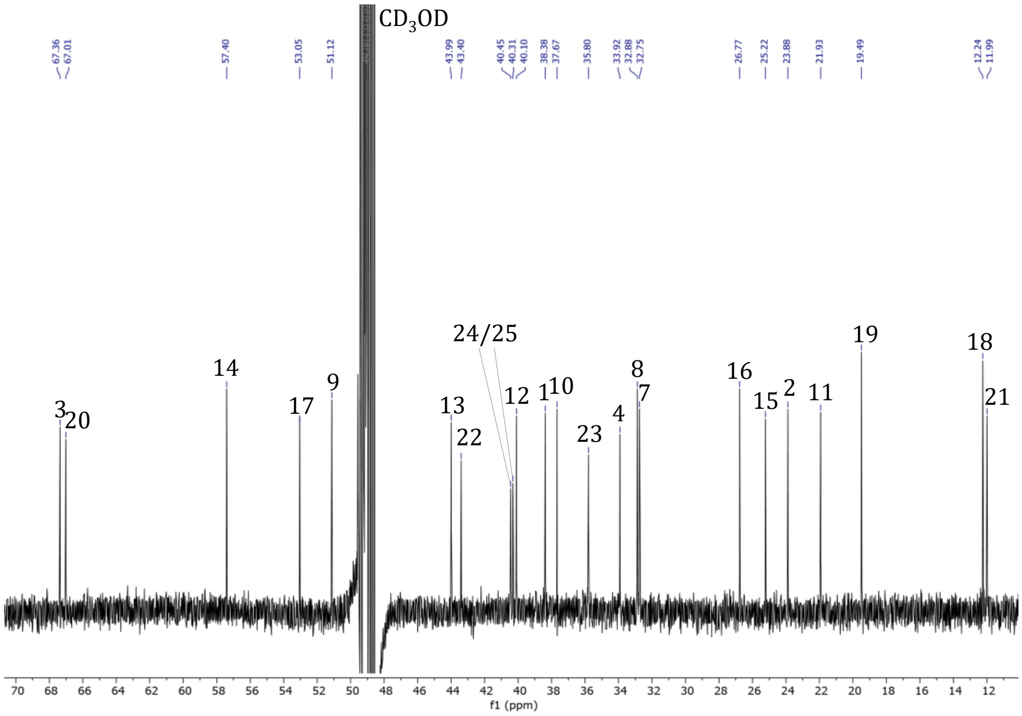

Figure S118. Detail of the ^{13}C NMR spectrum of sarcodinine (**13**) (CD_3OD, 151 MHz).

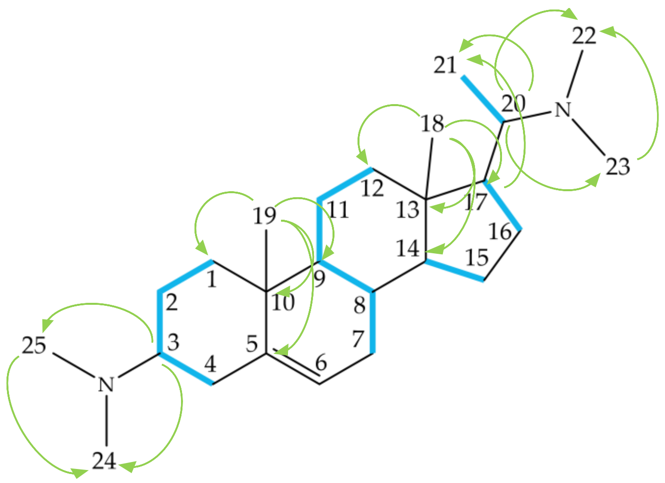

Figure S119. Key COSY (blue lines) and HMBC (green arrows) correlations of sarcodinine (**13**).

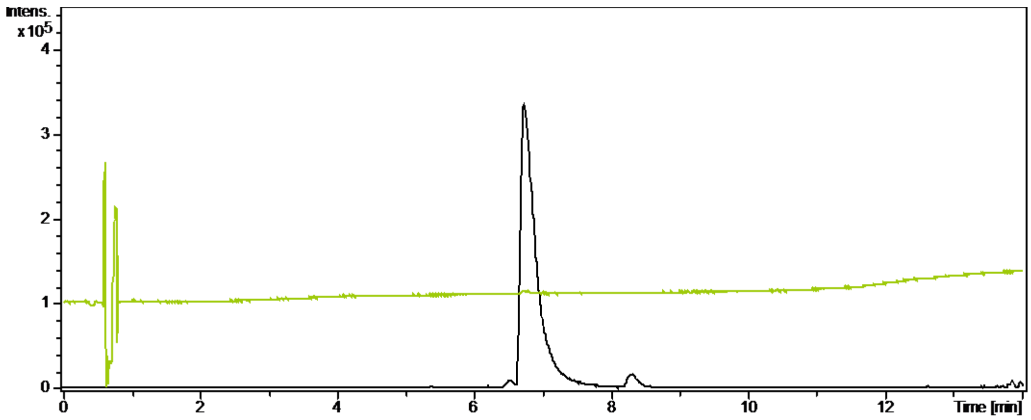

Figure S120. UHPLC/+ESI-QqTOF-MS/MS chromatogram of epipachysamine A (**14**). Base peak chromatogram 200.0000-1000.0000 +All MS (black); UV chromatogram 200-400 nm (green).

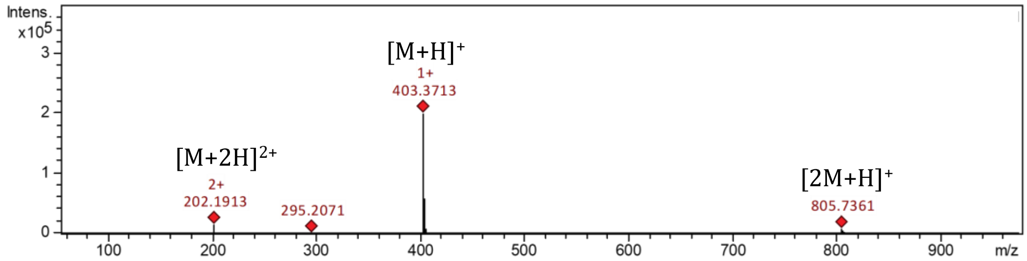

Figure S121. +ESI-QqTOF MS spectrum of epipachysamine A (**14**); *m/z* 403.3713 $[M+H]^+$, *m/z* 202.1913 $[M+2H]^{2+}$.

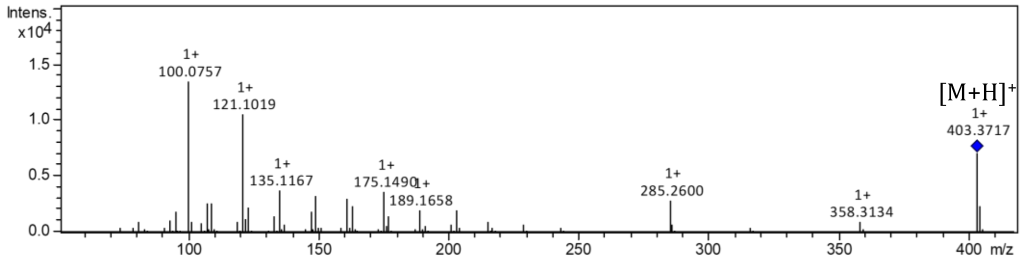

Figure S122. +ESI-QqTOF MS/MS spectrum of epipachysamine A (**14**).

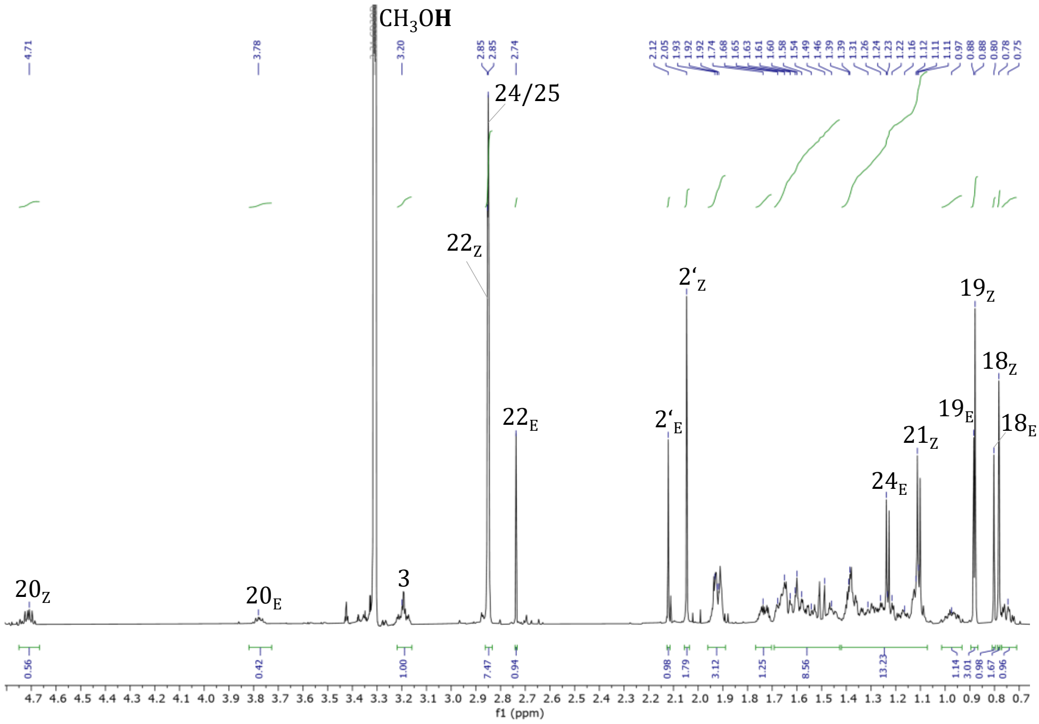

Figure S123. ^{1}H NMR spectrum of epipachysamine A (**14**) (CD_3OD, 600 MHz). The assignment of the signals between 2.15 and 0.70 ppm can be found in the enlarged Figure S124.

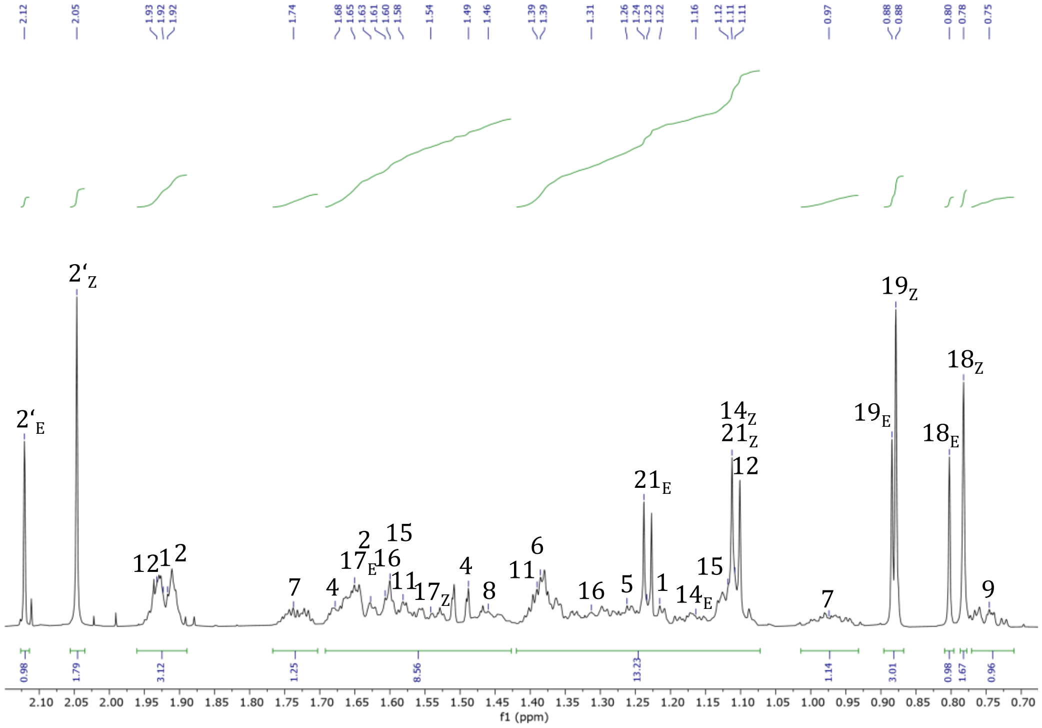

Figure S124. Detail of the ^{1}H NMR spectrum of epipachysamine A (**14**) (CD_3OD, 600 MHz).

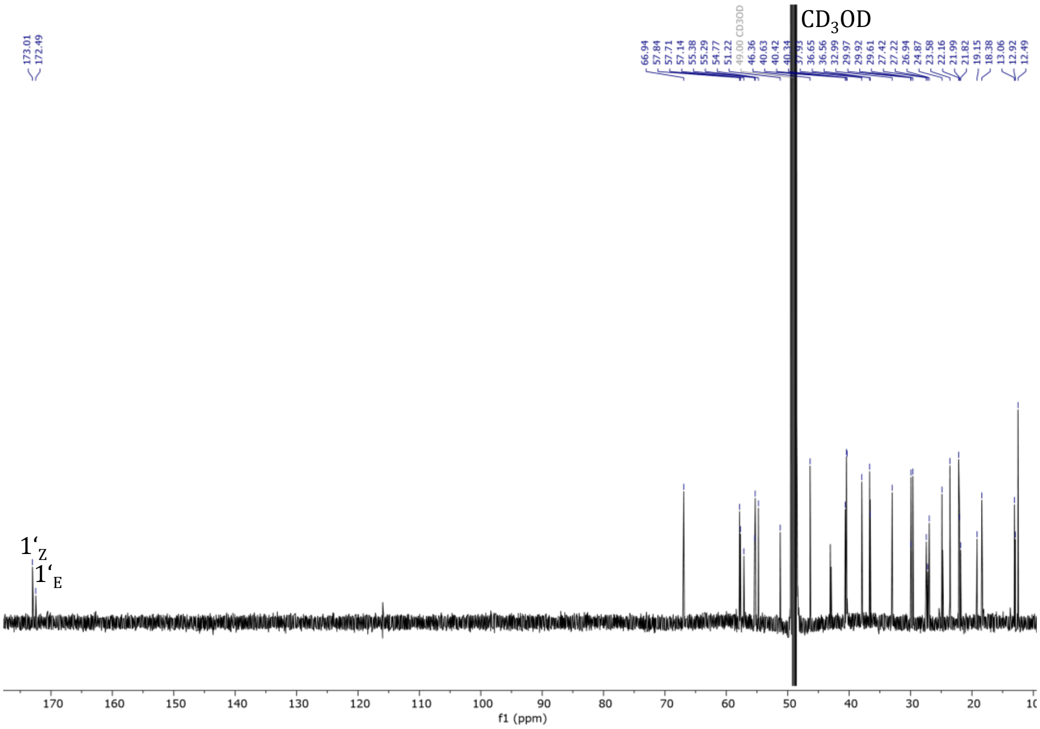

Figure S125. ^{13}C NMR spectrum of epipachysamine A (**14**) (CD$_3$OD, 151 MHz). The assignment of the signals between 71 and 9 ppm can be found in the enlarged Figure S126.

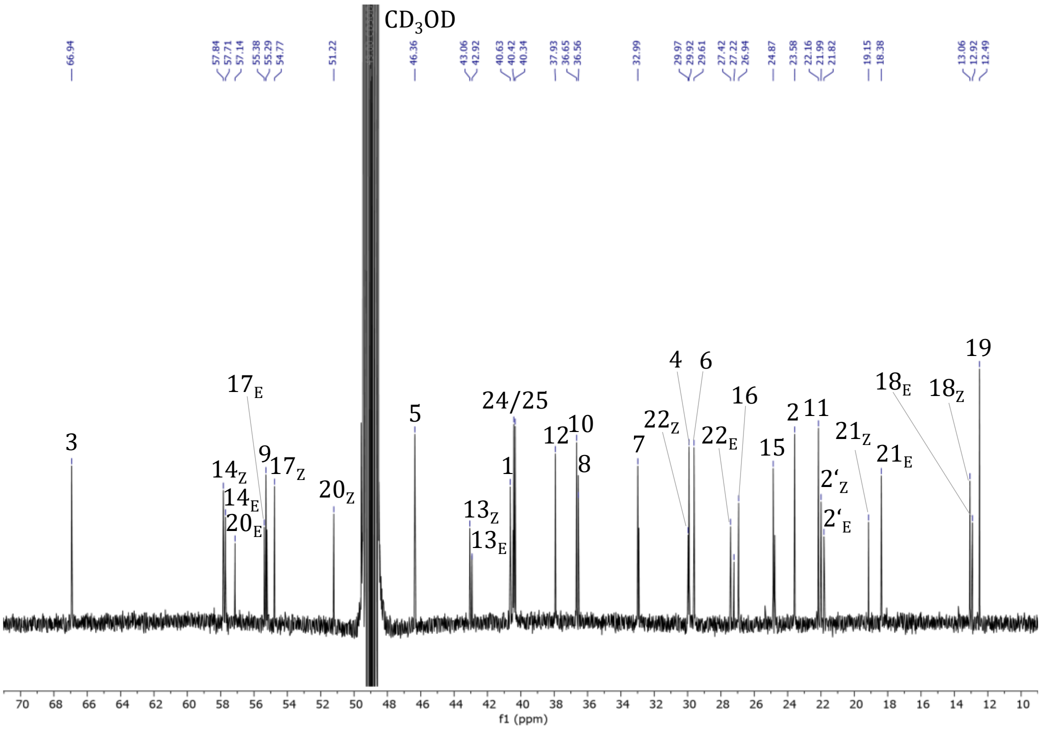

Figure S126. Detail of the ^{13}C NMR spectrum of epipachysamine A (**14**) (CD$_3$OD, 151 MHz).

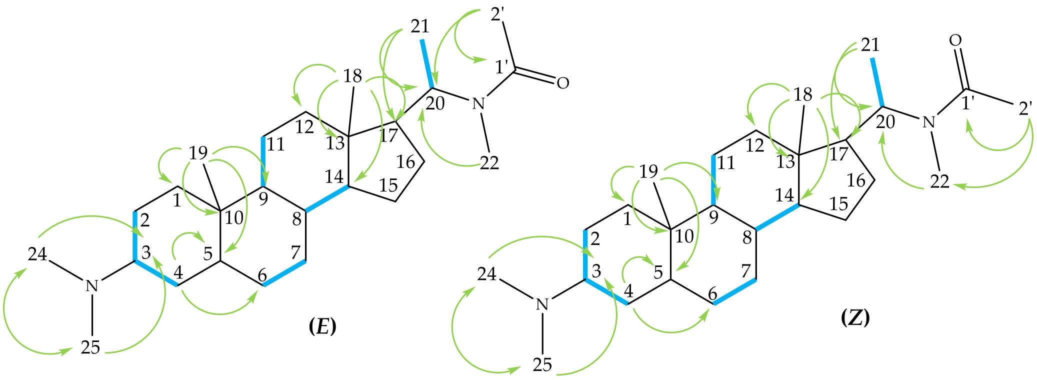

Figure S127. Key COSY (blue lines) and HMBC (green arrows) correlations of *E*- and *Z*-epipachysamine A (**14**).

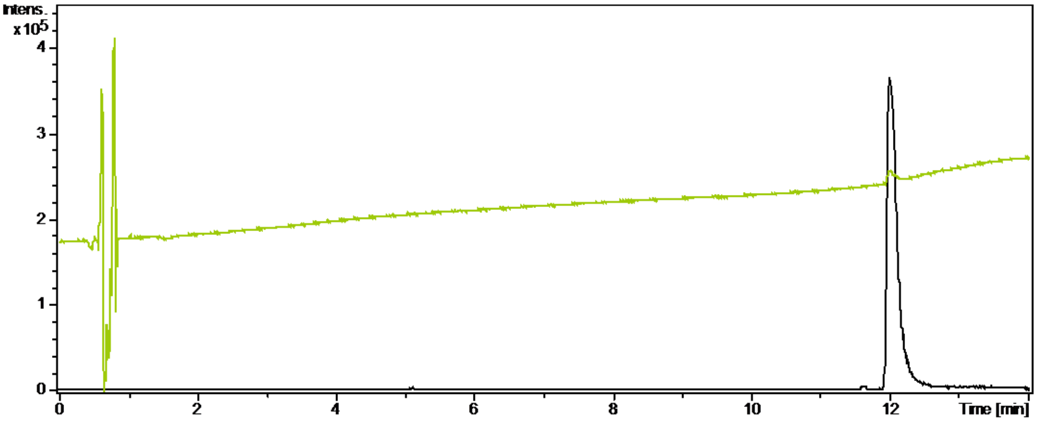

Figure S128. UHPLC/+ESI-QqTOF-MS/MS chromatogram of spiropachysine (**15**). Base peak chromatogram 200.0000-1000.0000 +All MS (black); UV chromatogram 200-400 nm (green).

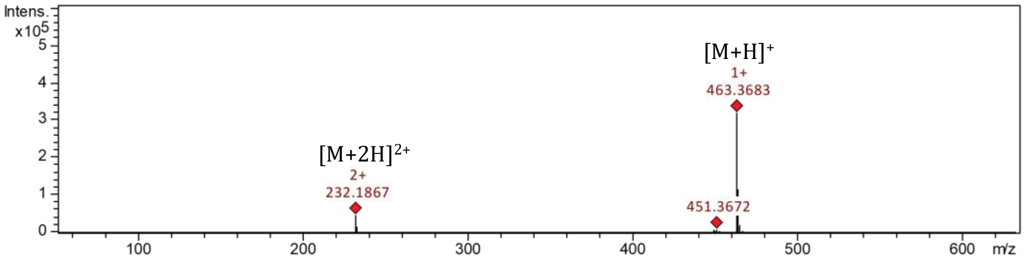

Figure S129. +ESI-QqTOF MS spectrum of spiropachysine (**15**); *m/z* 463.3683 $[M+H]^+$, *m/z* 232.1867 $[M+2H]^{2+}$.

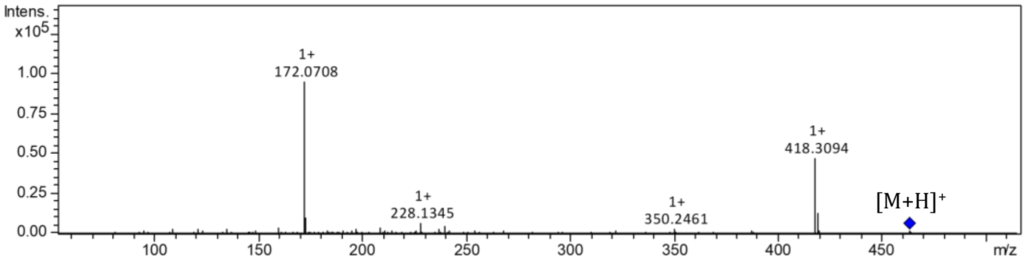

Figure S130. +ESI-QqTOF MS/MS spectrum of spiropachysine (**15**).

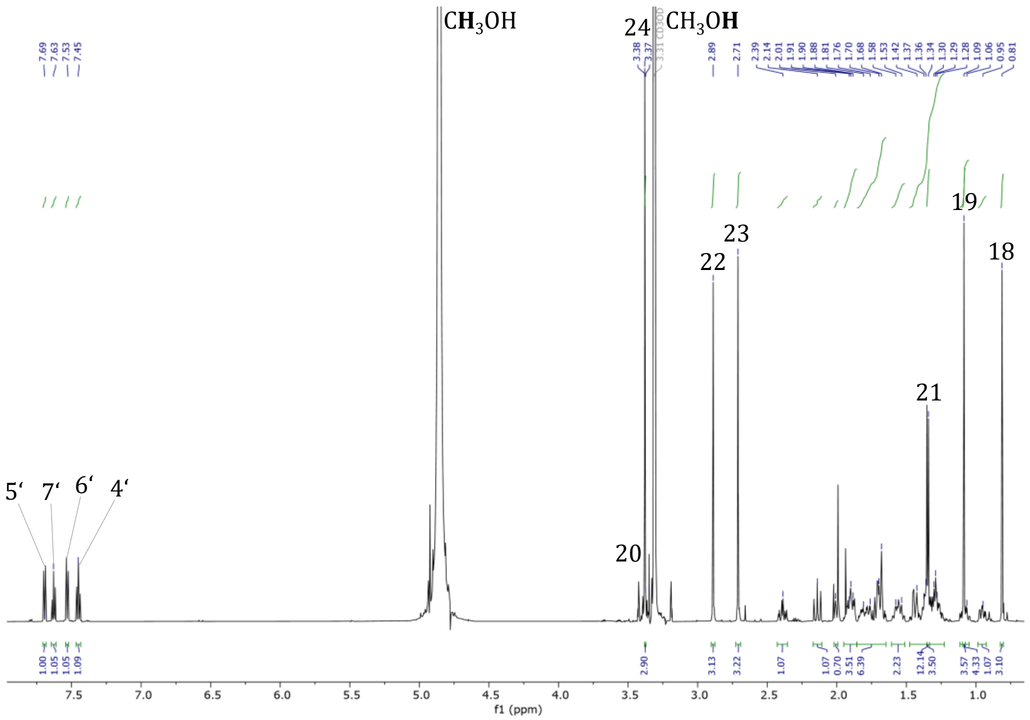

Figure S131. ^{1}H NMR spectrum of spiropachysine (**15**) (CD_3OD, 600 MHz). The assignment of the signals between 2.95 and 0.75 ppm can be found in the enlarged Figure S132.

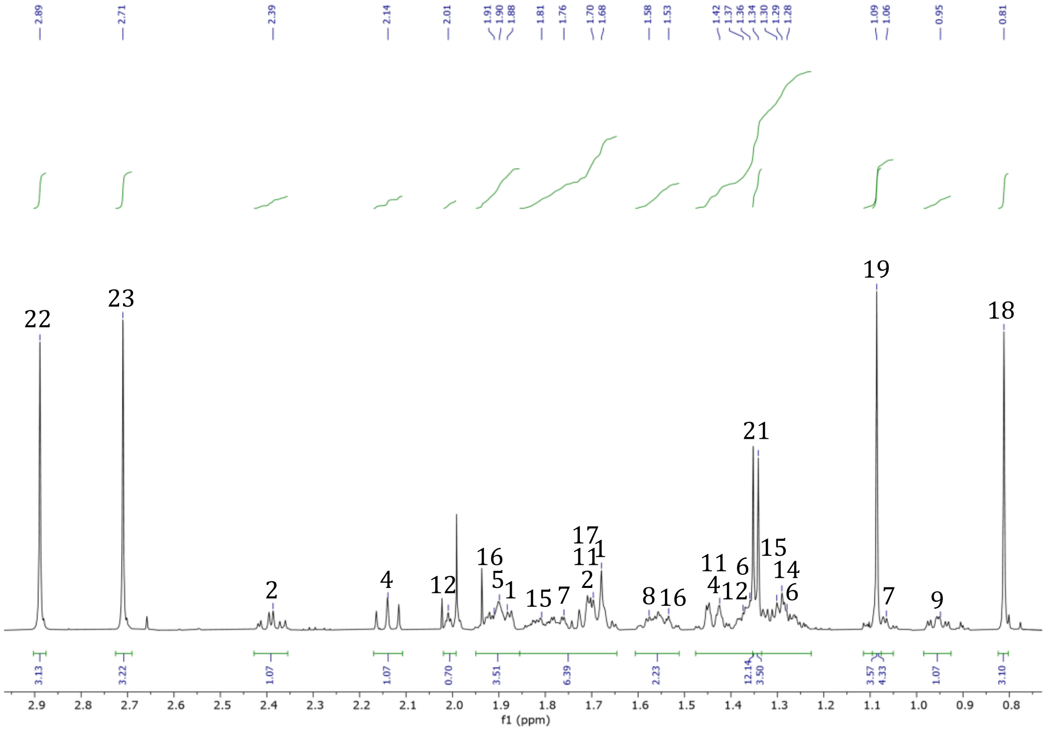

Figure S132. Detail of the ^{1}H NMR spectrum of spiropachysine (**15**) (CD_3OD, 600 MHz).

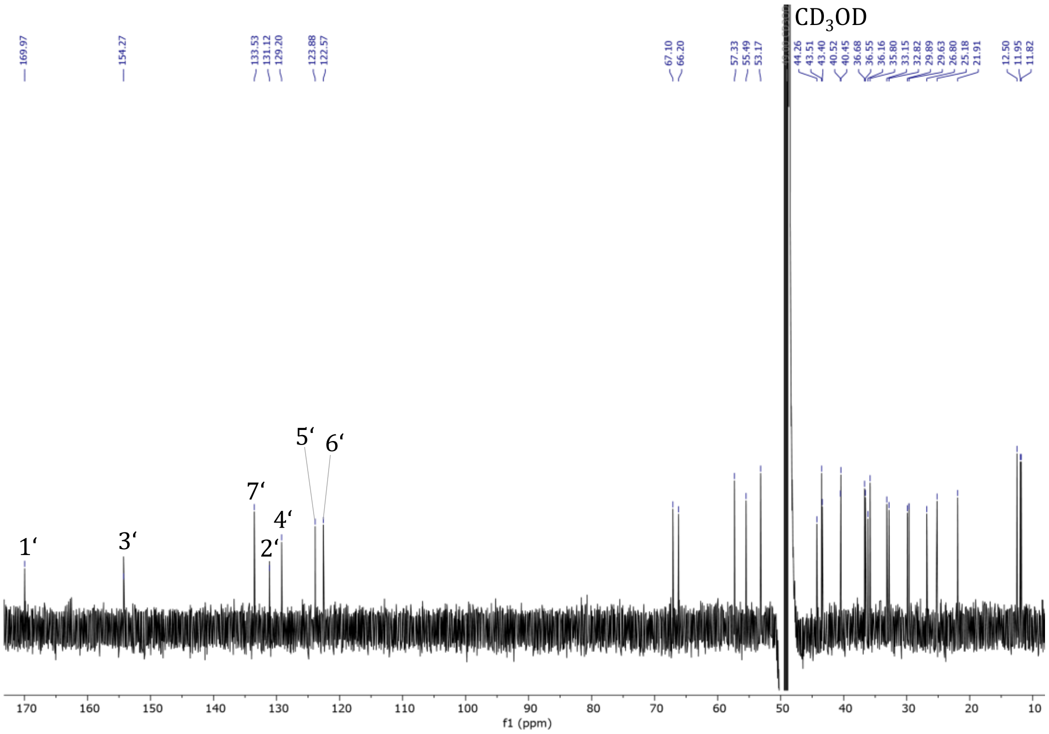

Figure S133. ^{13}C NMR spectrum of spiropachysine (**15**) (CD_3OD, 151 MHz). The assignment of the signals between 70 and 10 ppm can be found in the enlarged Figure S134.

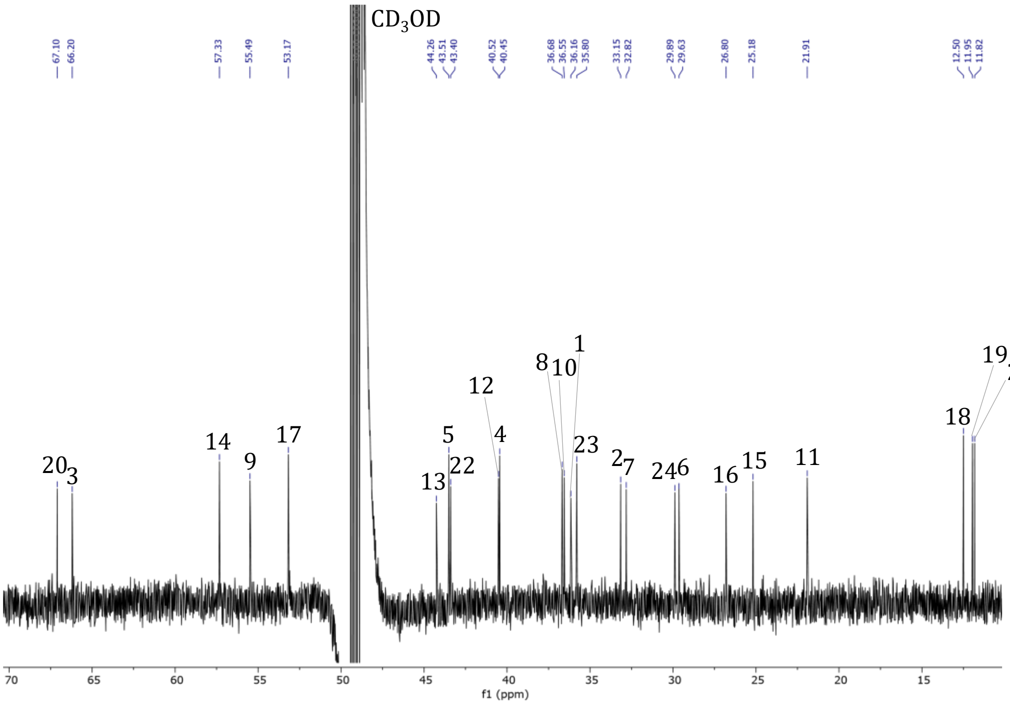

Figure S134. Detail of the ^{13}C NMR spectrum of spiropachysine (**15**) (CD_3OD, 151 MHz).

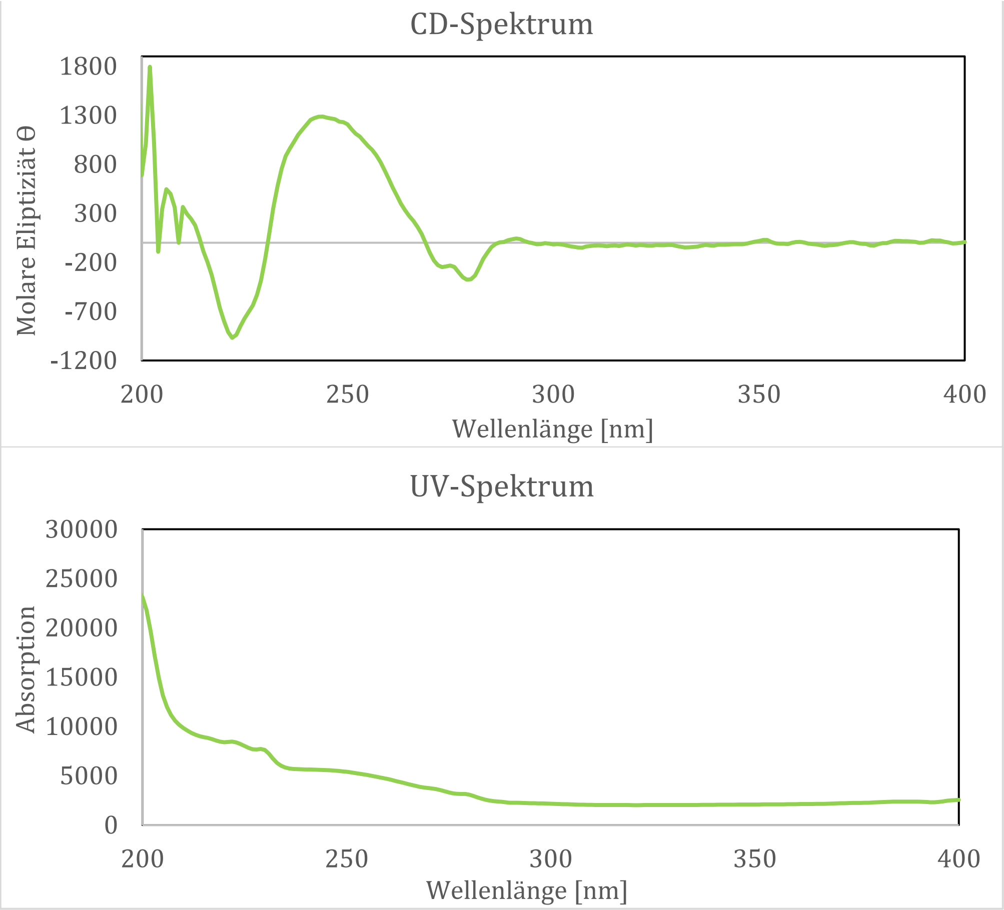

Figure S135. CD and UV spectrum of spiropachysine (**15**) in MeOH (1 mg/mL).

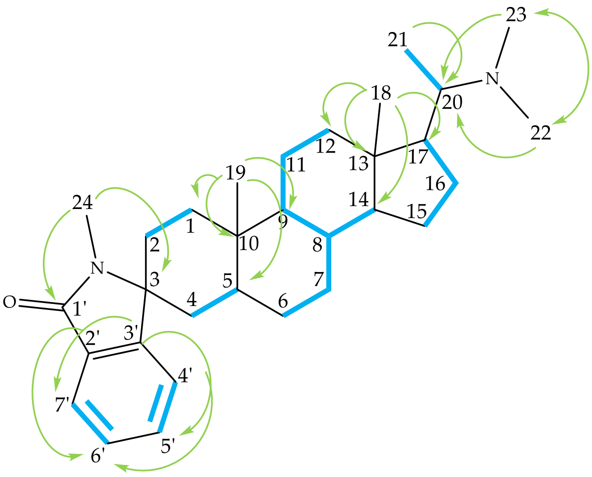

Figure S136. Key COSY (blue lines) and HMBC (green arrows) correlations of spiropachysine (**15**).

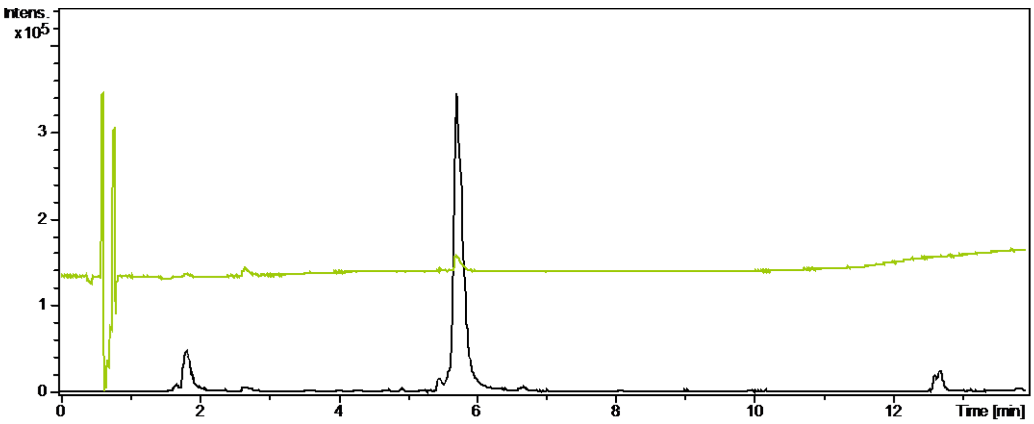

Figure S137. UHPLC/+ESI-QqTOF-MS/MS chromatogram of 4β-hydroxy-hookerianamide N (**16**). Base peak chromatogram 200.0000-1000.0000 +All MS (black); UV chromatogram 200-400 nm (green).

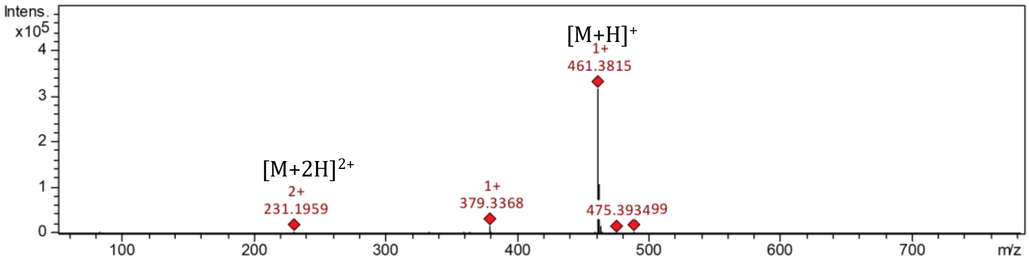

Figure S138. +ESI-QqTOF MS spectrum of 4β-hydroxy-hookerianamide N (**16**); *m/z* 461.3815 $[M+H]^+$, *m/z* 231.1959 $[M+2H]^{2+}$.

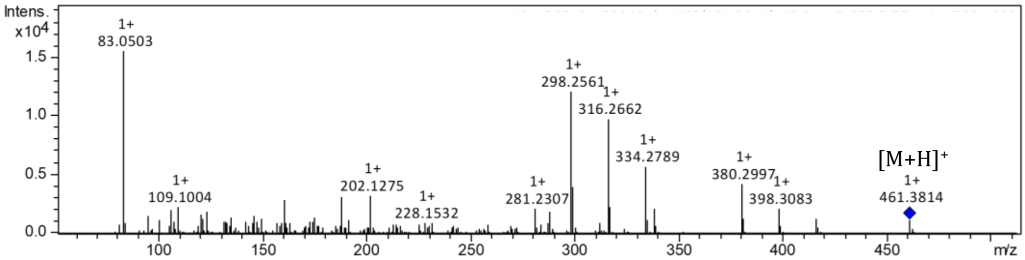

Figure S139. +ESI-QqTOF MS/MS spectrum of 4β-hydroxy-hookerianamide N (**16**).

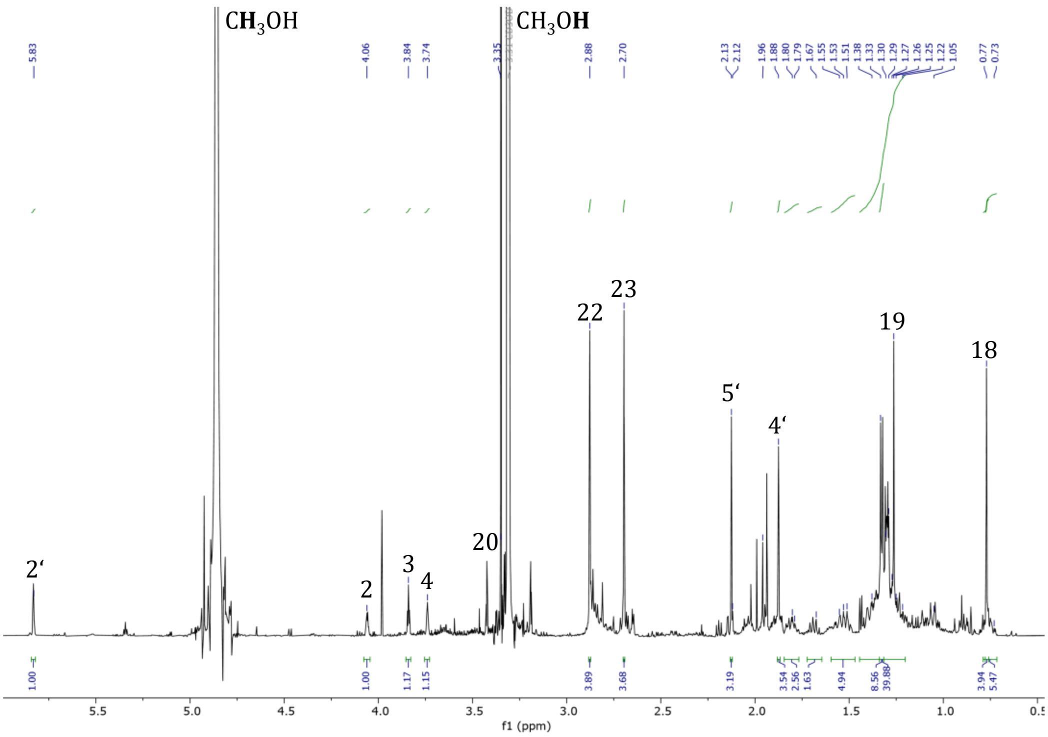

Figure S140. ^{1}H NMR spectrum of 4β-hydroxy-hookerianamide N (**16**) (CD_3OD, 600 MHz). The assignment of the signals between 2.22 and 0.70 ppm can be found in the enlarged Figure S141.

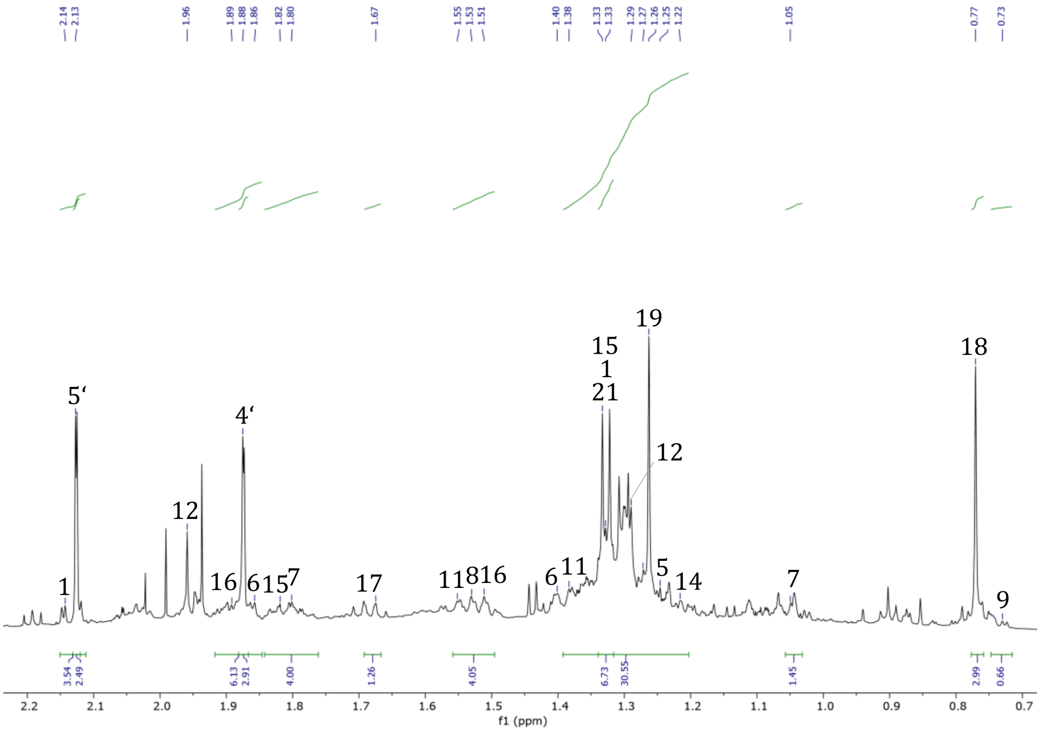

Figure S141. Detail of the ^{1}H NMR spectrum of 4β-hydroxy-hookerianamide N (**16**) (CD_3OD, 600 MHz).

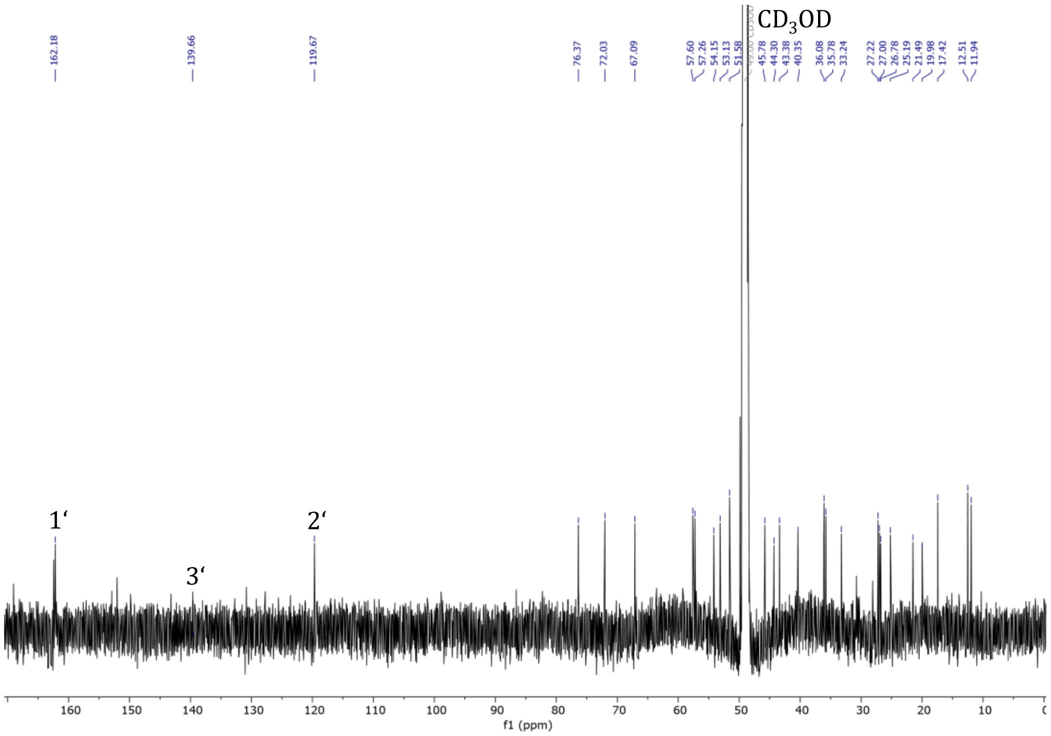

Figure S142. ^{13}C NMR spectrum of 4β-hydroxy-hookerianamide N (**16**) (CD_3OD, 151 MHz). The assignment of the signals between 80 and 10 ppm can be found in the enlarged Figure S143.

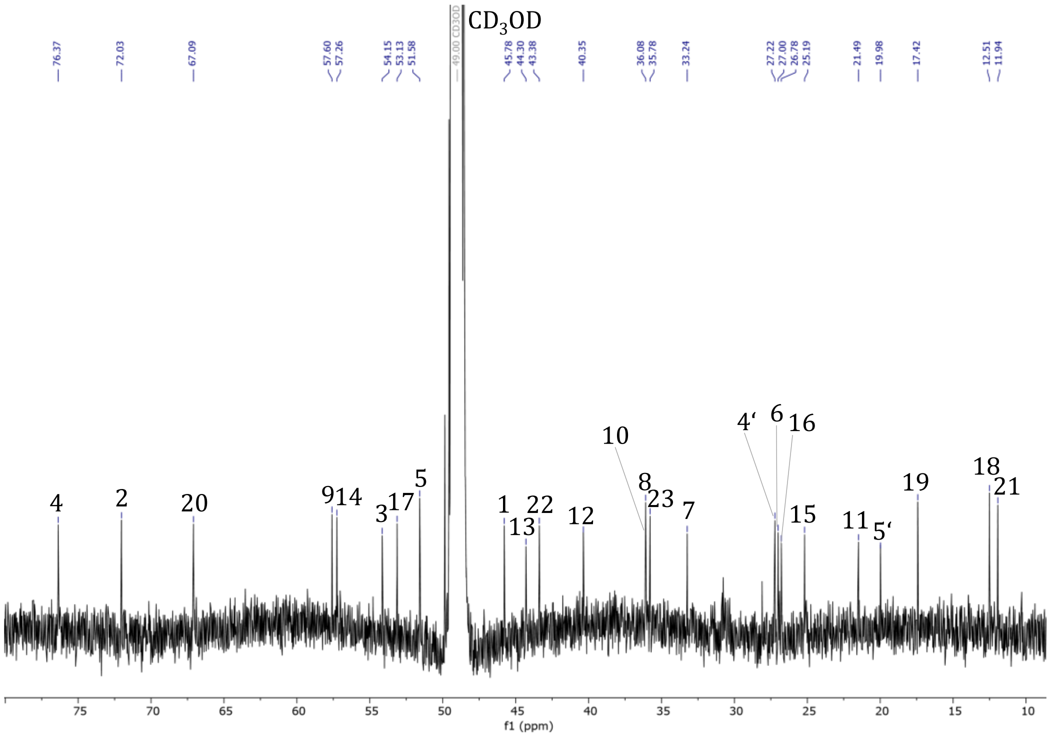

Figure S143. Detail of the ^{13}C NMR spectrum of 4β-hydroxy-hookerianamide N (**16**) (CD_3OD, 151 MHz).

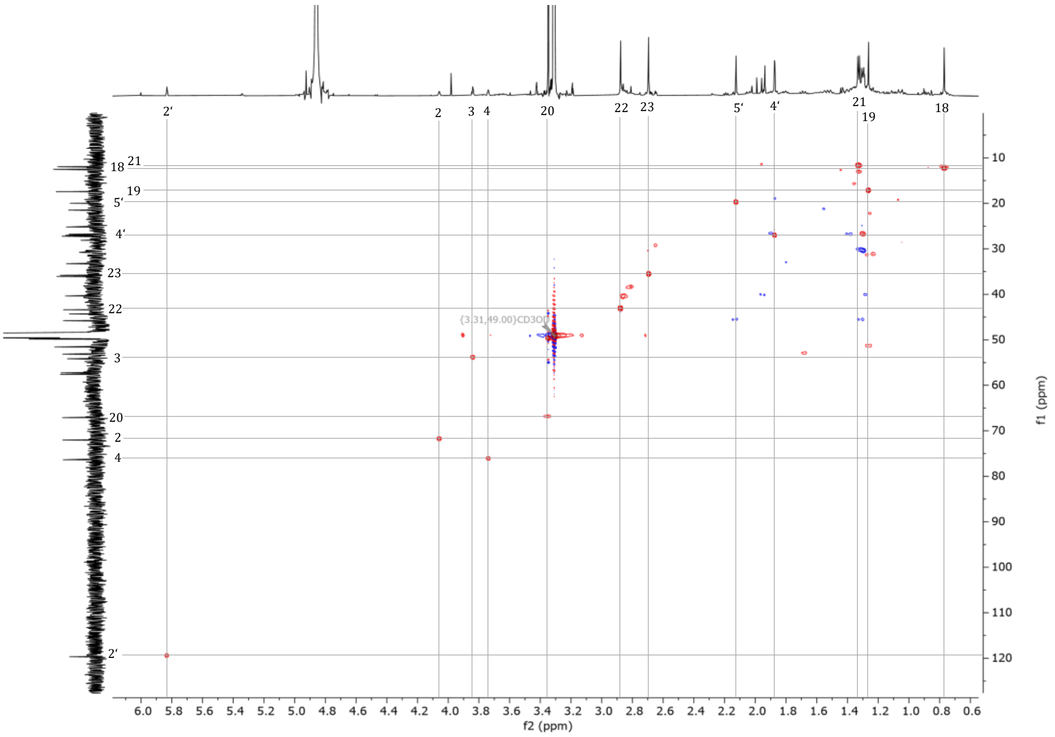

Figure S144. $^1H/^{13}C$ HSQC spectrum of 4β-hydroxy-hookerianamide N (**16**) (CD_3OD, 600/151 MHz).

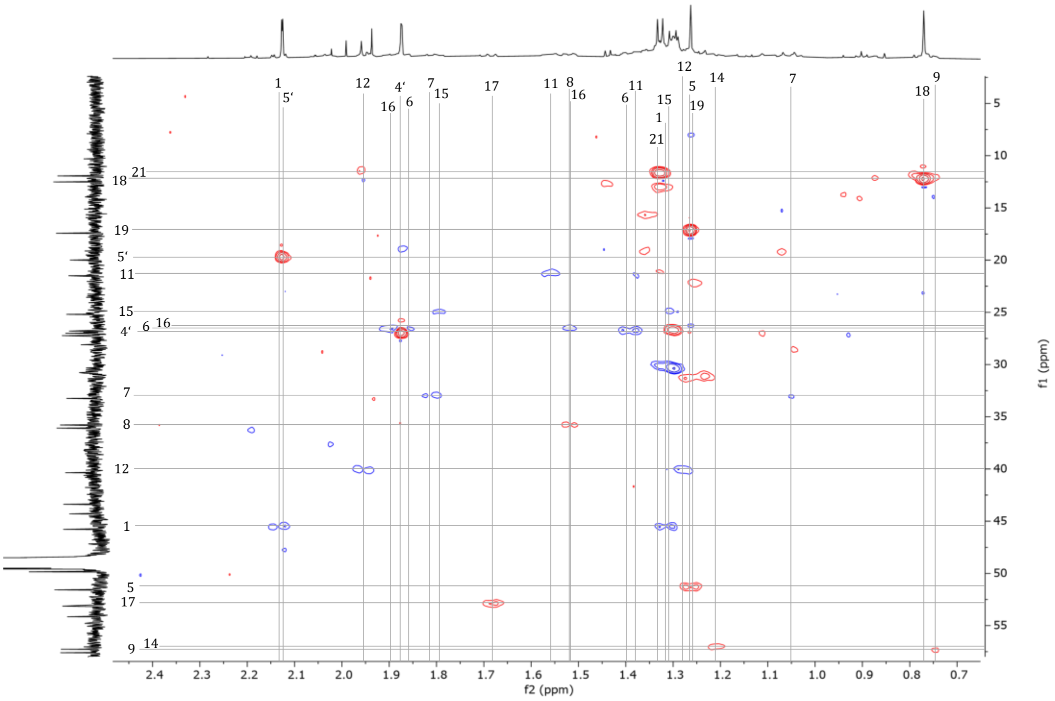

Figure S145. Detail of the $^1H/^{13}C$ HSQC spectrum of 4β-hydroxy-hookerianamide N (**16**) (CD_3OD, 600/151 MHz).

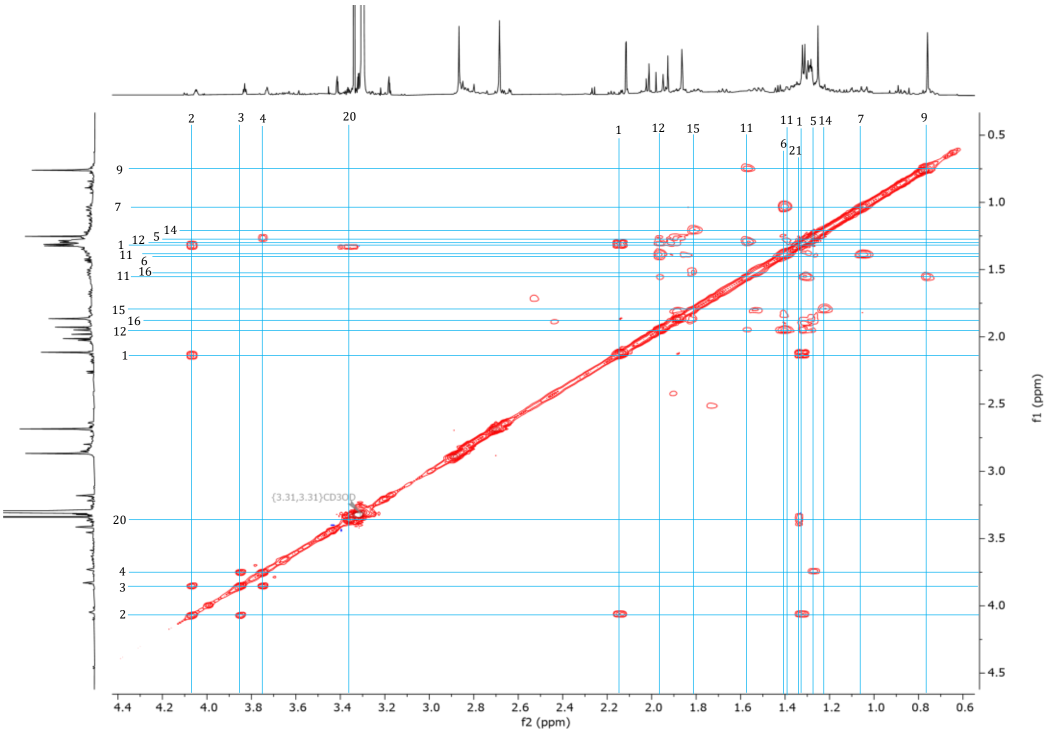

Figure S146. $^1H/^1H$ COSY spectrum of 4β-hydroxy-hookerianamide N (**16**) (CD_3OD, 600 MHz).

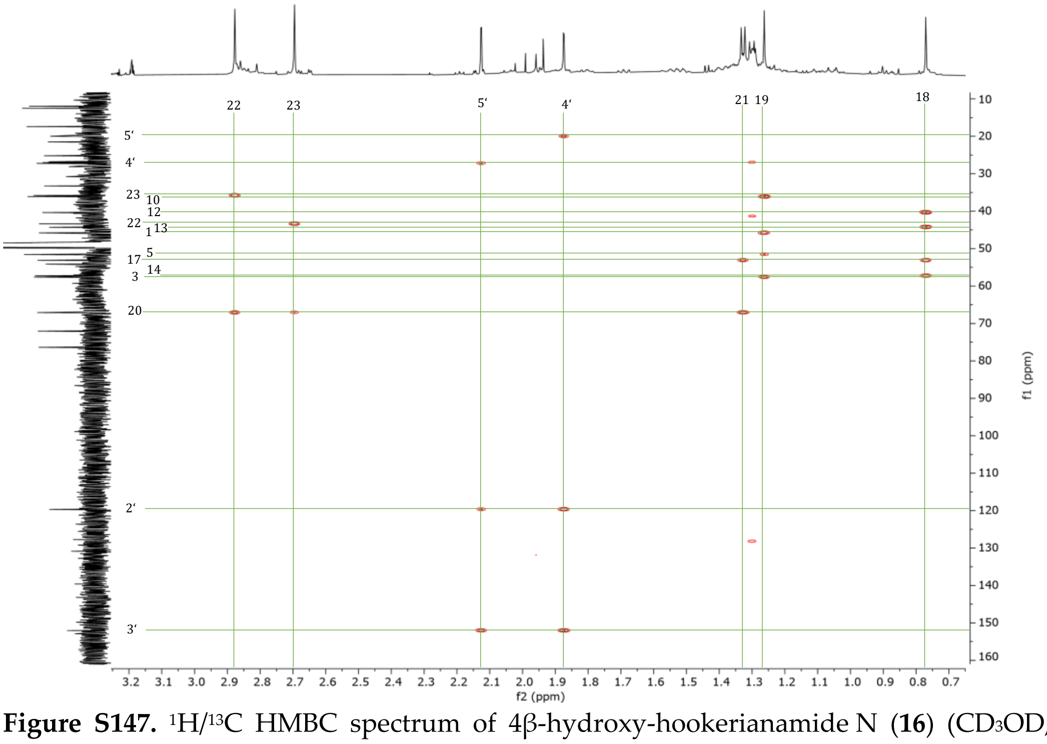

Figure S147. $^1H/^{13}C$ HMBC spectrum of 4β-hydroxy-hookerianamide N (**16**) (CD_3OD, 600/151 MHz).

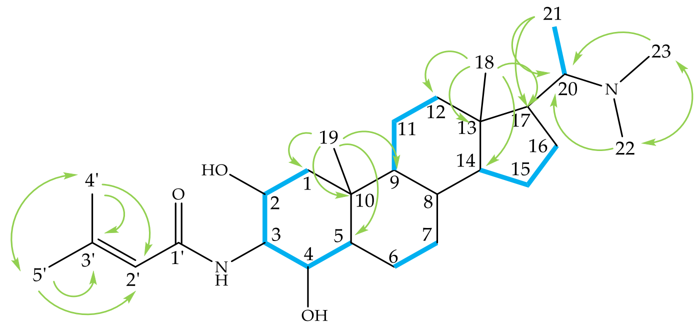

Figure S148. Key COSY (blue lines) and HMBC (green arrows) correlations of 4β-hydroxy-hookerianamide N (**16**).

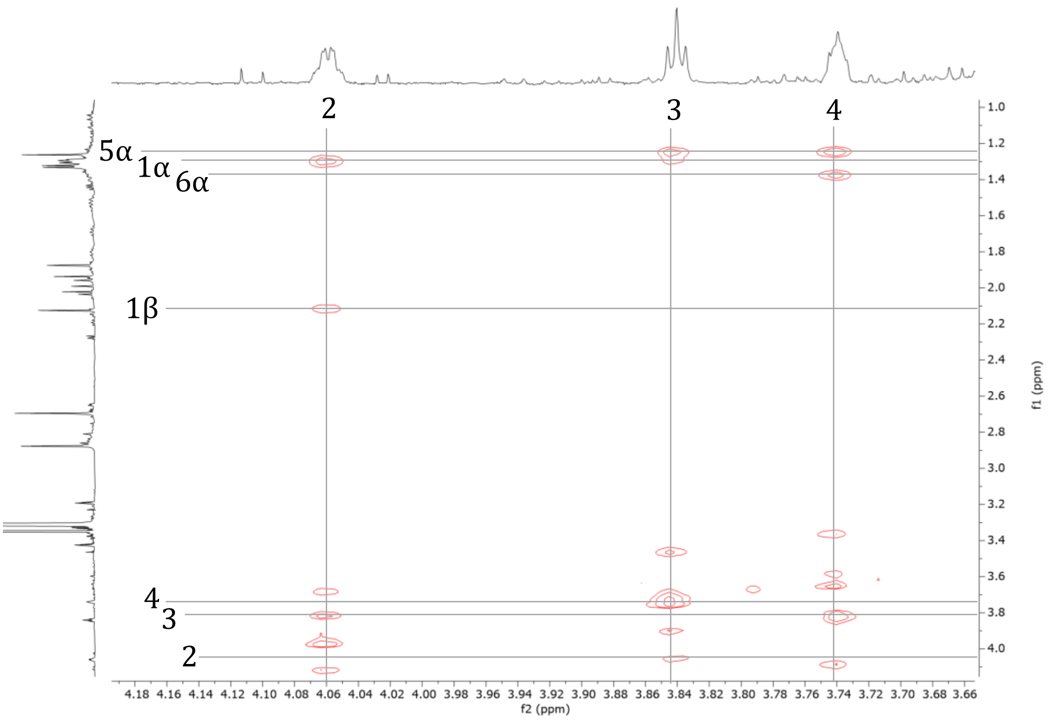

Figure S149. Detail of the ^{1}H/^{1}H NOESY spectrum of 4β-hydroxy-hookerianamide N (**16**) (CD_3OD, 600 MHz).

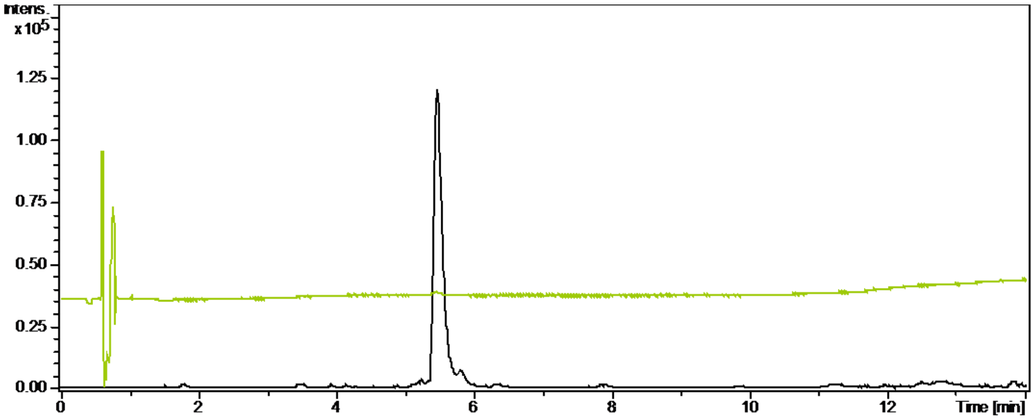

Figure S150. UHPLC/+ESI-QqTOF-MS/MS chromatogram of 5*α*-hydroxy-3*α*,4*α*-diapachysanaximine A (**17**). Base peak chromatogram 200.0000-1000.0000 +All MS (black); UV chromatogram 200-400 nm (green).

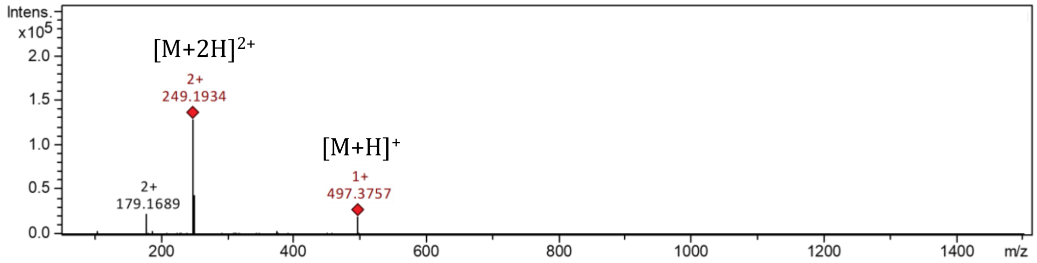

Figure S151. +ESI-QqTOF MS spectrum of 5α-hydroxy-3α,4α-diapachysanaximine A (**17**); *m/z* 497.3757 $[M+H]^+$, *m/z* 249.1934 $[M+2H]^{2+}$.

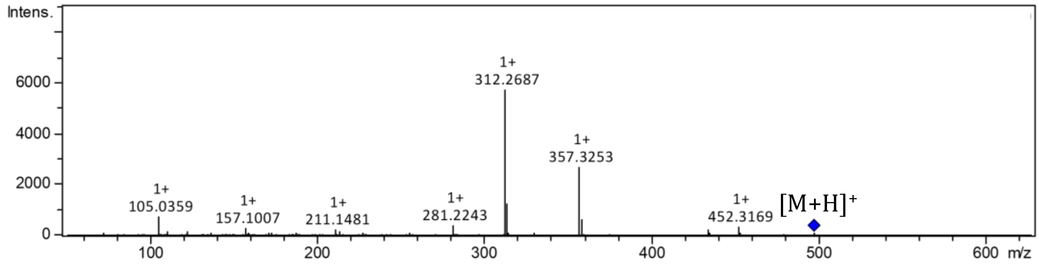

Figure S152. +ESI-QqTOF MS/MS spectrum of 5α-hydroxy-3α,4α-diapachysanaximine A (**17**).

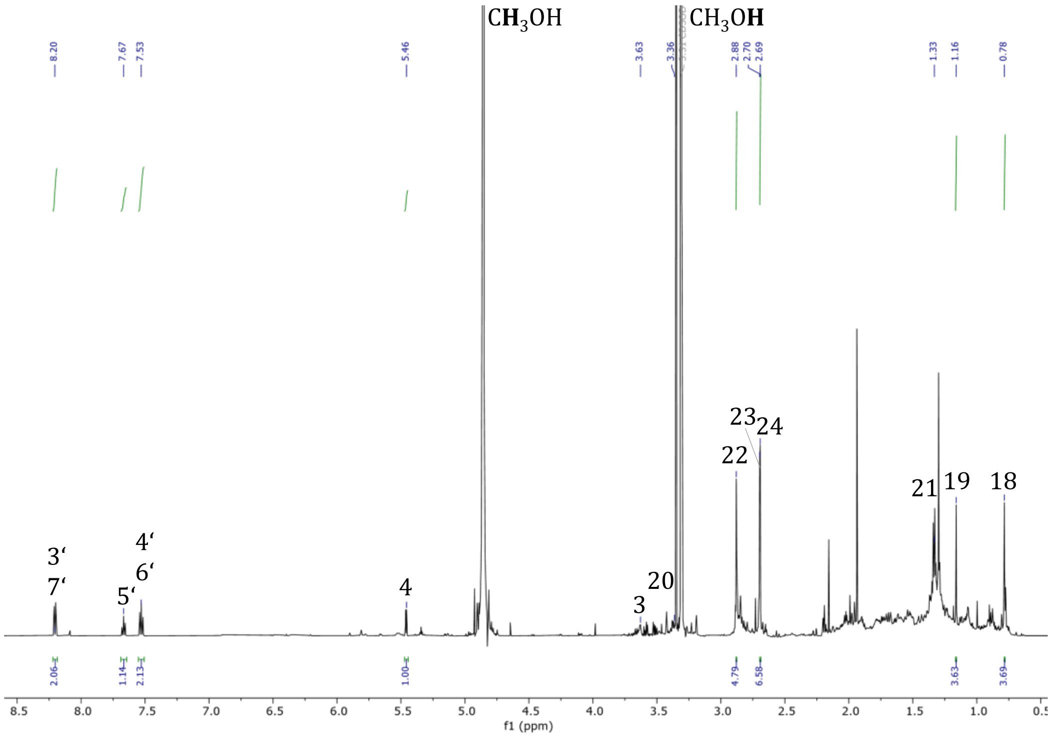

Figure S153. ^{1}H NMR spectrum of 5α-hydroxy-3α,4α-diapachysanaximine A (**17**) (CD_3OD, 600 MHz). The assignment of the signals between 3.15 and 0.55 ppm can be found in the enlarged Figure S154.

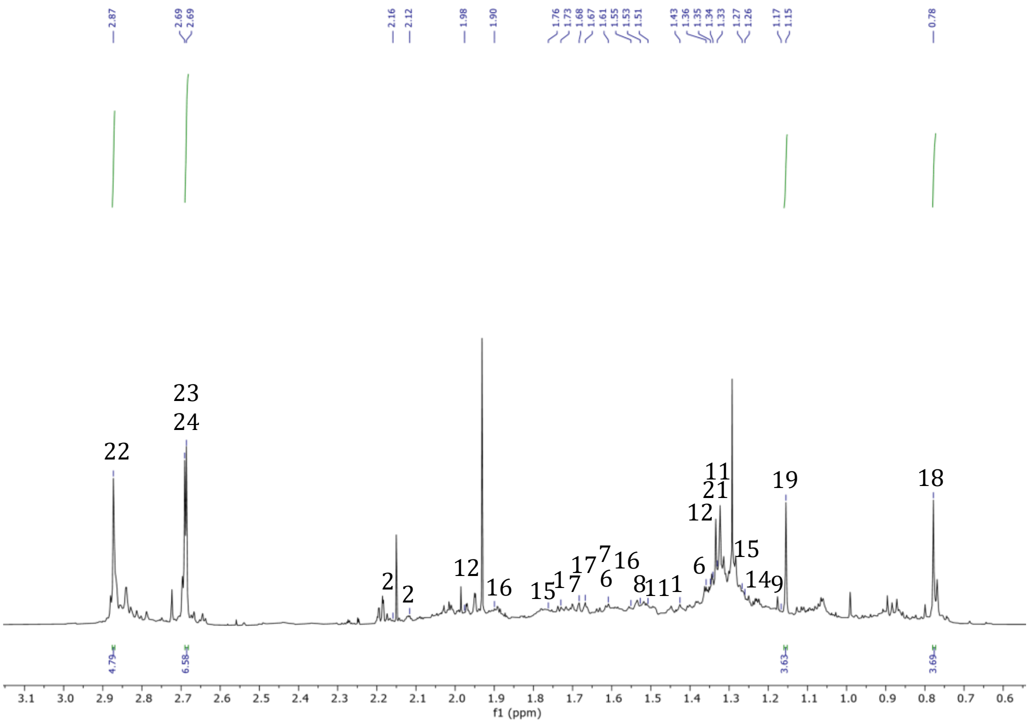

Figure S154. Detail of the ^{1}H NMR spectrum of 5α-hydroxy-3α,4α-diapachysanaximine A (**17**) (CD_3OD, 600 MHz).

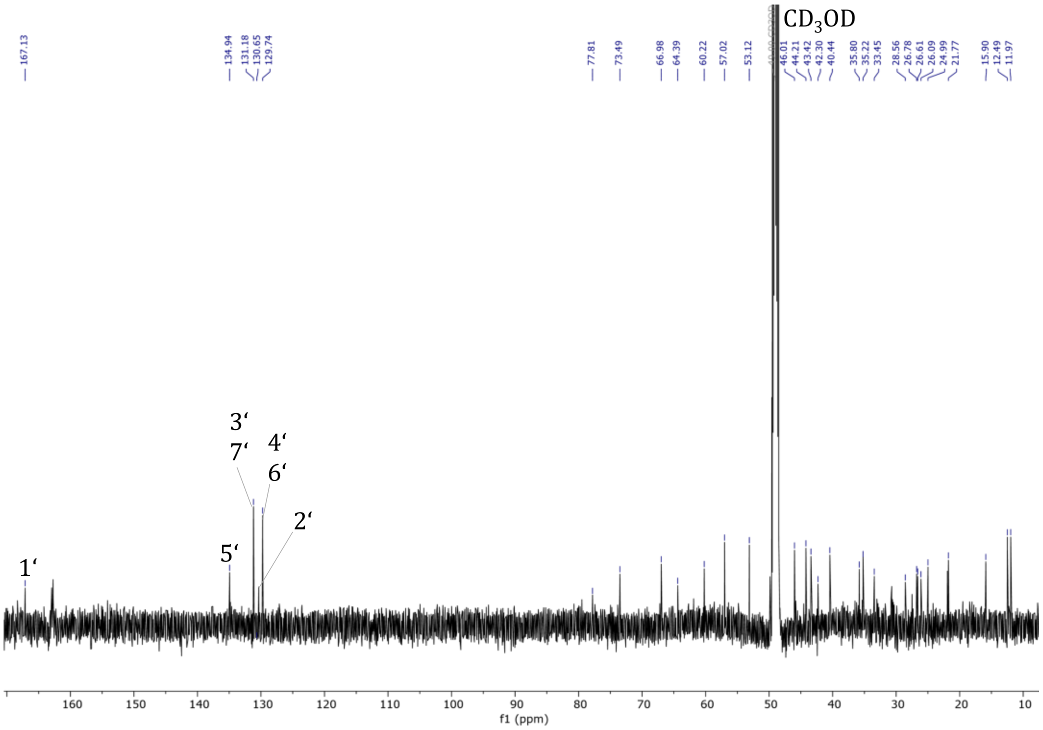

Figure S155. ^{13}C NMR spectrum of 5α-hydroxy-3α,4α-diapachysanaximine A (**17**) (CD_3OD, 151 MHz). The assignment of the signals between 80 and 10 ppm can be found in the enlarged Figure S156.

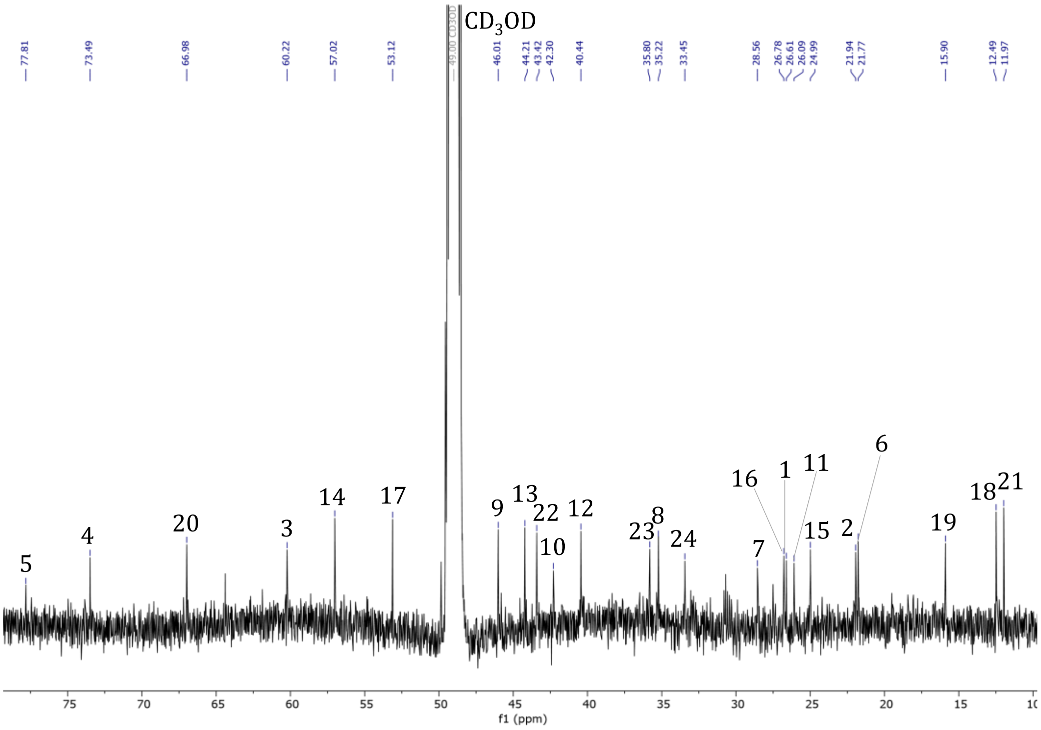

Figure S156. Detail of the ^{13}C NMR spectrum of 5α-hydroxy-3α,4α-diapachysanaximine A (**17**) (CD3OD, 151 MHz).

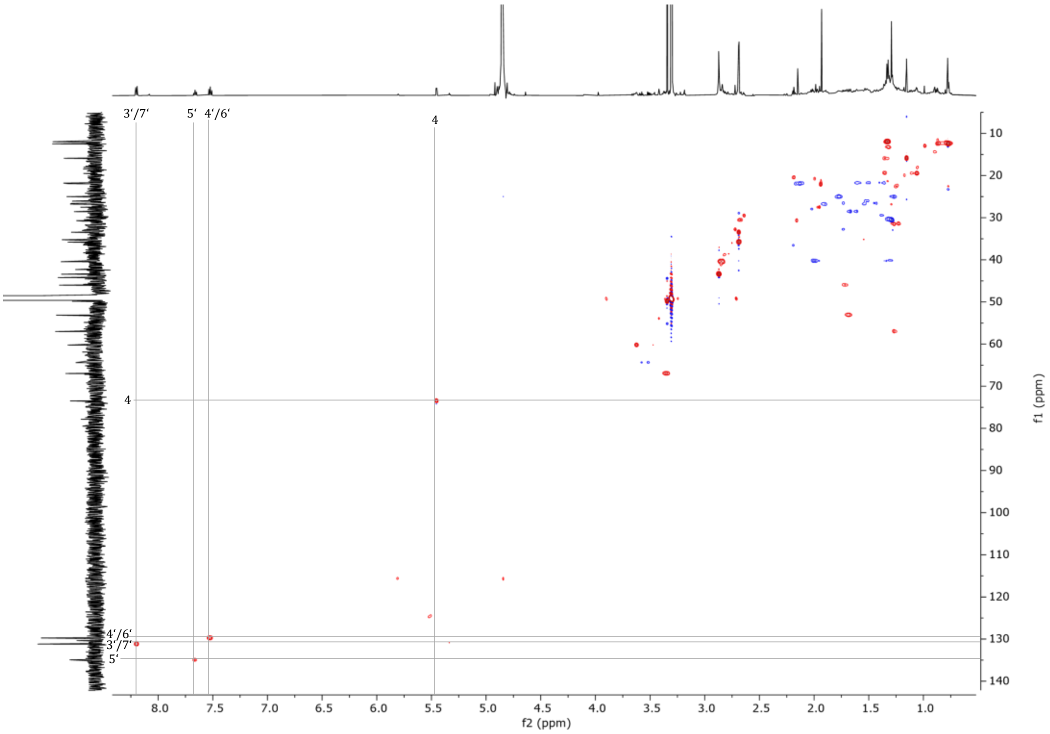

Figure S157. ^{1}H/^{13}C HSQC spectrum of 5α-hydroxy-3α,4α-diapachysanaximine A (**17**) (CD3OD, 600/151 MHz).

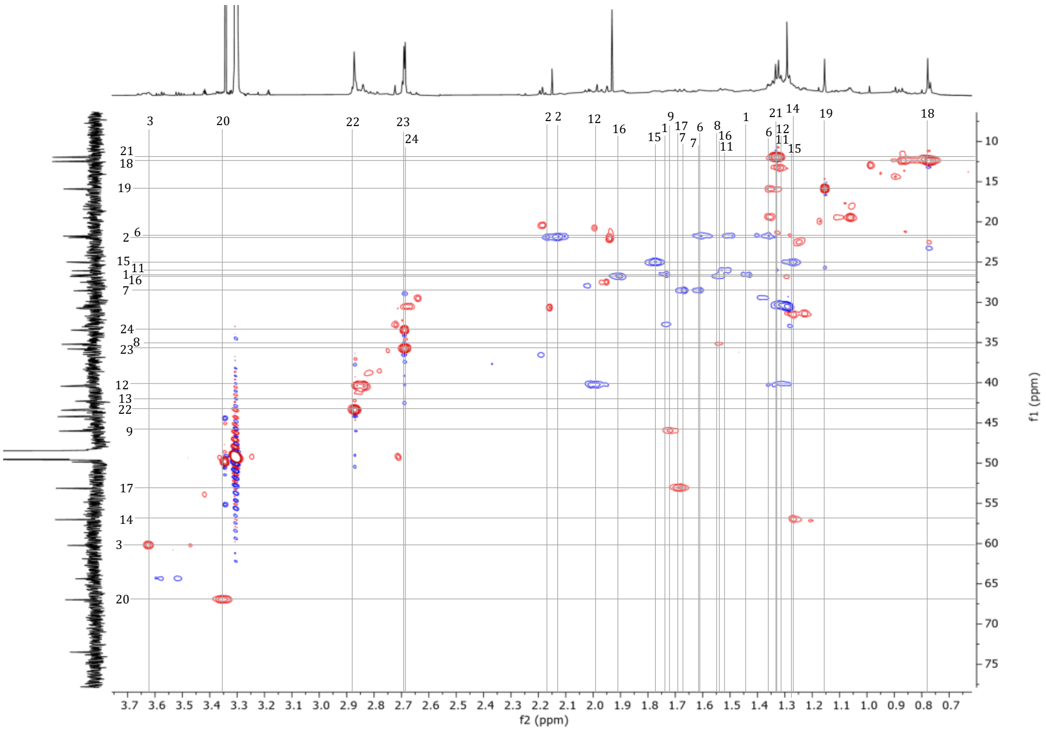

Figure S158. Detail of the $^{1}H/^{13}C$ HSQC spectrum of 5*α*-hydroxy-3*α*,4*α*-diapachysanaximine A (**17**) (CD_3OD, 600/151 MHz).

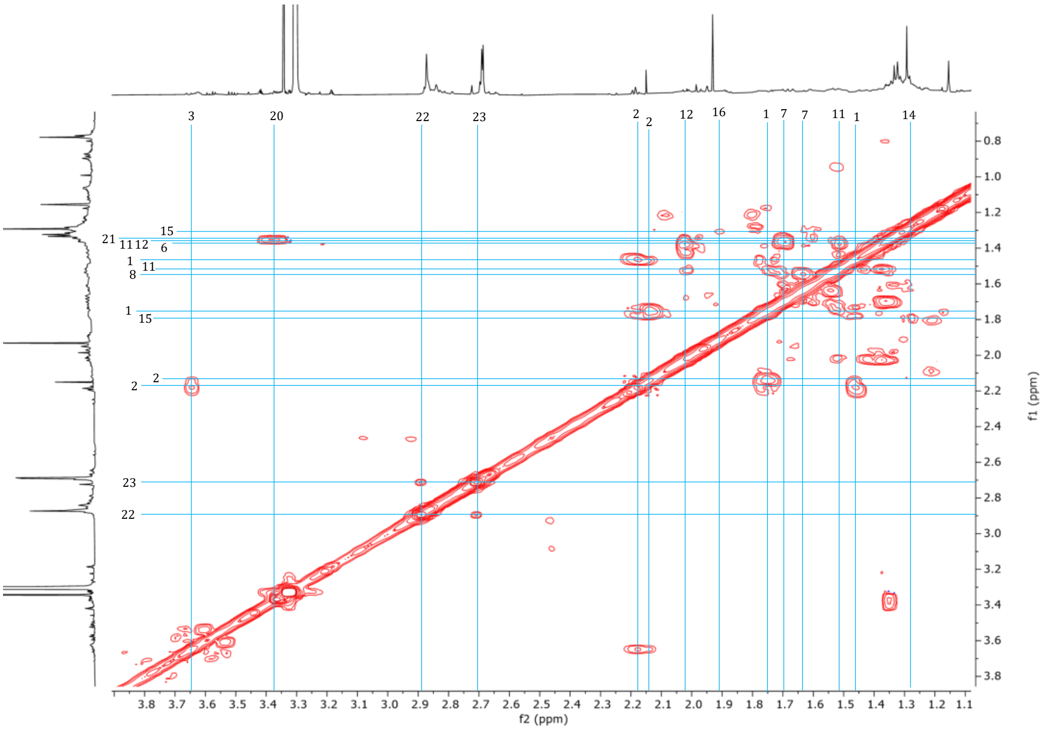

Figure S159. Detail of the $^{1}H/^{1}H$ COSY spectrum of 5*α*-hydroxy-3*α*,4*α*-diapachysanaximine A (**17**) (CD_3OD, 600 MHz).

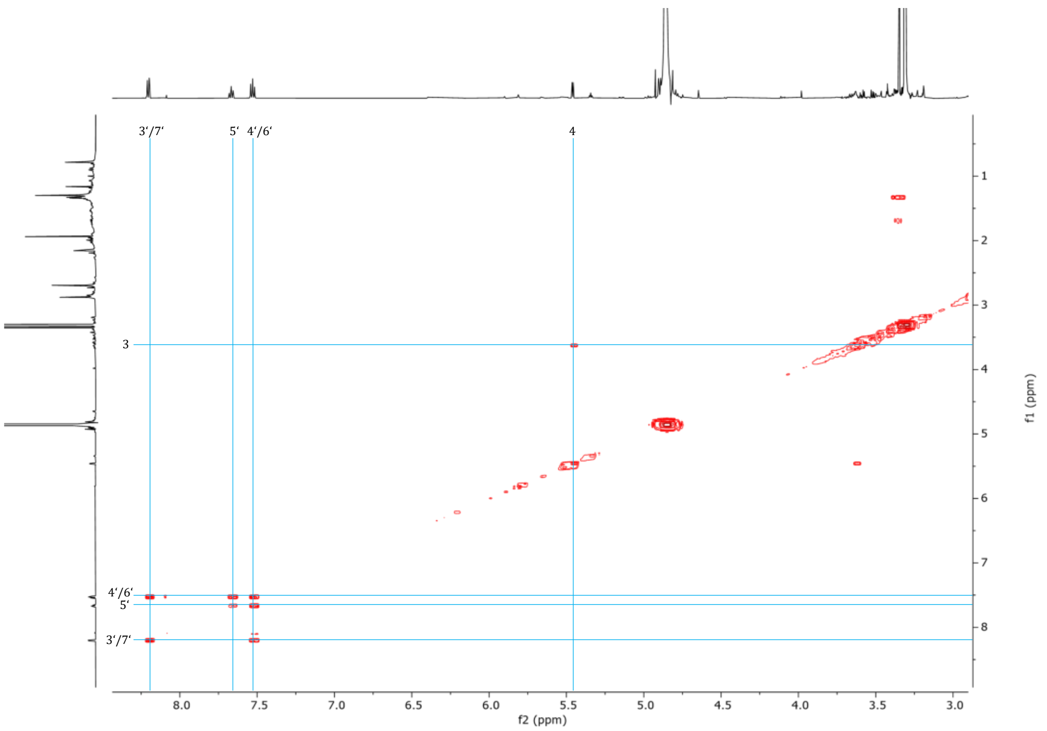

Figure S160. Detail of the $^{1}H/^{1}H$ COSY spectrum of 5α-hydroxy-3α,4α-diapachysanaximine A (**17**) (CD_3OD, 600 MHz).

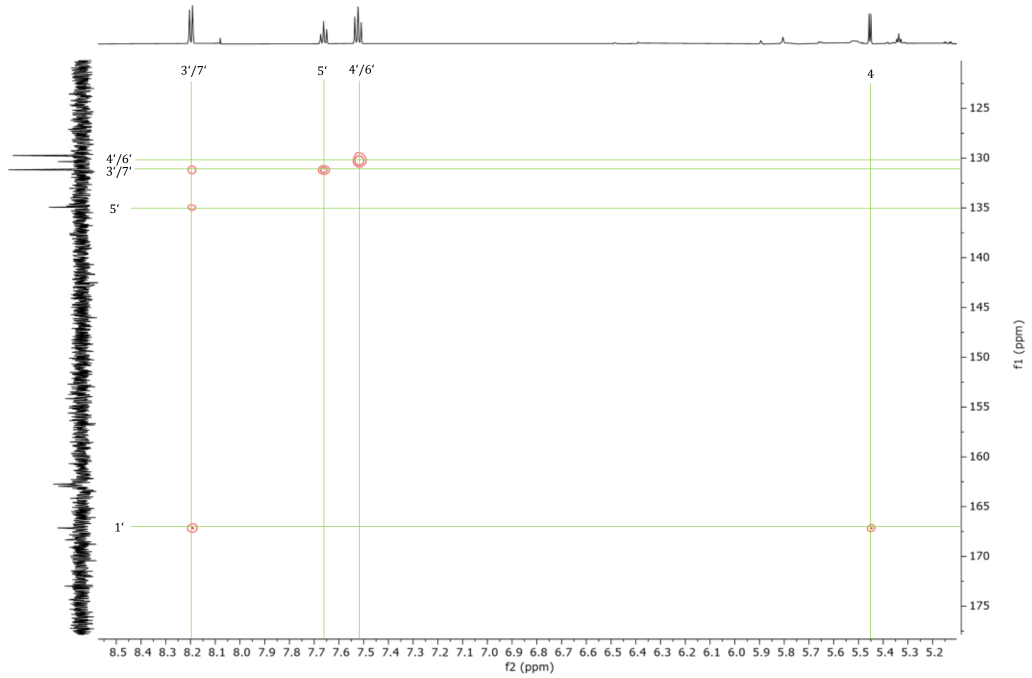

Figure S161. Detail of the $^{1}H/^{13}C$ HMBC spectrum of 5α-hydroxy-3α,4α-diapachysanaximine A (**17**) (CD_3OD, 600/151 MHz).

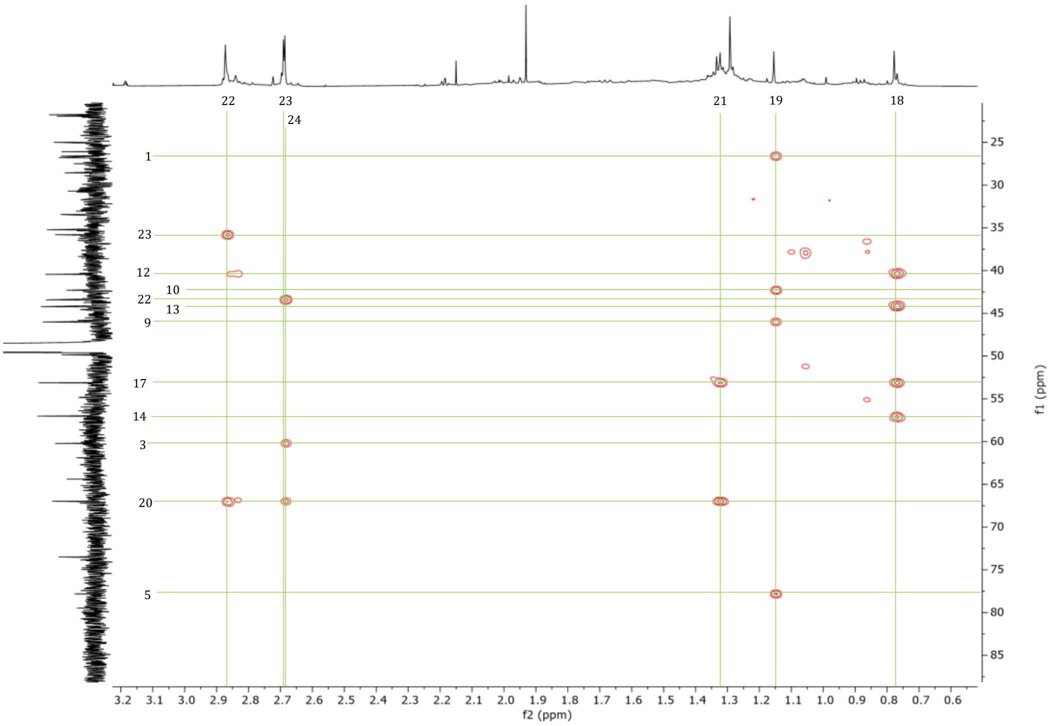

Figure S162. Detail of the ¹H/¹³C HMBC spectrum of 5α-hydroxy-3α,4α-diapachysanaximine A (**17**) (CD_3OD, 600/151 MHz).

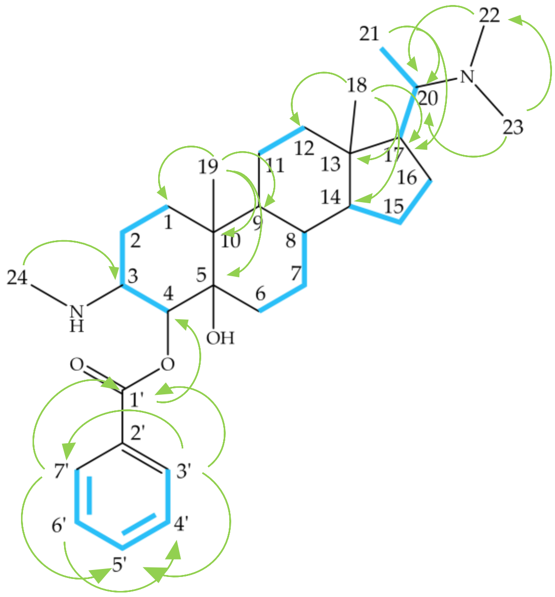

Figure S163. Key COSY (blue lines) and HMBC (green arrows) correlations of 5α-hydroxy-3α,4α-diapachysanaximine A (**17**).

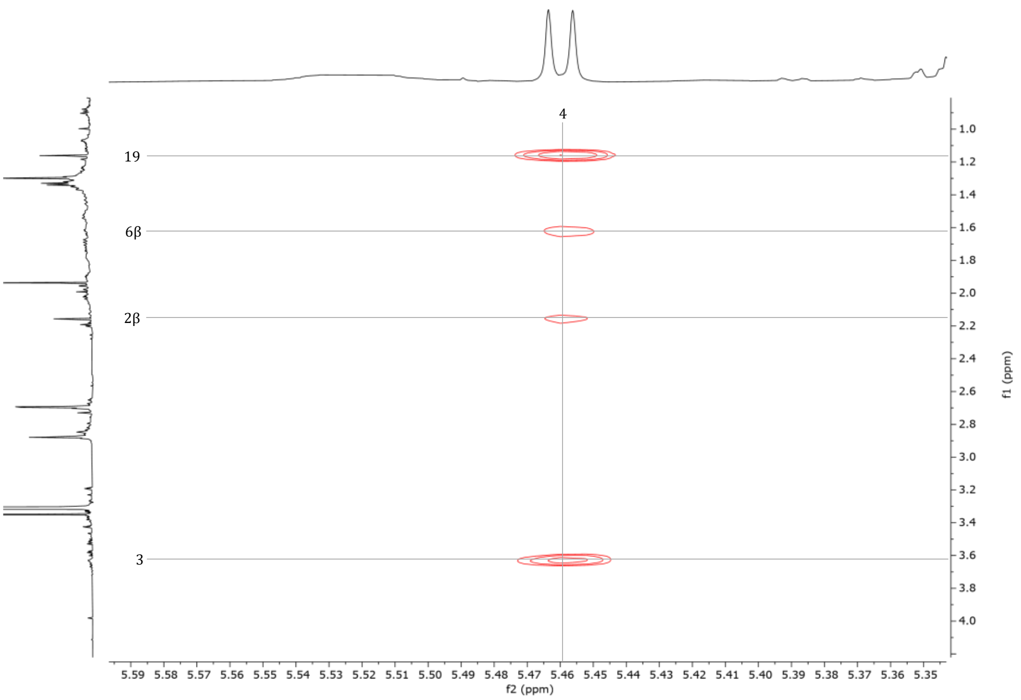

Figure S164. Detail of the $^{1}H/^{1}H$ NOESY spectrum of 5α-hydroxy-3α,4α-diapachysanaximine A (**17**) (CD_3OD, 600 MHz).

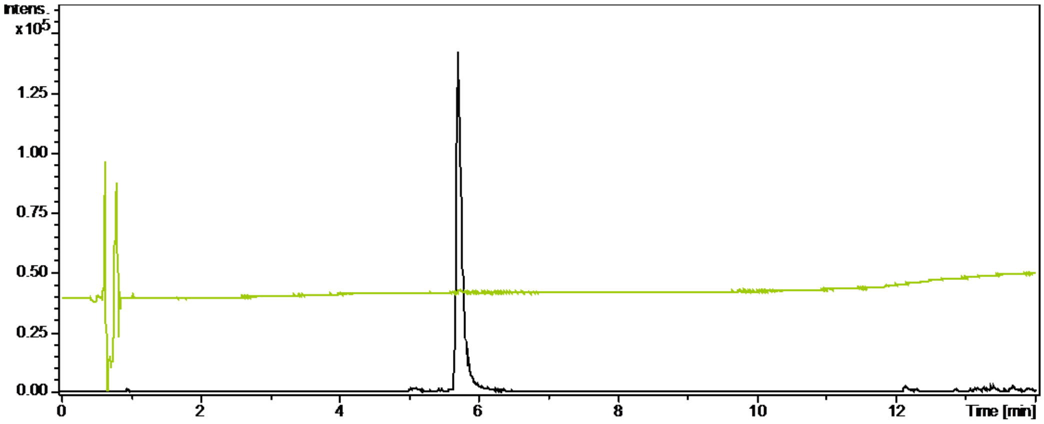

Figure S165. UHPLC/+ESI-QqTOF-MS/MS chromatogram of 2β,3β,4β-diapachysamine K (**18**). Base peak chromatogram 200.0000-1000.0000 +All MS (black); UV chromatogram 200-400 nm (green).

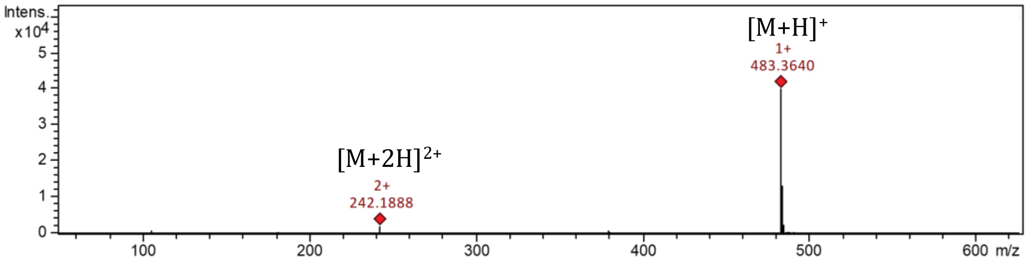

Figure S166. +ESI-QqTOF MS spectrum of 2β,3β,4β-diapachysamine K (**18**); *m/z* 483.3640 $[M+H]^{+}$, *m/z* 242.1888 $[M+2H]^{2+}$.

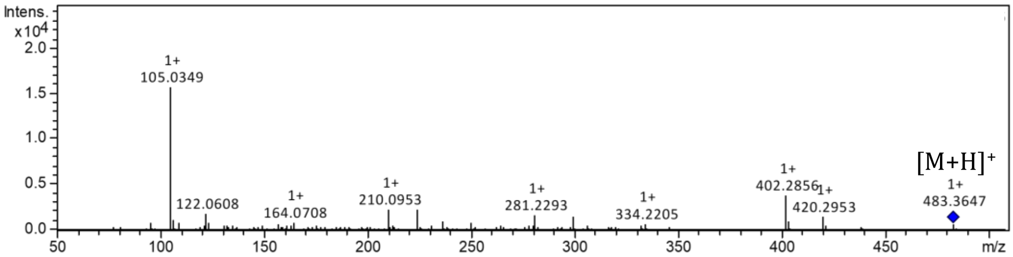

Figure S167. +ESI-QqTOF MS/MS spectrum of 2β,3β,4β-diapachysamine K (**18**).

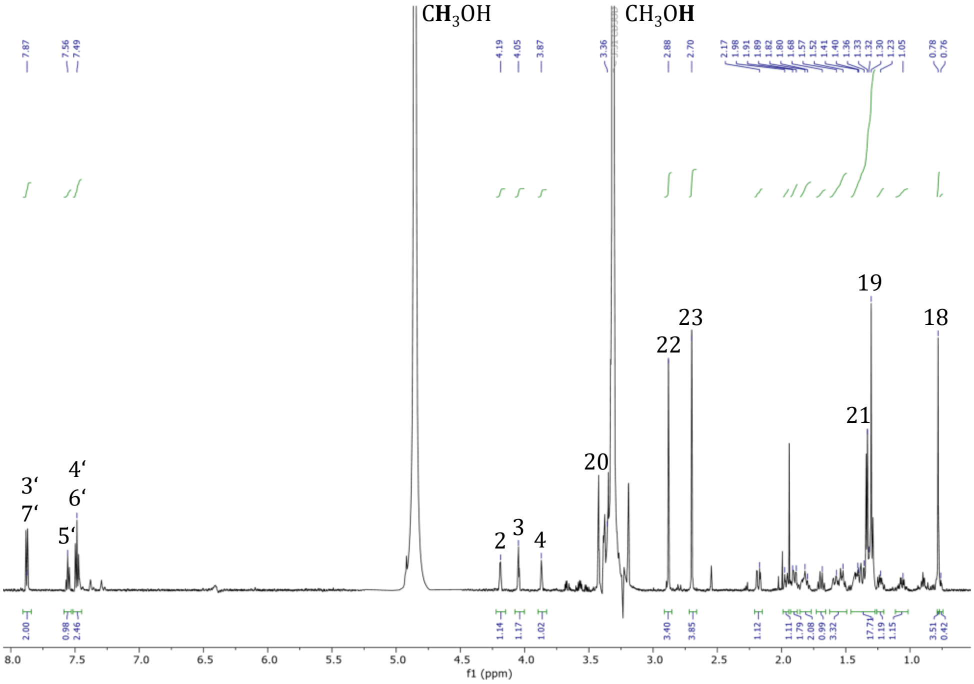

Figure S168. ^{1}H NMR spectrum of 2β,3β,4β-diapachysamine K (**18**) (CD_3OD, 600 MHz). The assignment of the signals between 2.95 and 0.65 ppm can be found in the enlarged Figure S169.

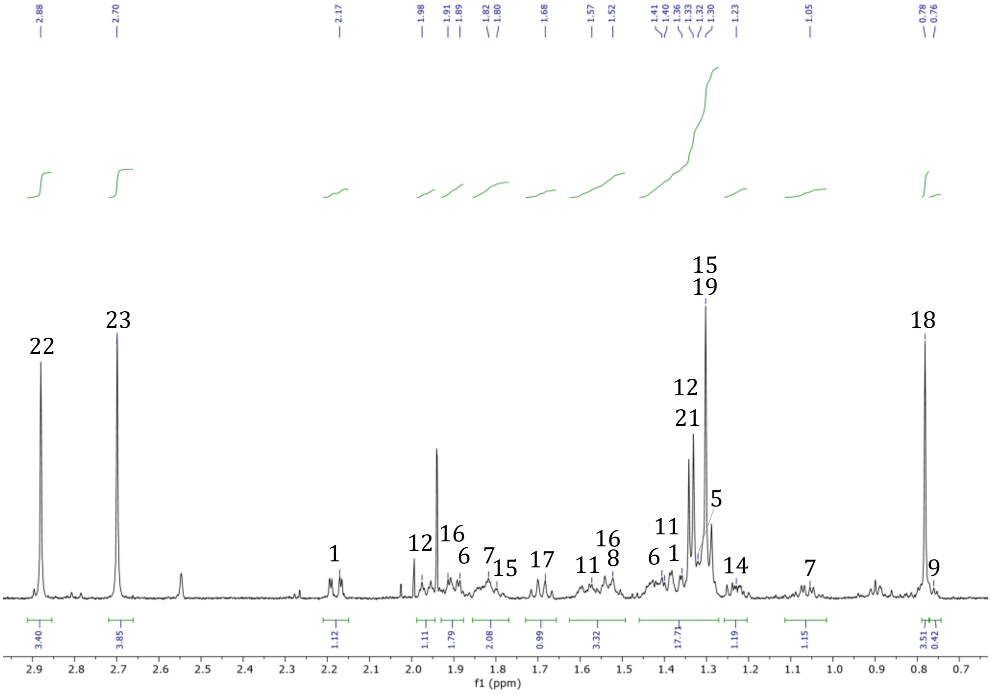

Figure S169. Detail of the ^{1}H NMR spectrum of 2β,3β,4β-diapachysamine K (**18**) (CD_3OD, 600 MHz).

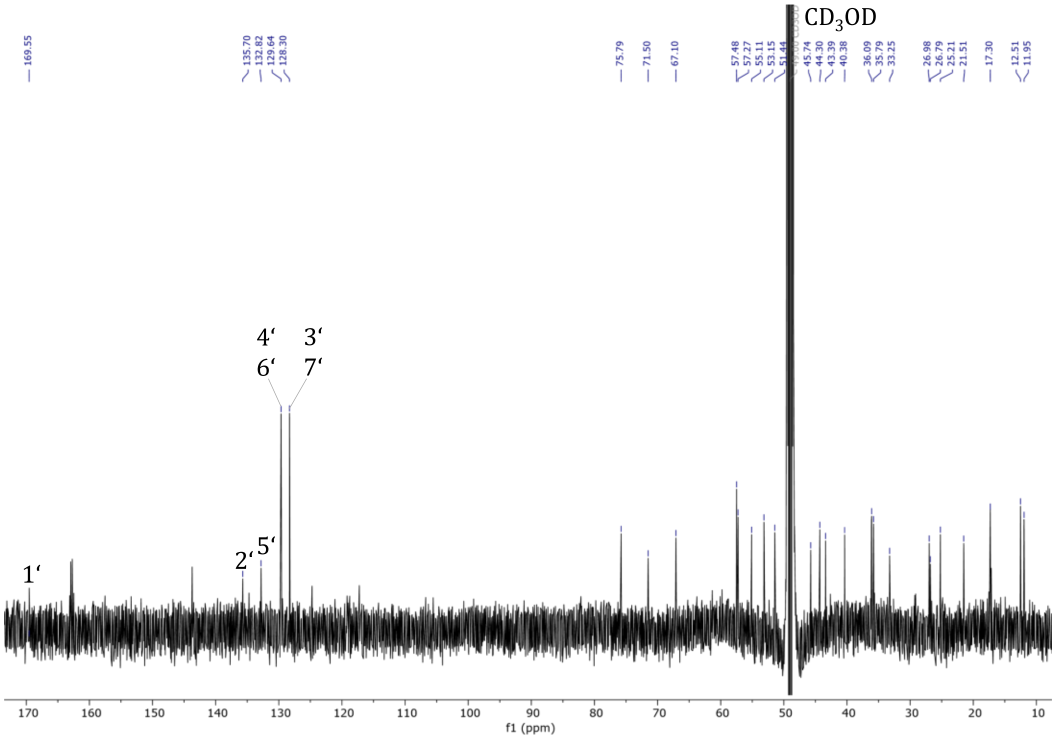

Figure S170. ^{13}C NMR spectrum of 2β,3β,4β-diapachysamine K (**18**) (CD_3OD, 151 MHz). The assignment of the signals between 80 and 10 ppm can be found in the enlarged Figure S171.

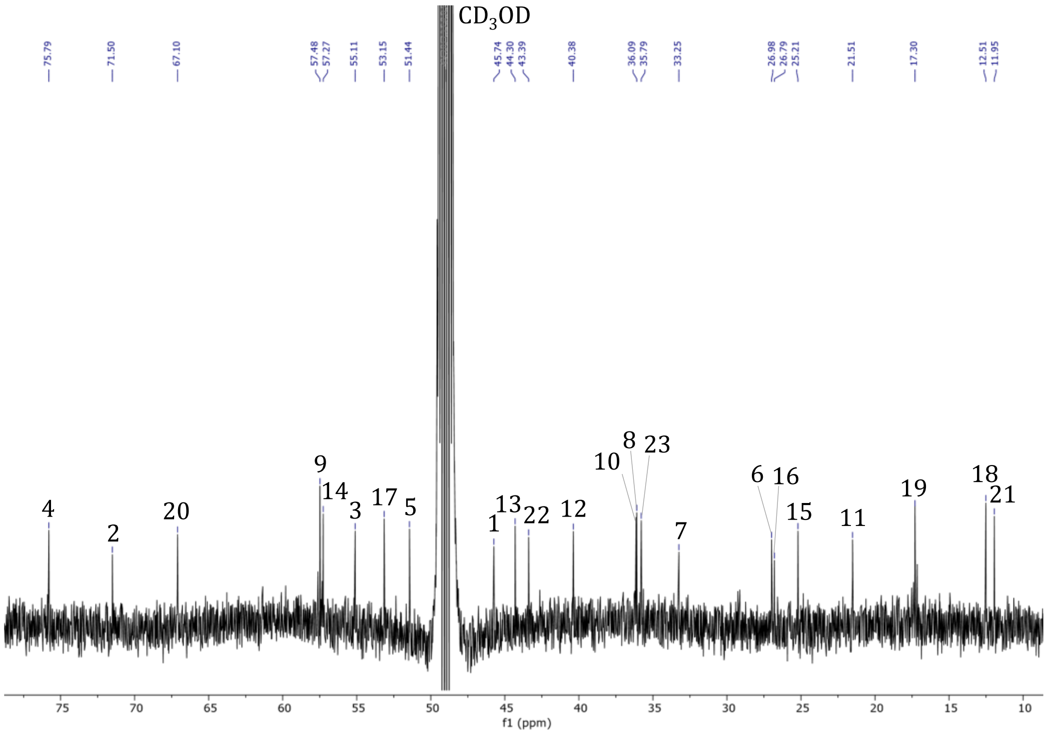

Figure S171. Detail of the ^{13}C NMR spectrum of 2β,3β,4β-diapachysamine K (**18**) (CD_3OD, 151 MHz).

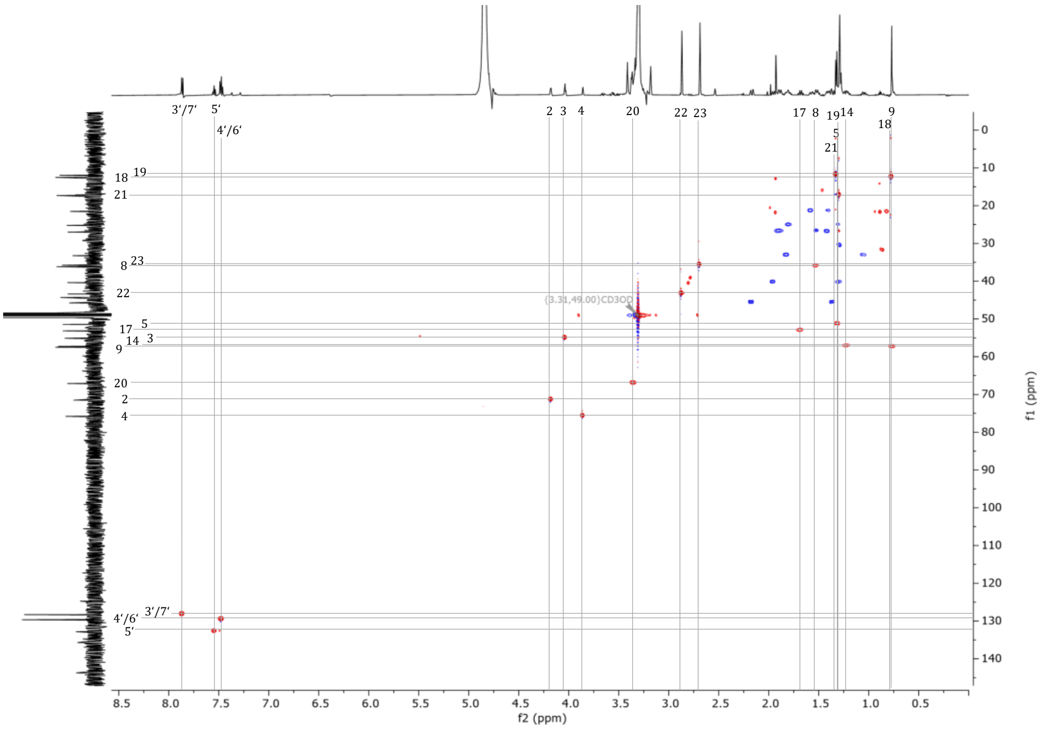

Figure S172. $^1H/^{13}C$ HSQC spectrum of 2β,3β,4β-diapachysamine K (**18**) (CD_3OD, 600/151 MHz).

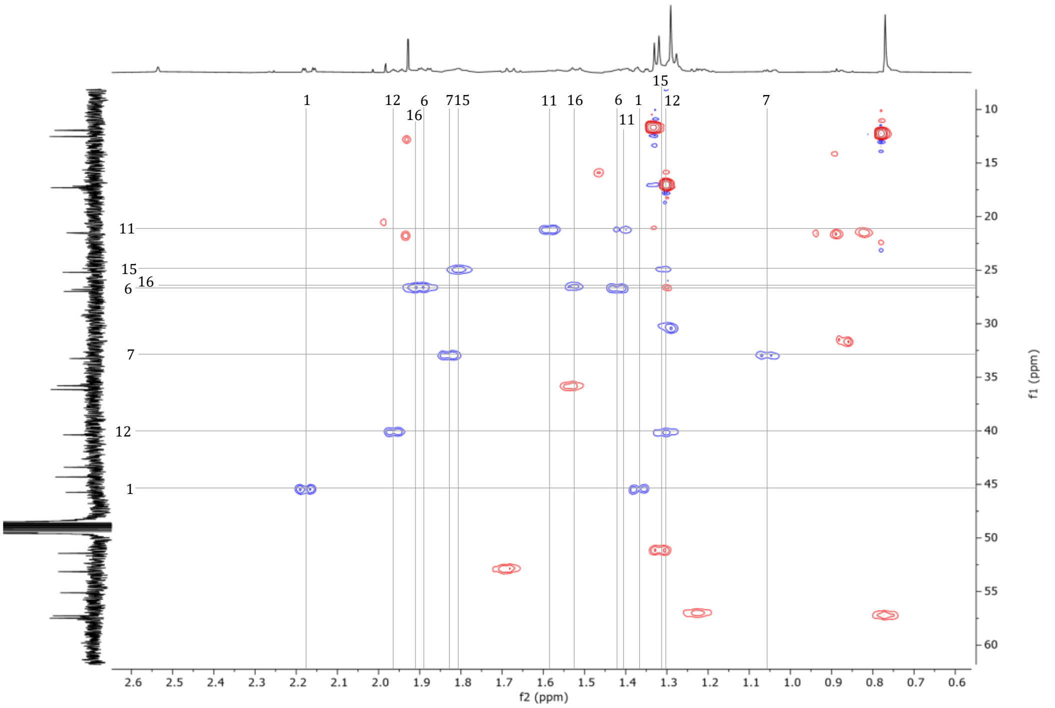

Figure S173. Detail of the $^1H/^{13}C$ HSQC spectrum of 2β,3β,4β-diapachysamine K (**18**) (CD_3OD, 600/151 MHz).

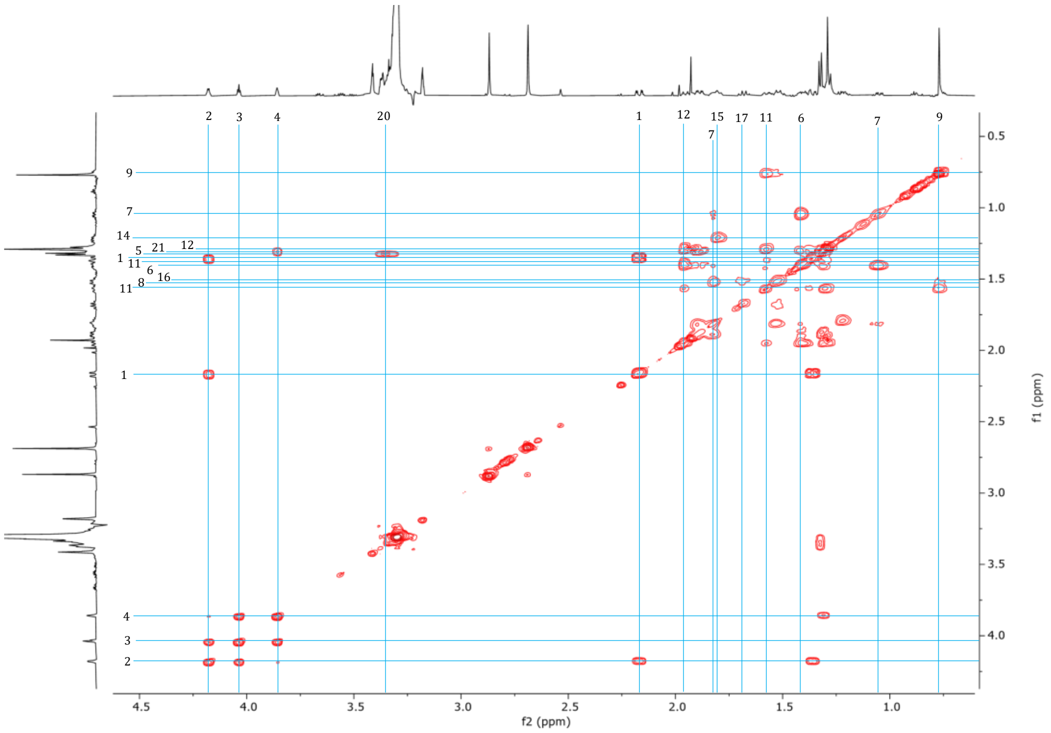

Figure S174. $^1H/^1H$ COSY spectrum of 2β,3β,4β-diapachysamine K (**18**) (CD_3OD, 600 MHz).

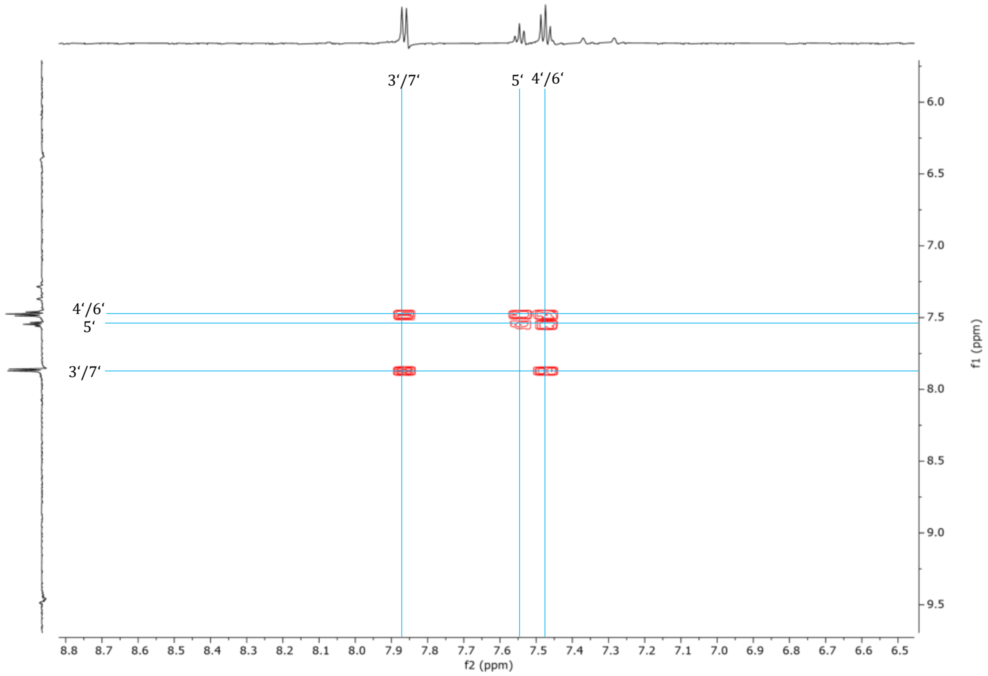

Figure S175. $^1H/^{13}C$ HMBC spectrum of 2β,3β,4β-diapachysamine K (**18**) (CD_3OD, 600/151 MHz).

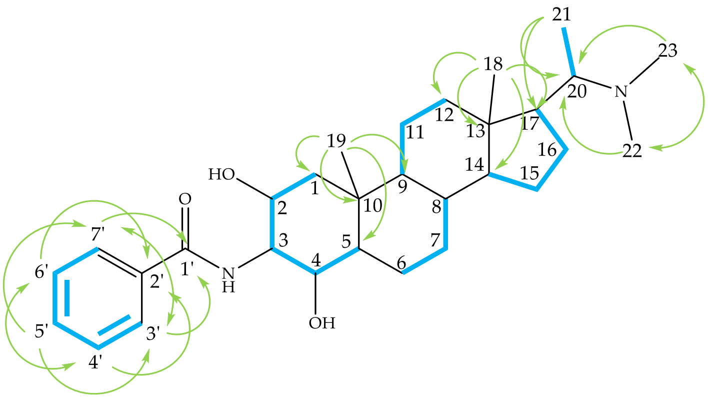

Figure S176. Key COSY (blue lines) and HMBC (green arrows) correlations of 2β,3β,4β-diapachysamine K (**18**).

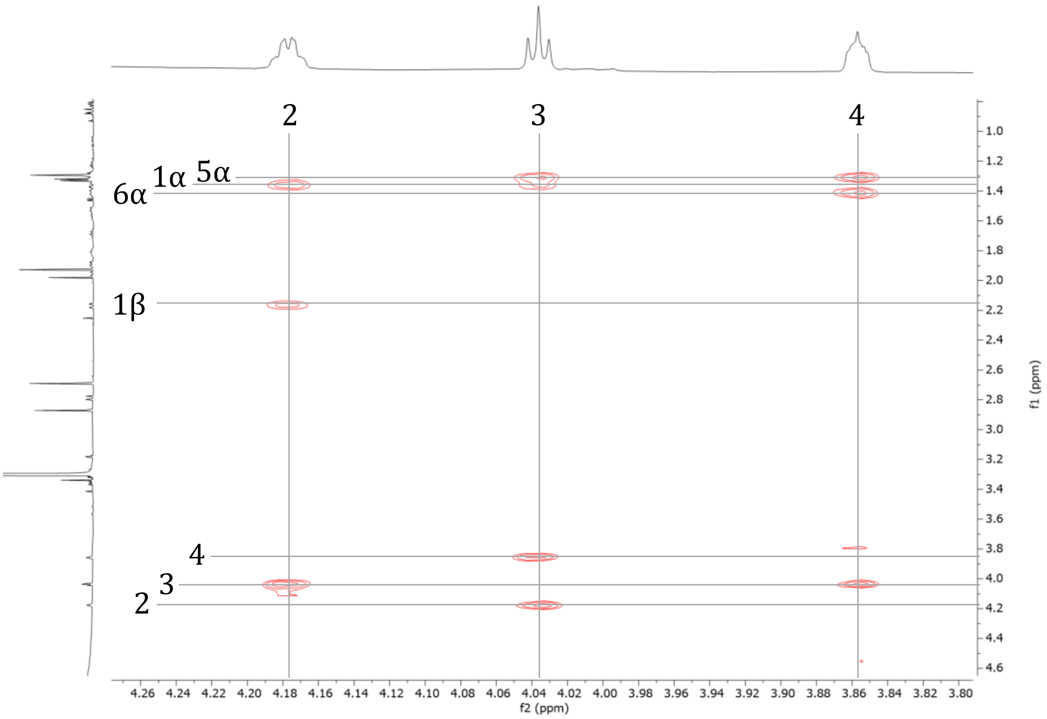

Figure S177. Detail of the $^{1}H/^{1}H$ NOESY spectrum of 2β,3β,4β-diapachysamine K (**18**) (CD_3OD, 600 MHz).

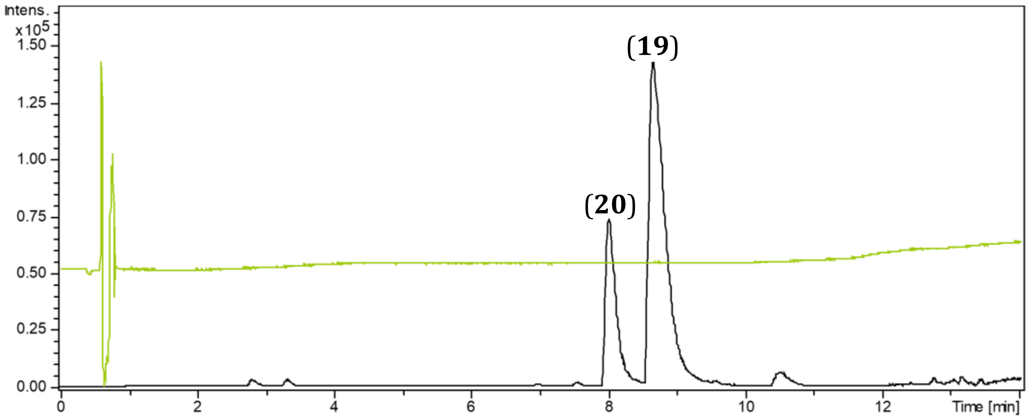

Figure S178. UHPLC/+ESI-QqTOF-MS/MS chromatogram of 3β-dimethylamino-pregnane-20-oxime (**19**) and 3β-dimethylamino-pregn-5,6-ene-20-oxime (**20**). Base peak chromatogram 200.0000-1000.0000 +All MS (black); UV chromatogram 200-400 nm (green).

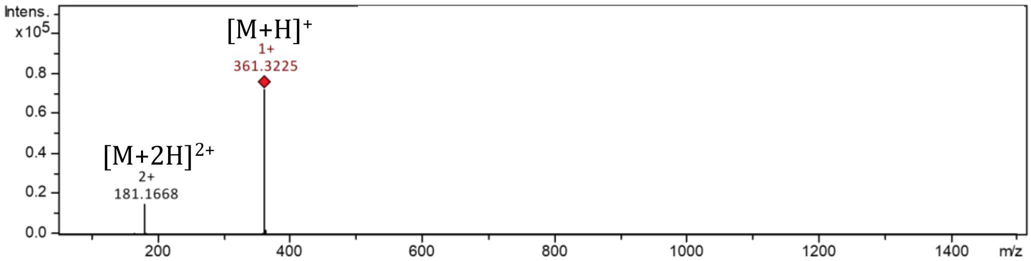

Figure S179. +ESI-QqTOF MS spectrum of 3β-dimethylamino-pregnane-20-oxime (**19**); *m/z* 361.3225 $[M+H]^+$, *m/z* 181.1668 $[M+2H]^{2+}$.

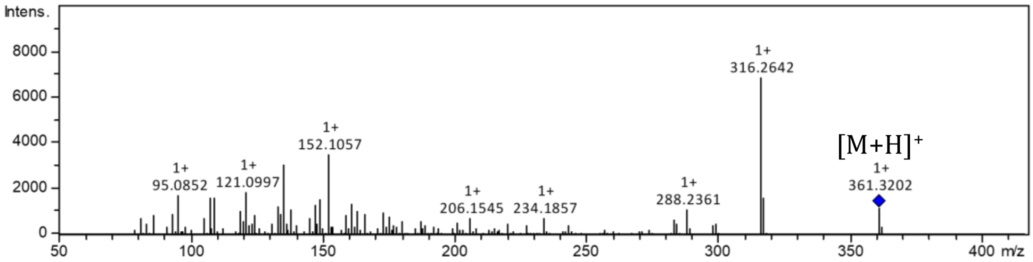

Figure S180. +ESI-QqTOF MS/MS spectrum of 3β-dimethylamino-pregnane-20-oxime (**19**).

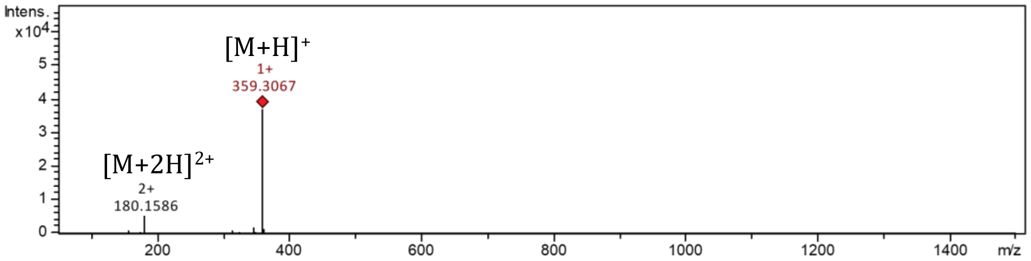

Figure S181. +ESI-QqTOF MS spectrum of 3β-dimethylamino-pregn-5,6-ene-20-oxime (**20**); *m/z* 359.3067 $[M+H]^+$, *m/z* 180.1586 $[M+2H]^{2+}$.

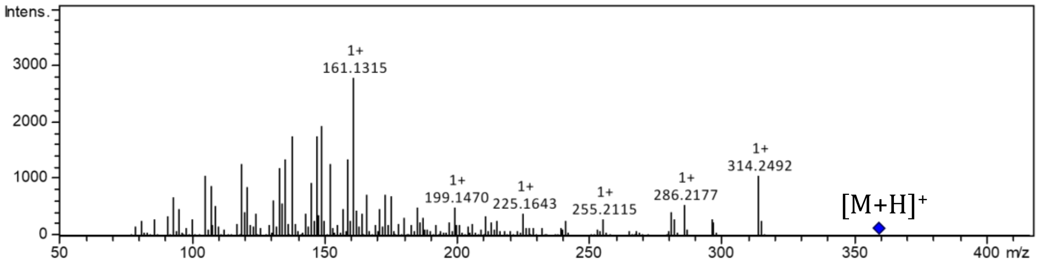

Figure S182. +ESI-QqTOF MS/MS spectrum of 3β-dimethylamino-pregn-5,6-ene-20-oxime (**20**).

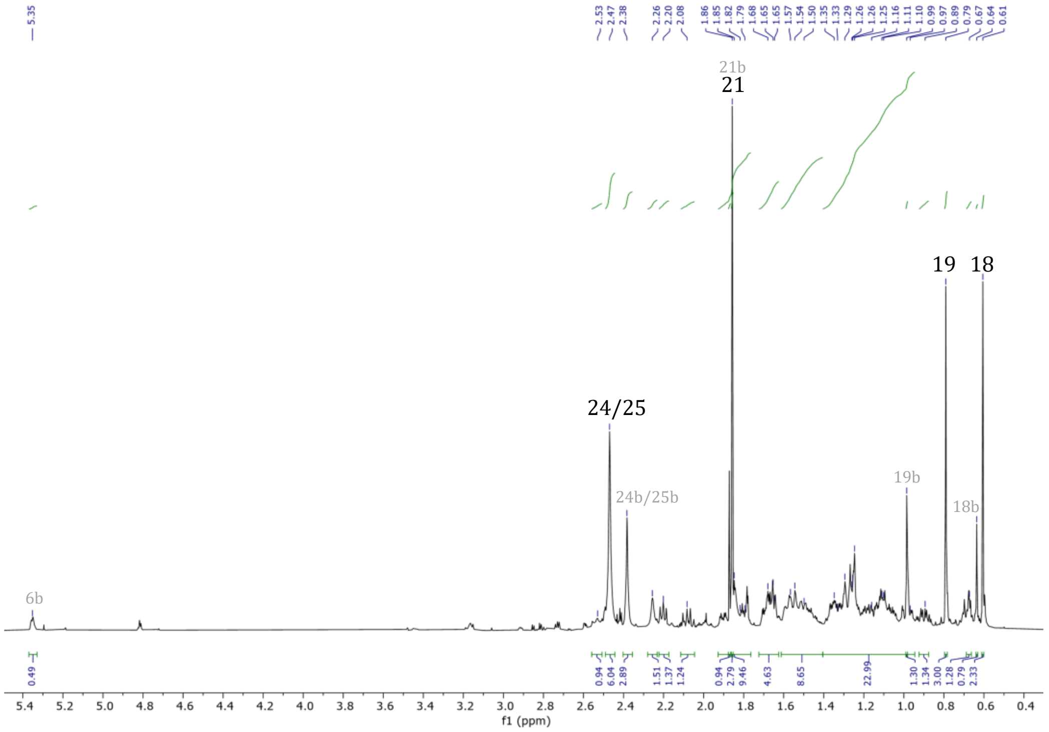

Figure S183. ^{1}H NMR spectrum of 3β-dimethylamino-pregnane-20-oxime (**19**) and 3β-dimethylamino-pregn-5,6-ene-20-oxime (**20** (b)) ($CDCl_3$, 600 MHz). The assignment of the signals between 2.60 and 0.55 ppm can be found in the enlarged Figure S184.

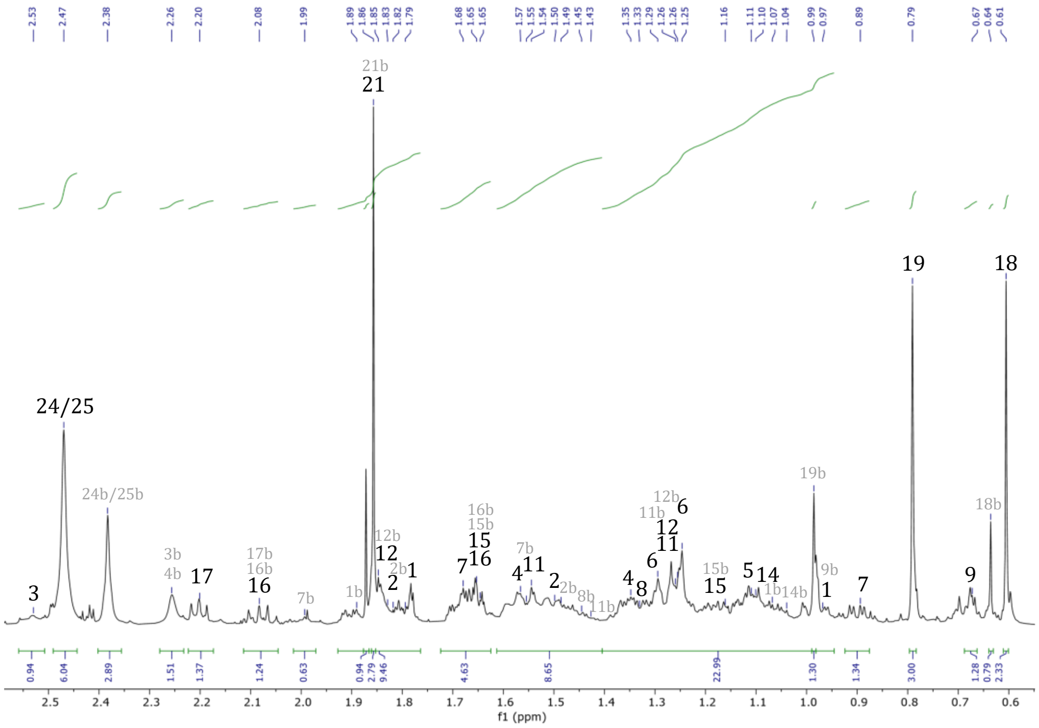

Figure S184. Detail of the ^{1}H NMR spectrum of 3β-dimethylamino-pregnane-20-oxime (**19**) and 3β-dimethylamino-pregn-5,6-ene-20-oxime (**20** (b)) ($CDCl_3$, 600 MHz).

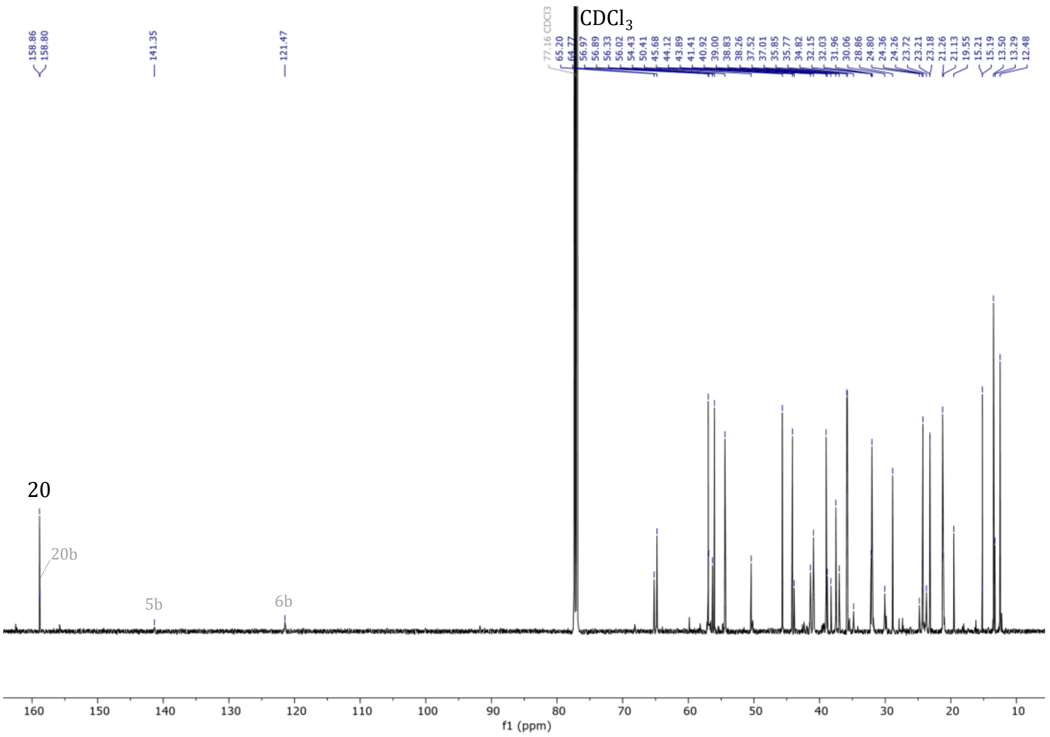

Figure S185. ^{13}C NMR spectrum of 3β-dimethylamino-pregnane-20-oxime (**19**) and 3β-dimethylamino-pregn-5,6-ene-20-oxime (**20** (b)) ($CDCl_3$, 151 MHz). The assignment of the signals between 65 and 10 ppm can be found in the enlarged Figure S186.

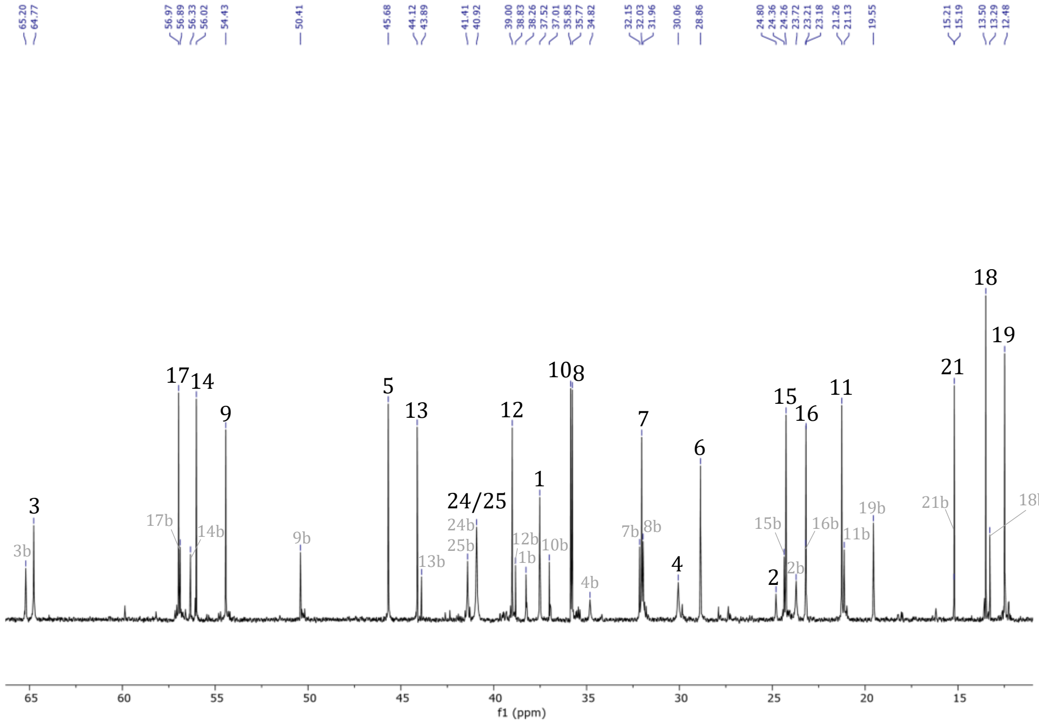

Figure S186. Detail of the ^{13}C NMR spectrum of 3β-dimethylamino-pregnane-20-oxime (**19**) and 3β-dimethylamino-pregn-5,6-ene-20-oxime (**20** (b)) ($CDCl_3$, 151 MHz).

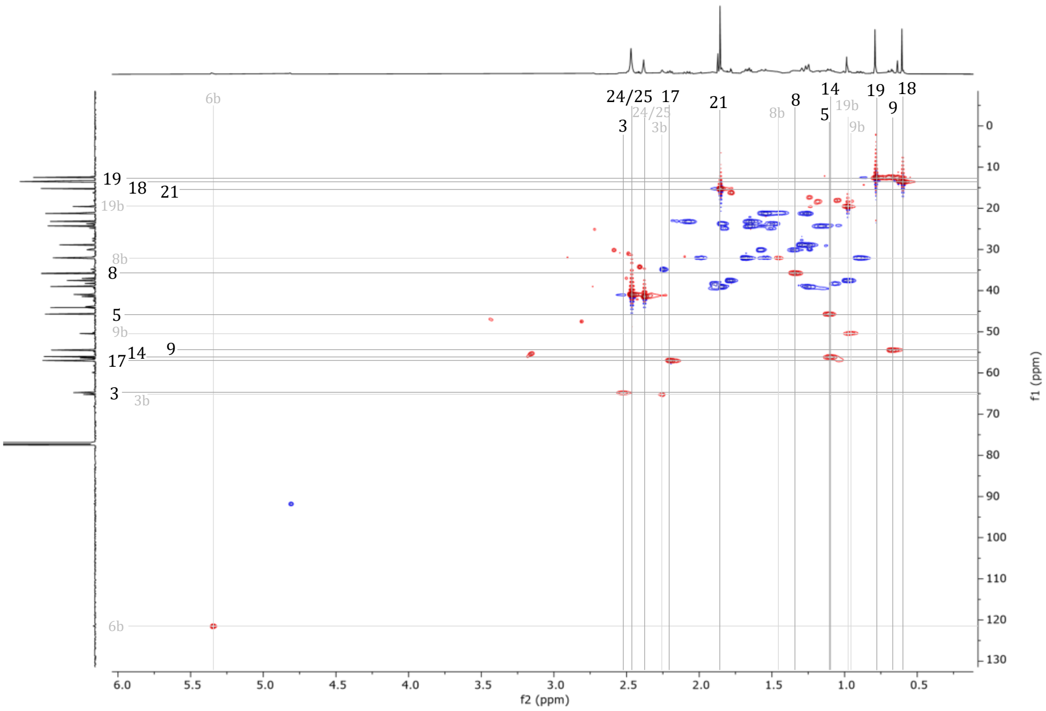

Figure S187. $^{1}H/^{13}C$ HSQC spectrum of 3β-dimethylamino-pregnane-20-oxime (**19**) and 3β-dimethylamino-pregn-5,6-ene-20-oxime (**20** (b)) ($CDCl_3$, 600/151 MHz).

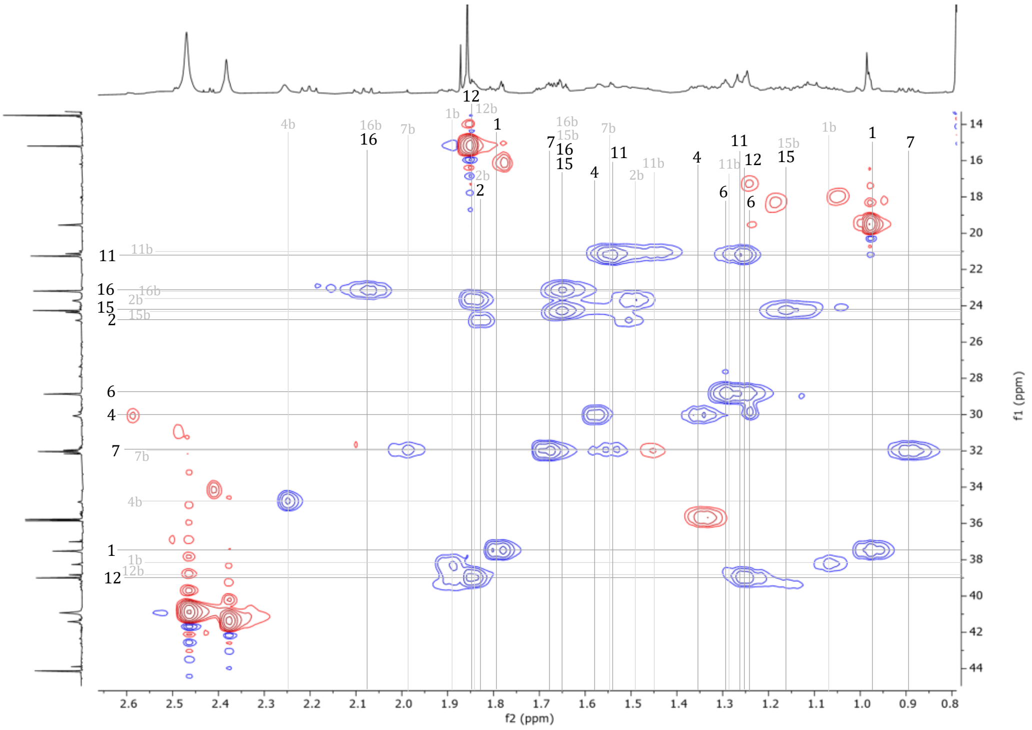

Figure S188. Detail of the $^{1}H/^{13}C$ HSQC spectrum of 3β-dimethylamino-pregnane-20-oxime (**19**) and 3β-dimethylamino-pregn-5,6-ene-20-oxime (**20** (b)) ($CDCl_3$, 600/151 MHz).

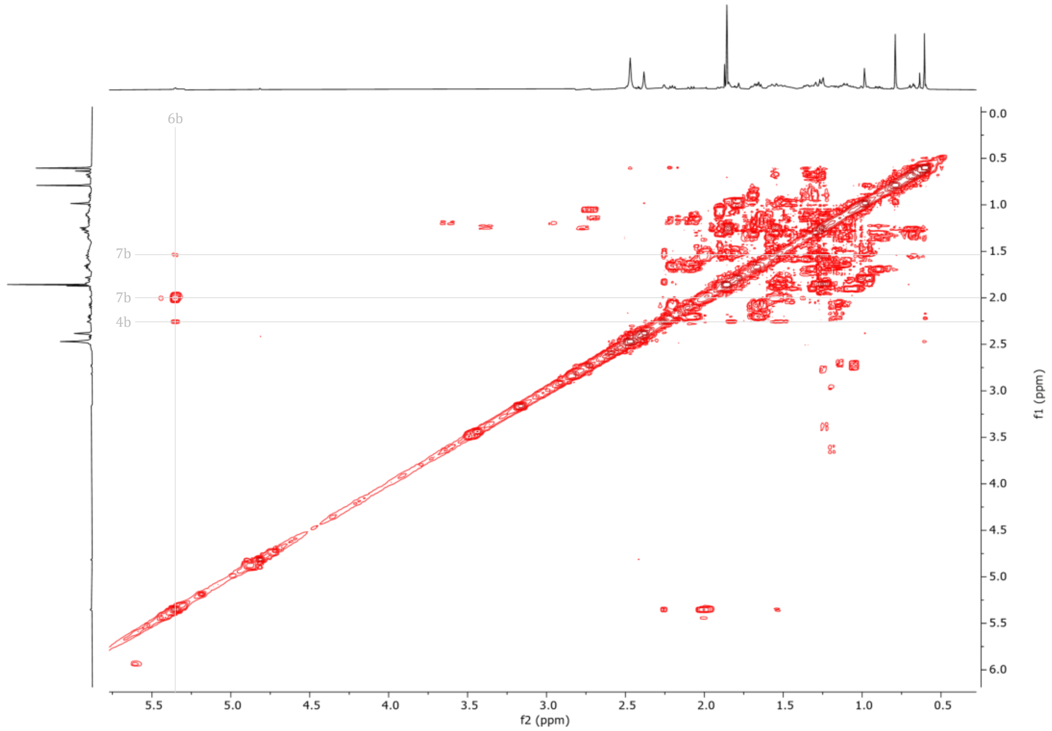

Figure S189. $^1H/^1H$ COSY spectrum of 3β-dimethylamino-pregnane-20-oxime (**19**) and 3β-dimethylamino-pregn-5,6-ene-20-oxime (**20** (b)) ($CDCl_3$, 600 MHz).

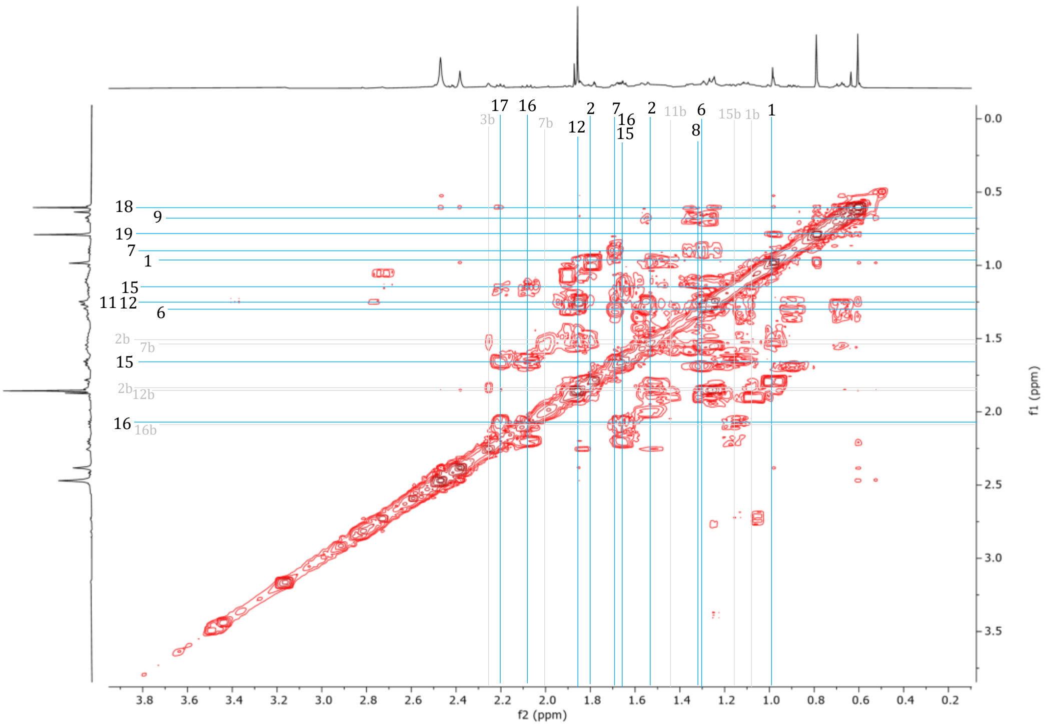

Figure S190. Detail of the $^1H/^1H$ COSY spectrum of 3β-dimethylamino-pregnane-20-oxime (**19**) and 3β-dimethylamino-pregn-5,6-ene-20-oxime (**20** (b)) ($CDCl_3$, 600 MHz).

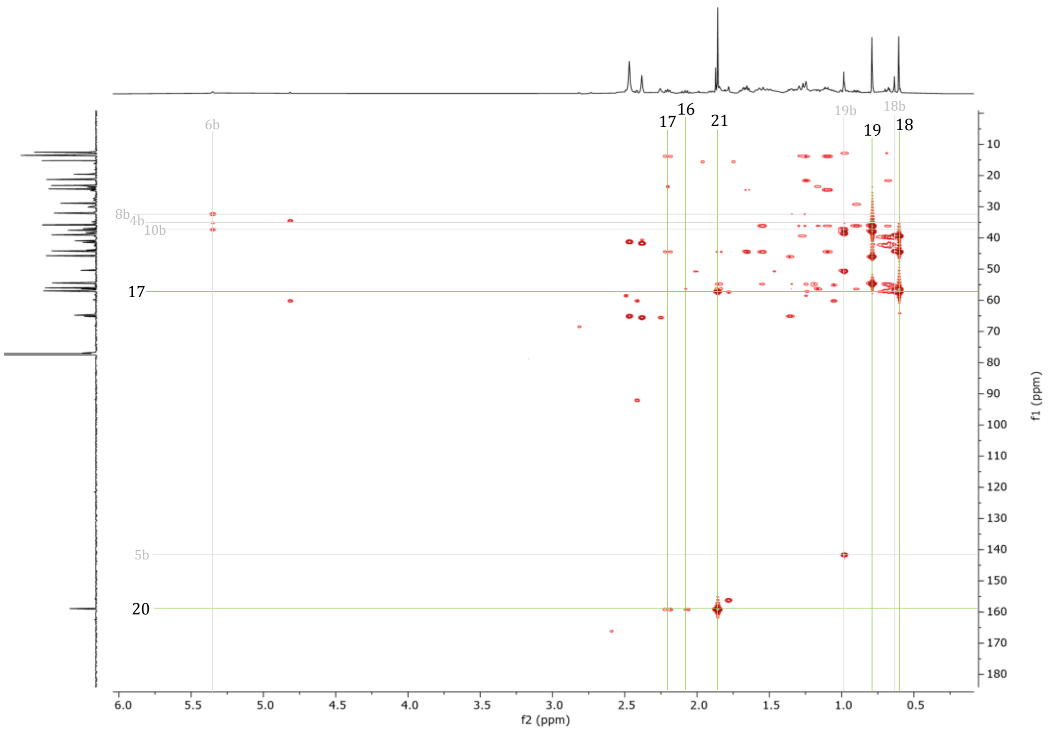

Figure S191. $^1H/^{13}C$ HMBC spectrum of 3β-dimethylamino-pregnane-20-oxime (**19**) and 3β-dimethylamino-pregn-5,6-ene-20-oxime (**20** (b)) ($CDCl_3$, 600/151 MHz).

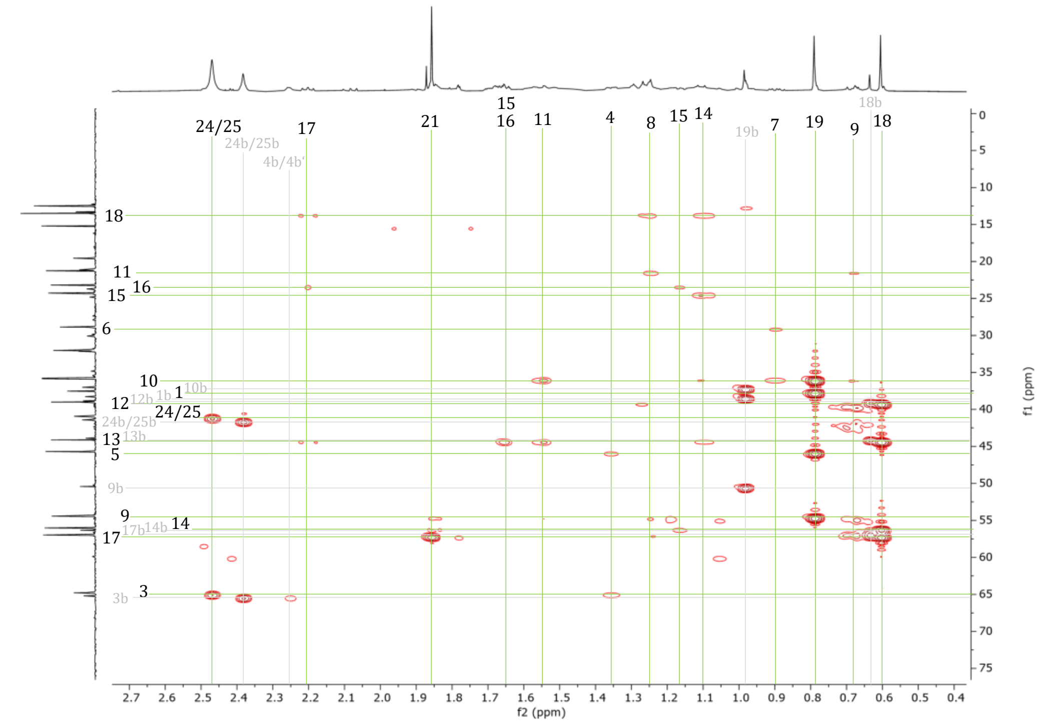

Figure S192. Detail of the $^1H/^{13}C$ HMBC spectrum of 3β-dimethylamino-pregnane-20-oxime (**19**) and 3β-dimethylamino-pregn-5,6-ene-20-oxime (**20** (b)) ($CDCl_3$, 600/151 MHz).

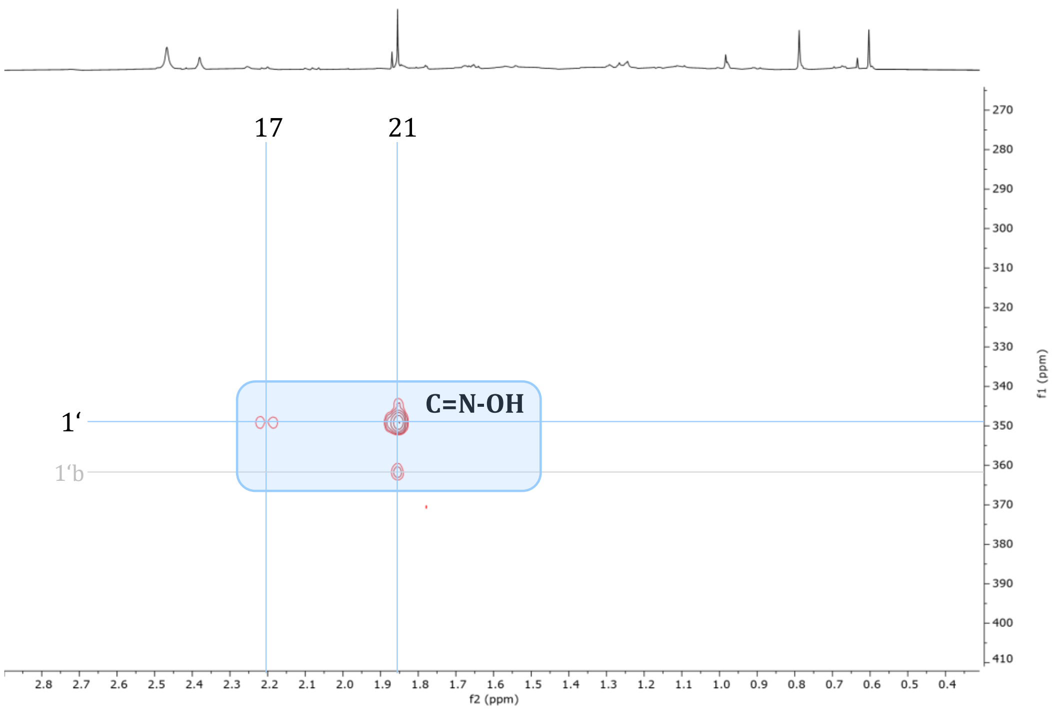

Figure S193. Detail of the $^{1}H/^{15}N$ HMBC spectrum of 3β-dimethylamino-pregnane-20-oxime (**19**) and 3β-dimethylamino-pregn-5,6-ene-20-oxime (**20** (b)) ($CDCl_3$, 600/151 MHz).

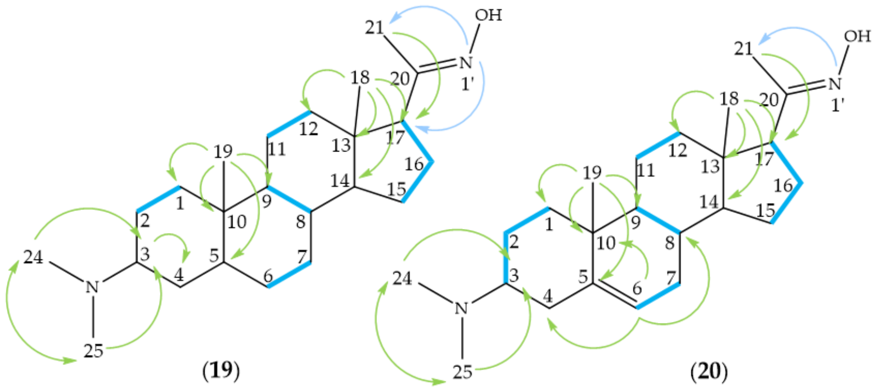

Figure S194. Key COSY (blue lines) and HMBC ($^{1}H/^{13}C$: green arrows, $^{1}H/^{15}N$ blue arrows) correlations of 3β-dimethylamino-pregnane-20-oxime (**19**) and 3β-dimethylamino-pregn-5,6-ene-20-oxime (**20**).

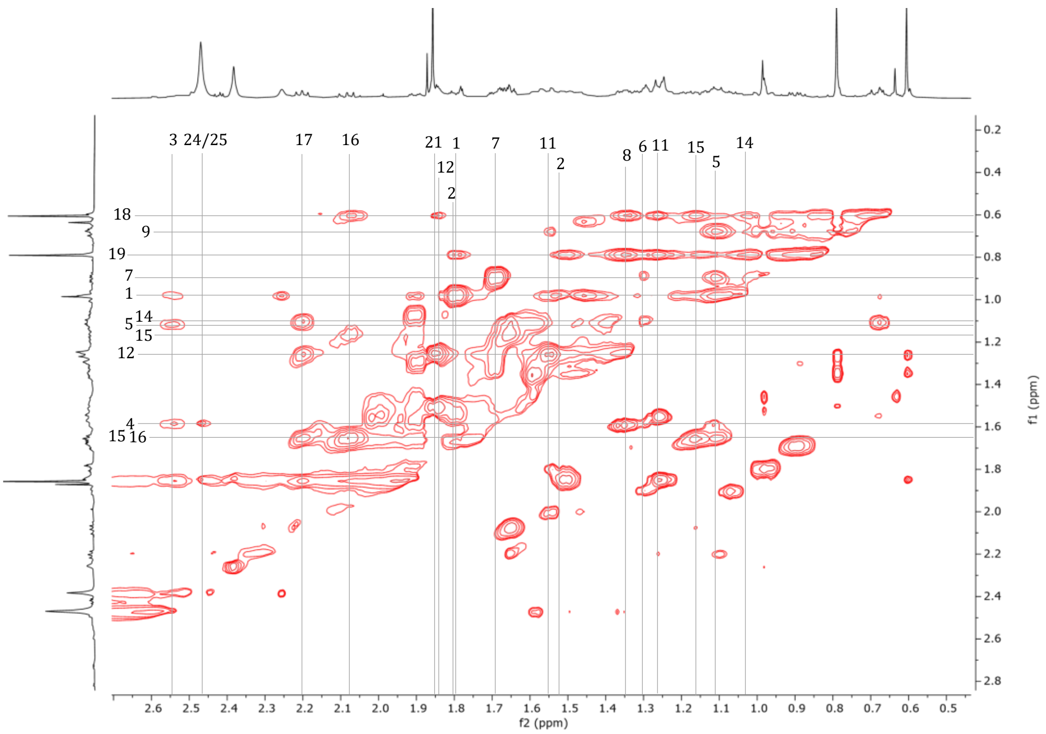

Figure S195. Detail of the $^1H/^1H$ NOESY spectrum of 3β-dimethylamino-pregnane-20-oxime (**19**) and 3β-dimethylamino-pregn-5,6-ene-20-oxime (**20**) ($CDCl_3$, 600 MHz).
